# Engineering CO_2_ Reduction Pathways via Alloy‐Support Interactions in Li‐CO_2_ Batteries

**DOI:** 10.1002/adma.73809

**Published:** 2026-06-20

**Authors:** Liang Sun, Xindan Zhang, Guang Feng, Guoqiang Zhao, Bernt Johannessen, Guanjie Li, Shilin Zhang, Hongge Pan, Zaiping Guo

**Affiliations:** ^1^ Institute of Science and Technology for New Energy Xi'an Technological University Xi'an China; ^2^ School of Chemical Engineering The University of Adelaide Adelaide Australia; ^3^ Department of Beijing Key Laboratory for Chemical Power Source and Green Catalysis Beijing Institute of Technology Beijing China; ^4^ Australian Synchrotron ANSTO Clayton Victoria Australia; ^5^ Institute for Superconducting & Electronic Materials (ISEM) Australian Institute for Innovative Materials (AIIM) Innovation Campus University of Wollongong Wollongong New South Wales Australia; ^6^ Department of Materials Science and Engineering City University of Hong Kong Kowloon Hong Kong China

**Keywords:** CO_2_ redox reactions, electron tuning, Li_2_C_2_O_4_, Li‐CO_2_ batteries, RuCu/NC

## Abstract

Rechargeable Li‐CO_2_ batteries (LCBs) hold great promise for dual‐function CO_2_ utilization and energy storage, yet their practical application is hindered by the sluggish kinetics of the conventional Li_2_CO_3_ pathway, resulting in low discharge voltages (below 2.0 V) and large overpotentials (over 1.0 V). Herein, we propose a strategy of CO_2_ reduction pathway engineering via alloy‐support interaction to unlock high‐performance LCBs. We designed a Ru_2_Cu_4_/NC_1000_ catalyst, where spectroscopy confirms distinct charge redistribution driven by strong coordination between the Ru_2_Cu_4_ alloy and N‐doped support. Theoretical simulations validate that this interaction shifts the Ru and Cu d‐band centers toward the Fermi level and induces interfacial charge redistribution, thus optimizing the electronic structure of the Ru‐Cu active sites for CO_2_ reduction. More importantly, this electronic restructuring thermodynamically favors the formation of metastable Li_2_C_2_O_4_ over insulating Li_2_CO_3_, thus significantly reducing the activation energy barrier for the rate‐determining step by 0.56 eV. As a result, the cell achieves a minimal overpotential of 0.50 V, an exceptional discharge voltage of 3.23 V, and a high specific capacity of 33 922 mAh g^−1^ (at 100 mA g^−1^). Our work establishes electron‐state engineering via alloy‐support interactions as a protocol for directing reaction pathways and achieving high‐voltage and durable LCBs.

## Graphical abstract

Alloy‐support interactions in RuCu/NC induce interfacial charge redistribution and shift d‐band centers, steering CO_2_ reduction from Li_2_CO_3_ to metastable Li_2_C_2_O_4_. This pathway engineering lowers the rate‐determining barrier and suppresses carbonate formation, enabling high discharge voltage (3.23 V) in Li‐CO_2_ batteries with reduced overpotential (0.50 V) and enhanced capacity, highlighting a strategy for efficient CO_2_ utilization and energy storage.

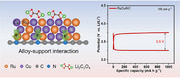

## 1 Introduction

Rechargeable Li‐CO_2_ batteries (LCBs) offer a promising paradigm for integrated high‐density energy storage and CO_2_ utilization. Theoretically, LCBs operate via a four‐electron redox chemistry (4Li^+^ + 3CO_2_ + 4e^−^ → 2Li_2_CO_3_ + C), delivering a high specific capacity of 1876 Wh kg^−1^ at an equilibrium potential of 2.88 V [1, 2]. However, the practical deployment of LCBs is bottlenecked by the inherent chemical inertness of CO_2_, which imposes a high activation energy barrier for CO_2_ reduction. This results in a depressed discharge voltage (often < 2.5 V), and even lower than 2.0 V without efficient catalysts, leading to a significant drop in the actual specific energy and cycle life of a battery [3, 4]. Additionally, the primary discharge product, Li_2_CO_3_, is a wide‐bandgap insulator that passivates the cathode surface, causing sluggish decomposition kinetics. As a result, high charge potentials over 4.3 V are often required, despite the thermodynamic equilibrium potential for the decomposition reaction being only 3.82 V (*vs*. Li/Li^+^) [3]. Notably, these high voltages trigger the irreversible decomposition of Li_2_CO_3_, generating the O_2_^−^ reactive radicals that accelerate electrolyte decomposition and severely compromise the battery reversibility [5, 6, 7, 8, 9].

Alternatively, the two‐electron reduction pathway (2Li^+^ + 2CO_2_ + 2e^−^ → Li_2_C_2_O_4_) represents a thermodynamically favorable route, defined by a high equilibrium potential of 3.01 V (*vs*. Li/Li^+^). Unlike the stable and insulating Li_2_CO_3_, the metastable Li_2_C_2_O_4_ exhibits much lower barriers for electrochemical decomposition. However, stabilizing this metastable product requires precise catalytic modulation. Early attempts using Mo_2_C catalysts proved ineffective in preventing the rapid disproportionation of Li_2_C_2_O_4_, while the use of soluble redox mediators (RMs) introduced the detrimental “shuttle effect”, causing parasitic side reactions at the Li anode [6, 10]. Recently, solid‐state RMs have emerged as a robust solution. For instance, the specific Cu(II) metal‐organic framework of benzene‐1,3,5‐tricarboxylic acid solid RM was demonstrated to retain the high‐voltage efficacy of soluble RMs while confining the redox activity to the cathode [11]. Although this strategy could mitigate the shuttle effect and accelerate kinetics, the synthesis of such ligand‐specific frameworks is inherently complex and synthetically demanding, especially when contrasted with the facile scalability of supported metal catalysts. The practical viability and broader application of the solid RM concept require further experimental validation.

Supported metal catalysts, where active metal species are anchored on robust substances, offer a robust and scalable alternative to complex molecular frameworks, as validated across chemical industries and energy conversion applications. Beyond providing structural integrity, the support plays a key role in stabilizing active metal sites, ensuring high atom utilization efficiency, and even acts as a co‐catalyst in specific reactions [12, 13]. More importantly, the interactions between the metal and the support are instrumental in modulating the local electronic structures and catalytic performance. An example is the widely recognized Strong Metal‐Support Interaction, such as the encapsulation of metal particles (*e.g*., Pt and Ru) by reducible support oxides (*e.g*., TiO_x_), which induces profound electronic and structural changes. This phenomenon allows for the rational tuning of catalytic activity, selectivity, and long‐term durability. However, the strategic application of metal‐support interactions to regulate reaction pathways in LCBs remains underexplored. This is because the complicated multiphase reaction environment makes it challenging to decouple the contributions of geometric and electronic effects. Furthermore, the insulating Li_2_CO_3_ rapidly covers the active sites during cell operation, limiting the intrinsic structure‐activity relationships required to rationally design pathway‐selective catalysts.

To address these challenges, we report the rational design of a ruthenium‐copper alloy supported on nitrogen‐doped carbon (Ru_2_Cu_4_/NC) as a catalyst for LCBs. Using the x‐ray absorption spectroscopy (XAS) and simulation, the introduction of N atom is effective to adjust the electron state of the Ru_2_Cu_4_ alloy via alloy‐support coordination. This approach alters the reaction pathway by suppressing the formation of insulating Li_2_CO_3_ while promoting the formation of metastable Li_2_C_2_O_4_ (Figure S1). As a result, the Ru_2_Cu_4_/NC_1000_ catalyst delivers a high discharge plateau to 3.23 V and an ultralow overpotential of only 0.50 V (at 100 mA g^−1^ with a fixed capacity of 1000 mA g^−1^). Furthermore, the cell using the Ru_2_Cu_4_/NC_1000_ catalyst enables exceptional reversibility, maintaining stable cycling performance for over 1800 h. Notably, the discharge plateau is well maintained at 2.96 V even at an ultrahigh current density of 1000 mA g^−1^, highlighting the outstanding rate capability of the catalyst. Our findings demonstrate that precise control over metal‐support interactions can regulate reaction pathways, validating this concept as a potent strategy for constructing high‐performance, reaction‐selective bimetallic electrocatalysts for LCBs.

## 2 Results and Discussion

### 2.1 Synthesis and Structural Characterization of Ru_2_Cu_4_/NC Catalysts

Ru‐Cu heterostructure alloys were loaded on nitrogen‐doped carbon (NC) supports with tunable nitrogen contents [14, 15]. The NC supports were prepared by thermal condensation of glucose and dicyandiamide at 800°C, 900°C, and 1000°C under a nitrogen atmosphere, and are denoted as NC_800_, NC_900_, and NC_1000_, respectively. X‐ray photoelectron spectroscopy (XPS) measurements (Figures S2 and S3 and Table S2) were used to analyze their chemical composition and nitrogen content of carbon supports. The C *1s* peak located at 285.0 eV for all three samples indicates that the carbon framework is mainly graphitic (*sp^2^*‐hybridized domains). With increasing calcination temperature, the nitrogen content decreases from 14.18 wt.% for NC_800_ to 8.68 wt.% for NC_900_ and further to 6.12 wt.% for NC_1000_, consistent with previous reports [14, 15]. X‐ray diffraction (XRD) patterns (Figure S4), scanning electron microscopy (SEM), and transmission electron microscopy images (TEM, Figure S5) respectively, reveal no obvious differences in the crystalline structure or morphology among NC_800_, NC_900_, and NC_1000_ specimens. In contrast, high‐resolution N *1s* XPS spectra (Figure S3) show that graphitic N becomes the dominant nitrogen species as the calcination temperature increases, as quantified in Figure S3j. This trend is further supported by N K‐edge soft x‐ray absorption spectra (SXAS, Figure S3k), in which the graphitic N feature of NC_1000_ exhibits a higher intensity than those of NC_800_ and NC_900_ [5, 16]. Considering graphitic N can regulate the electron pathways of the carbon framework, which is expected to enhance the electronic conductivity of the carbon support and improve catalytic performance [17]. Additionally, the specific surface area increases from 406.3 m^2^ g^−1^ for NC_800_ to 519.4 m^2^ g^−1^ for NC_1000_ (Figure S6a), while pore size distribution analysis confirms that all NC samples mainly possess mesoporous structures (Figure S6b). Owing to its high surface area, suitable N content, and relatively high graphitization, NC_1000_ was selected as the representative support for the following studies, unless stated otherwise [15].

**FIGURE 1:**
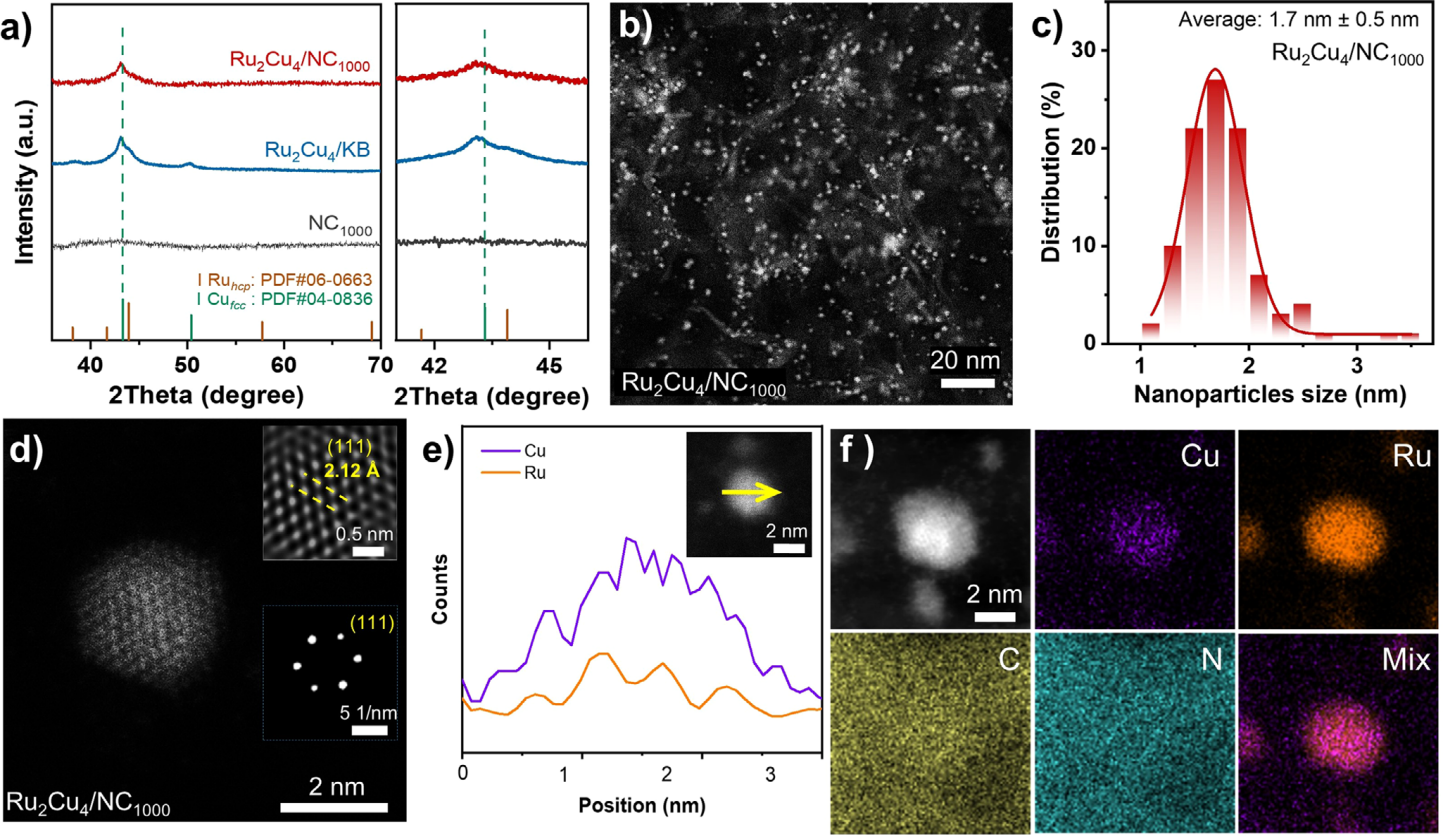
(a) XRD pattern of Ru_2_Cu_4_/NC_1000_, Ru_2_Cu_4_/KB, and NC_1000_ materials. (b) Representative HAADF‐STEM image of the Ru_2_Cu_4_/NC_1000_ catalyst. (c) Size distribution histogram (counted number of particles: 100). (d) Atomically resolved HAADF‐STEM image of a Ru_2_Cu_4_/NC_1000_ particle. The inserts show the fast Fourier transform image and the simulated crystal structure image of the chosen nanoparticle. (e) Line scanning profiles of a single particle in Ru_2_Cu_4_/NC_1000_. (f) HAADF‐STEM image of the chosen area of Ru_2_Cu_4_/NC_1000_ and corresponding elemental distribution mapping images.

Ru‐Cu catalysts with a Ru: Cu atomic ratio of 1:2, denoted as Ru_2_Cu_4_, were synthesized by wet impregnation of soluble Ru and Cu precursors onto NC or Ketjen black (KB) supports, followed by NaBH_4_ reduction and subsequent thermal treatment under an H_2_/Ar mix atmosphere. The Ru‐Cu system was selected based on the high catalytic activity of Ru for CO_2_ redox reactions and the ability of Cu to promote CO_2_ adsorption and activation while reducing noble metal usage [10, 18, 19]. The KB was chosen as an N‐free carbon support to clarify the role of N doping in regulating alloy formation, because KB has a similar porous structure and specific surface area (Figure S7) [20]. The XRD patterns (Figure 1a) of Ru_2_Cu_4_/KB and Ru_2_Cu_4_/NC_1000_ exhibit broad and weak diffraction peaks, indicating that the metal species are present as ultrafine clusters lacking long‐range lattice order. For Ru_2_Cu_4_/NC_1000_, a broad peak centred at 43.1° can be assigned to the *face‐centred cubic* Cu*_fcc_* (111) plane. Compared with the standard Cu*_fcc_* (111) reflection at 43.3°, the slight shift toward lower angles indicates lattice expansion caused by Ru doping due to the large atomic radius of Ru (134 pm) compared to Cu (128 pm), confirming the formation of a RuCu alloy [21]. TEM (Figure 1b,c) images show that RuCu nanoparticles, with an average diameter of about 1.7 ± 0.5 nm, are uniformly dispersed on the NC_1000_ support. High‐resolution TEM further confirms the alloy structure, indicating clear lattice fringes with an interplanar spacing of 2.12 Å (Figure 1d and Figure S8), which is larger than that of metallic Cu (2.09 Å), further indicating homogeneous Ru substitution within the Cu lattice [21, 22]. The alloy composition was further examined by scanning transmission electron microscopy (STEM). Line‐scan profiles collected from individual RuCu alloy particles in Ru_2_Cu_4_/NC_1000_ (Figure 1e and Figure S9) reveal that Ru and Cu are uniformly distributed, with an atomic ratio of about 1:1.89. This value is in agreement with the ratio obtained from XPS results (1:2.17, Figure S10b) and is close to the theoretical Ru: Cu molar ratio of 1:2. The inductively coupled plasma‐atomic (ICP, Table S3) results further confirm a similar composition. High‐angle angular dark‐field STEM (HAADF‐STEM) imaging combined with energy‐dispersive x‐ray spectrometer (EDS) elemental mapping confirms the homogeneous distribution of Ru and Cu within each nanoparticle in Ru_2_Cu_4_/NC_1000_, while nitrogen species remain homogeneously dispersed throughout the carbon support (Figure 1f).

Catalysts with varied Ru and Cu atomic ratios were also prepared and are denoted as Ru*_x_* Cu_6_ *_‐x_*/KB or Ru*_x_* Cu_6_ *_‐x_*/NC_800_ (*x* = 6, 4, 3, 2, 0, where *x* represents the atomic ratio of Ru). These samples were designed to systematically investigate the influence of the Ru/Cu ratio on the physicochemical properties, electronic structure, and corresponding catalytic behavior of the alloys. NC_800_ support was chosen because it offers good structural durability and favorable compatibility with uniform alloy dispersion (Figure S11). For catalysts supported on N‐free KB, XRD patterns (Figure S12) confirm well‐defined diffraction peaks for both Cu_6_/KB and Ru_2_Cu_4_/KB, corresponding to the characteristic *fcc* structure of metallic Cu (PDF#04‐0836). As the Ru atomic fraction increases in Ru*_x_* Cu*_6‐x_*/KB, the diffraction features gradually evolve from the Cu*_fcc_* phase toward the hexagonal close‐packed (*hcp*) phase characteristic of metallic Ru. This phase change is clearly observed in Ru‐dominant phases, including Ru_3_Cu_3_/KB, Ru_4_Cu_2_/KB, and Ru_6_/KB, which indicates pronounced crystallization of Ru‐rich domains on the KB support. In contrast, XRD patterns of the Ru*_x_* Cu*_6‐x_*/NC_800_ material (Figure S13) and Ru_2_Cu_4_/NC_800_, Ru_2_Cu_4_/NC_900_, and Ru_2_Cu_4_/NC_1000_ (Figure S14) exhibit only broad and weak diffraction features, with no distinct reflections corresponding to metallic Ru or Cu. Compared with their Ru*_x_* Cu*_6‐x_*/KB counterparts, the presence of nitrogen species in NC_800_ is effective in suppressing excessive growth and phase separation of Ru and Cu, promoting the formation of uniformly dispersed alloy clusters in the Ru*_x_* Cu*_6‐x_*/NC_800_ materials. Nitrogen‐doped carbon provides abundant coordination sites, including pyridinic, pyrrolic, and graphitic N, which can strongly anchor metal species, inhibit their migration and coalescence, and maintain small particle sizes and high dispersion [23]. TEM images further confirm this finding. As shown in Figures S15 and S16, RuCu alloys in Ru_2_Cu_4_/NC_800_ catalysts are uniformly distributed on NC support with an average particle size of about 1.7 ± 0.5 nm, which is much smaller than that observed for Ru_2_Cu_4_/KB using KB supports (∼2.4 nm).

**FIGURE 2:**
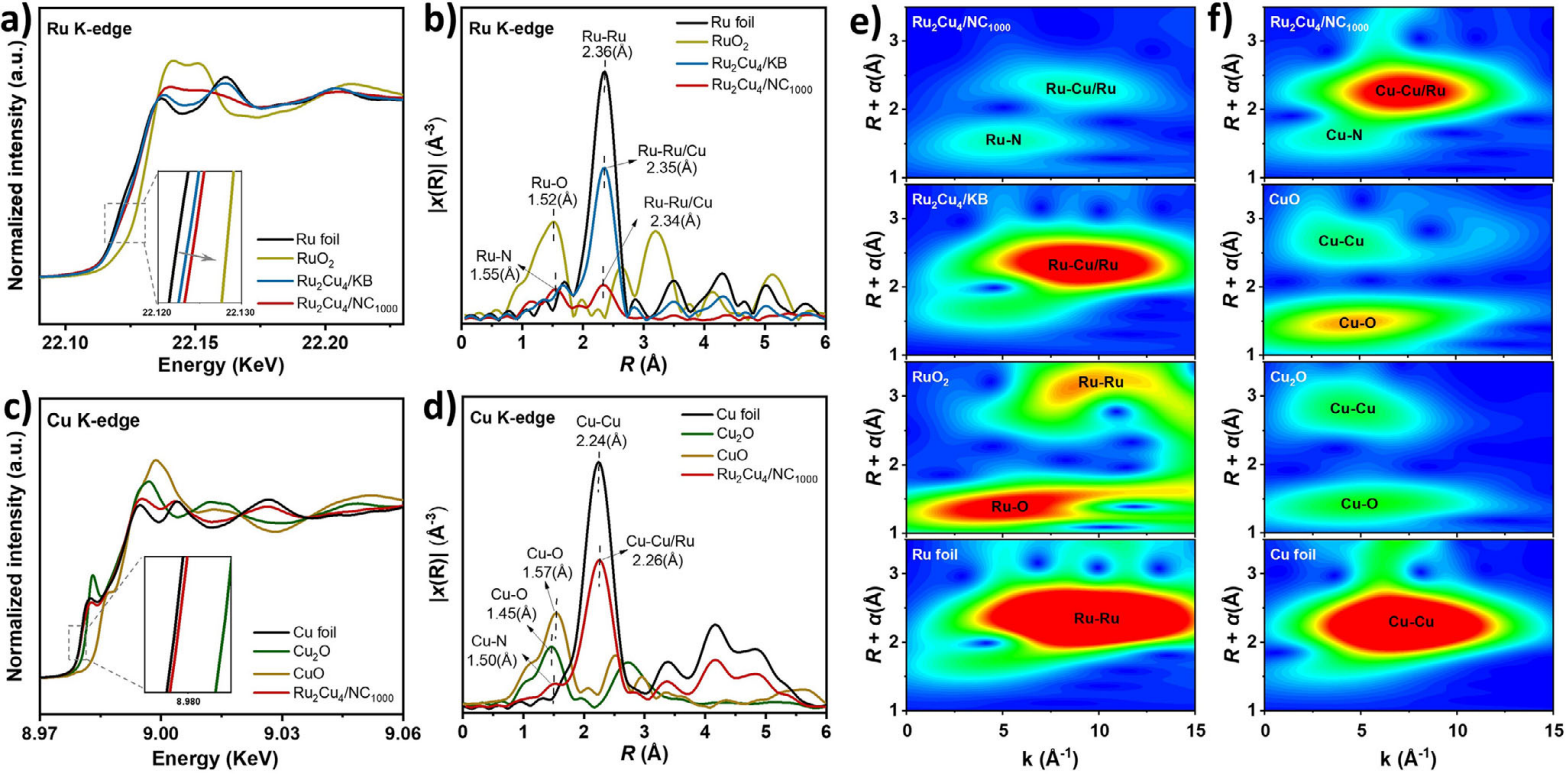
Atomic structure understanding of Ru_2_Cu_4_/NC_1000_ material. X‐ray absorption near edge structure (XANES) spectra of the (a,b) Ru K‐edge and (c,d) Cu K‐edge. (e) Wavelet transformed (WT) of the *k* ^3^‐weighted EXAFS signals of Ru foil, RuO_2_, Ru_2_Cu_4_/KB, and Ru_2_Cu_4_/NC_1000_. (f) WT of the *k* ^3^‐weighted EXAFS signals of Cu foil, Cu_2_O, CuO, and Ru_2_Cu_4_/NC_1000_.

To further understand the electronic structure of the catalysts, Ru and Cu K‐edge XAS, together with x‐ray photoelectron spectroscopy (XPS) measurements, were performed (Figure 2 and Figures S17–S28). XAS was used to investigate both the electronic states and local coordination environment of Ru and Cu. Compared with Ru_6_/NC_800_ and Cu_6_/NC_800_, the Ru and Cu K‐edge x‐ray Absorption Near‐Edge Structure (XANES) spectra of Ru_2_Cu_4_/NC_800_ (Figure S17) show that Ru becomes electron‐enriched, while Cu is electron‐deficient, indicating electron transfer from Cu to Ru due to the high electronegativity of Ru (2.2, Figure S18). XPS results are consistent with the XAS results. As shown in Figures S19 and S20, Ru_2_Cu_4_/NC_800_ exhibits an increased ratio of Ru^0^ species and a positive shift in the Cu *2p* binding energy relative to the corresponding monometallic Cu in Cu_6_/NC_800_ counterpart, indicating electron transfer from Cu to Ru. A similar trend is also observed for Ru_2_Cu_4_/KB (Figure S21), which is in agreement with previous studies reporting charge redistribution from Cu to Ru during RuCu alloy formation and strong electronic coupling between these two metals [22, 24]. In Figure 2a, the white‐line features in the Ru K‐edge XANES spectra of Ru_2_Cu_4_/KB and Ru_2_Cu_4_/NC_1000_ closely resemble those of metallic Ru rather than ruthenium oxides, suggesting that Ru is in the metallic state. This finding is further supported by the Extended x‐ray Absorption Fine Structure (EXAFS) spectra in R space and the first derivatives of the Ru K‐edge XANES profiles (Figure 2b and Figure S22). Notably, compared with Ru_2_Cu_4_/KB, Ru_2_Cu_4_/NC_1000_ exhibits a slight shift of the Ru white line toward higher energy, indicating a change in Ru electron density or the increase of unsaturated orbitals. This shift could be attributed to strong interactions between Ru and the N‐functionalized carbon support [25]. Also, the Cu K‐edge absorption edge of Ru_2_Cu_4_/NC_1000_ is close to that of Cu foil (Figure 2c, inset), suggesting that Cu species are mainly metallic. This result is further confirmed by the first‐order derivatives of Cu K‐edge XANES spectra of Ru_2_Cu_4_/NC_1000_ (Figure S23), which are characteristic of Cu^0^.

To further understand changes in the coordination environment or Ru and Cu, Fourier‐transform EXAFS (FT‐EXAFS) fitting was performed. No metal‐oxygen scattering contributions are observed in the FT‐EXAFS spectra of either Ru_2_Cu_4_/KB or Ru_2_Cu_4_/NC_1000_, indicating no oxidation in both catalysts (Figure 2b,d). In the Ru K‐edge FT‐EXAFS spectra (Figure 2b), the main peak located at ∼2.34 Å for Ru_2_Cu_4_/NC_1000_ is assigned to Ru‐Cu/Ru coordination formed upon incorporation of Ru into the Cu lattice. These metal‐metal distances are slightly shorter than the Ru‐Ru bond length in Ru foil (∼2.36 Å), reflecting lattice distortion associated with alloy formation [26]. In the Cu K‐edge FT‐EXAFS spectra (Figure 2d), Ru_2_Cu_4_/NC_1000_ exhibits a dominant peak at 2.26 Å, which is slightly larger than the Cu─Cu distance in Cu foil (2.24 Å). This finding can be attributed to Cu─Cu and Cu─Ru coordination, further confirming the formation of the Ru─Cu alloy [21]. FT‐EXAFS fitting results provide deeper insight into the local coordination environments (Table S4 and Figures S24 and S25). Ru_2_Cu_4_/KB exhibits Ru─Ru and Ru─Cu coordination numbers of 8.0 and 2.0, respectively, indicating the formation of Ru‐rich clusters. In contrast, for Ru_2_Cu_4_/NC_1000_, the Ru K‐edge reveals Ru─Ru, Ru─Cu, and Ru─N coordination numbers of 3.0, 3.7, and 1.4, respectively, while Cu K‐edge fitting yields Cu─Cu, Cu─Ru, and Cu─N coordination numbers of 6.4, 1.5, and 0.6, respectively. The presence of Ru─N coordination promotes a more uniform dispersion of Ru─Cu species, thus reducing the overall metal‐metal coordination numbers. Additionally, the introduction of N enables the change in electron density of alloys and reflects strong interactions between the alloy and N‐doped carbon, especially for Ru. Specifically, the introduction of N atoms acts as an electron acceptor due to the high electronegativity of N (3.04), leading to a decreased electron density on Ru in Ru_2_Cu_4_/NC_1000_ compared with Ru_2_Cu_4_/KB [27]. This result is consistent with the EXAFS results (Figure 2b). Furthermore, Crystal Orbital Hamilton Population (COHP) analysis (Figure S26) reveals that Ru‐N bonding in Ru_2_Cu_4_‐NC is markedly stronger than Cu─N interaction, as evidenced by pronounced bonding states below the Fermi level and a much more negative integrated COHP (ICOHP) value (−3.09 for Ru‐N vs. −0.09 eV for Cu‐N), indicating that nitrogen preferentially coordinates with Ru and dominantly modulates the electronic structure of the catalyst. This conclusion is fully consistent with the XAS fitting results, which reveal a higher Ru‐N coordination number (1.4) than that of Cu─N (0.6), further confirming the preferential coordination of nitrogen with Ru in Ru_2_Cu_4_‐NC. Notably, the metal‐metal coordination peak for both Ru_2_Cu_4_/KB and Ru_2_Cu_4_/NC_1000_ is significantly weaker than that of the corresponding metal foils, owing to the small cluster size and the absence of long‐range structural periodicity. Compared with metallic Ru and Cu foils, which both exhibit a first‐shell coordination number of 12, the reduced metal‐metal coordination numbers observed for Ru_2_Cu_4_/NC_1000_ and Ru_2_Cu_4_/KB indicate the existence of unsaturated Ru and Cu sites, which could act as the active sites for LCBs.

Wavelet transform (WT) analysis of the Ru K‐edge EXAFS spectra (Figure 2e) provides more information on the local coordination environment of Ru. For Ru_2_Cu_4_/NC_1000_, two distinct intensity maxima are observed: a low‐k feature at *k* = 6.00 Å^−1^ and R = 1.55 Å, which can be assigned to Ru‐N coordination, and a higher‐k feature at *k* = 8.90 Å^−1^ and R = 2.34 Å corresponding to Ru‐Cu/Ru interactions [28]. This WT pattern is markedly different from those of Ru foil and RuO_2_. Specifically, Ru foil exhibits a dominant lobe centered at *k* = 9.70 Å^−1^, characteristic of the metallic Ru‐Ru bond [21]. For Ru_2_Cu_4_/KB and Ru_2_Cu_4_/NC_1000_, the main WT lobes shift to lower *k* values (∼9.20 and ∼8.90 Å^−1^, respectively), which are attributable to mixed Ru─Cu/Ru coordination, reflecting alloy formation and reduced Ru‐Ru coordination. The WT‐EXAFS analysis at the Cu K‐edge further confirms the coordination between Ru and Cu. As shown in Figure 2f, the WT contour plot of Ru_2_Cu_4_/NC_1000_ exhibits a pronounced maximum at *k* = 7.50 Å^−1^ and R = 2.26 Å, characteristic of Cu─Cu/Ru coordination, along with a distinct low‐R feature at *k* = 4.05 Å^−1^ and R = 1.50 Å attributable to Cu‐N bonding. These features differ from the WT signatures of Cu foil and Cu oxides, in which Cu‐Cu (*k* = 7.40 Å^−1^; R = 2.24 Å) and Cu─O (*k* = 5.00 Å^−1^; R = 1.45/1.57 Å) interactions dominate [29]. The observation of Cu─N and the Cu─Cu/Ru signal demonstrates that Ru atoms are coordinated with Cu.

Furthermore, XANES analysis (Figure S27) reveals that nitrogen content plays a key role in regulating the electronic states of Ru in the Ru‐Cu alloy. The negative shift of the Ru white line for Ru_2_Cu_4_/NC_1000_ indicates an increase in Ru electron density as the nitrogen content decreases from NC_800_ to NC_1000_, reflecting weakened electron withdrawal by N species. XPS and N K‐edge XAS spectra (Figure S28) confirm the presence of metal‐N coordination in the Ru─Cu alloy [30]. Notably, Ru_2_Cu_4_/NC_1000_ exhibits a pronounced metal‐N coupling feature, indicating strong alloy‐support interactions and highlighting the combined influence of bimetallic coupling and nitrogen doping on electronic redistribution. Additionally, ultraviolet photoelectron spectroscopy measurements (UPS, Figure S29) show that the work functions of Ru_2_Cu_4_/NC_800_, Ru_2_Cu_4_/NC_900_, and Ru_2_Cu_4_/NC_1000_ are 6.40, 6.14, and 5.80 eV, respectively. The reduced work function of Ru_2_Cu_4_/NC_1000_ indicates a lower barrier for electron transfer from the catalyst surface to adsorbed reactant during catalytic reaction [31]. In short, the unique electronic configuration of Ru_2_Cu_4_/NC_1000_ generates distinct surface electronic environments that could govern the adsorption and activation of reactants, intermediates, and products in LCBs. Beyond charge redistribution, strong metal‐support interactions may further influence the catalytic mechanism by modulating intermediate binding through electronic coupling between metal atoms and the support, as well as by enabling cooperative co‐catalytic interactions in which both the Ru─Cu alloy and N‐doped carbon directly participate in stabilizing reaction species.

### 2.2 Performance of LCBs With Catalysts

We firstly investigated the influence of alloy composition on the catalytic performance of Ru*_x_* Cu_6‐_ *_x_*/NC catalysts in LCBs. A corresponding set of RuCu alloy catalysts supported on different N‐doped carbon supports (NC_800_, NC_900_, and NC_1000_) and N‐free KB was also evaluated. For the KB‐supported catalysts, the introduction of Ru─Cu alloy maintains or even enhances catalytic activity compared with Ru_6_/KB but significantly reduces Ru content. Among them, Ru_2_Cu_4_/KB delivers a low overpotential of 0.96 V when compared to those of Ru_6_/KB (1.06 V) and Cu_6_/KB (1.23 V, Figure S31). Compared with Cu_6_/NC_800_ (1.43 V), Ru_6_/NC_800_ (0.92 V), Ru_4_Cu_2_/NC_800_ (0.9 V), and Ru_3_Cu_3_/NC_800_ (0.93 V), Ru_2_Cu_4_/NC_800_ delivers the lowest overpotential of 0.80 V and maintains a much higher discharge plateau above 3.0 V (Figure S33b,c). In addition to enhanced activity, Ru_2_Cu_4_/NC_800_ exhibits superior cycling stability, sustaining 49 stable cycles, which outperforms Ru_6_/KB (37 cycles) and Cu_6_/KB (48 cycles, Figure S33j). Electrochemical impedance spectroscopy (EIS) data (Figure S34) further demonstrate that Ru_2_Cu_4_/NC_800_ has the lowest charge‐transfer impedance among all NC_800_‐supported catalysts, indicating excellent reaction reversibility in LCBs. These results indicate that Ru_2_Cu_4_ is the optimal alloy composition, highlighting the strong synergistic interaction between Ru and Cu that enhances catalytic efficiency while minimizing the usage of noble Ru metal.

**FIGURE 3:**
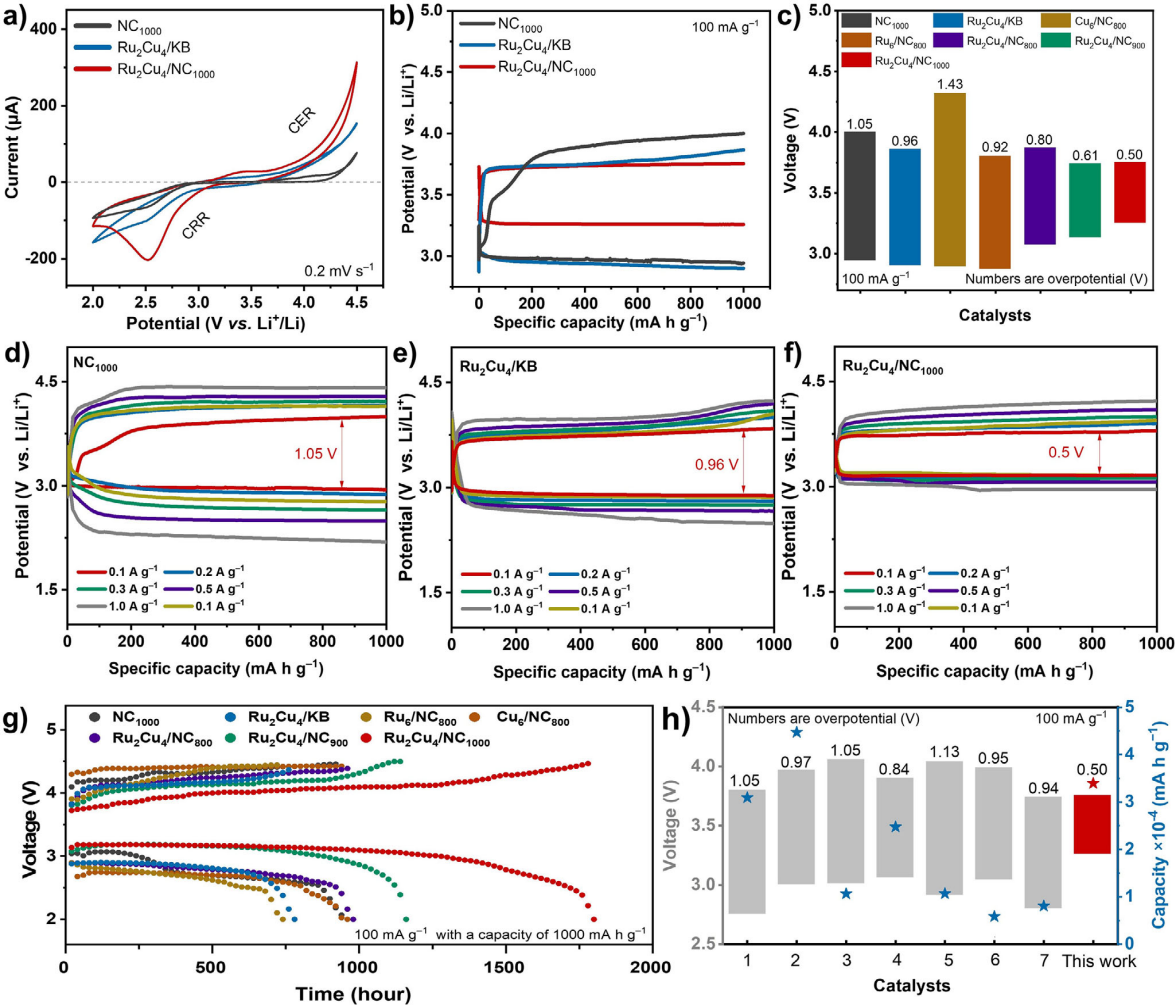
(a) CV curves at 0.2 mV s^‒1^. (b) Charge and discharge curves of NC_1000_, Ru_2_Cu_4_/KB, and Ru_2_Cu_4_/NC_1000_ materials at current densities of 100 mA g^‒1^. (c) Battery overpotentials at current densities of 100 mA g^‒1^. (d–f) Charge and discharge curves of NC_1000_, Ru_2_Cu_4_/KB, and Ru_2_Cu_4_/NC_1000_ materials taken at different current densities. (g) The long cyclic performance of catalysts at 100 mA g^‒1^ curtailed the specific capacity of 1000 mAh g^‒1^. (h) Comparison of the catalysts in this work with the representative Ru systems reported in the literature. 1: SA Ru–Co_3_O_4_/CC (0.1, 0.5) [32]; 2: SA Ru_h_‐NC@rGO (0.1, 1) [18]; 3: Ru_AC+SA_@NDCN (0.1, 1) [28]; 4: Ru/Co‐CPY@CNT (0.1, 1) [33]; 5: Ru/NS‐G (0.1, 1) [34]; 6: Cu(I) RM and Ru catalysts (0.1, 1) [6]; 7: RuCo NSs/CNT (0.1, 1) [35]. The detailed data are provided in Table S6. The testing conditions of overpotential are shown in the parentheses (current density: A g^−1^, cut‐off capacity: Ah g^−1^).

The electrochemical performance of Ru_2_Cu_4_/NC_800_, Ru_2_Cu_4_/NC_900_, and Ru_2_Cu_4_/NC_1000_ was further conducted to indicate how the N content in the NC support influences the catalytic performance of Ru_2_Cu_4_ alloy in LCBs. Cyclic voltammetry (CV) measurements were conducted between 2.0 and 4.5 V (Figure 3a and Figure S35a) to probe the electrochemical reaction and related kinetics. Using the Ru_2_Cu_4_/NC_1000_ as the catalyst, distinct redox peaks associated with CO_2_ reduction and the decomposition of discharge products are clearly observed. In comparison with pristine NC_1000_ (2.51 V cathodic, 4.07 V anodic), Ru_2_Cu_4_/KB cathodes (2.49 V cathodic, no clear anodic peak), Ru_2_Cu_4_/NC_800_ (2.43 V cathodic, no clear anodic peak), and Ru_2_Cu_4_/NC_900_ (2.44 V cathodic, no clear anodic peak), the Ru_2_Cu_4_/NC_1000_ cell exhibits a higher cathodic onset potential of 2.52 V and a much lower anodic onset potential of 3.42 V, together with much larger peak currents. These features indicate that the Ru_2_Cu_4_/NC_1000_ has accelerated kinetics for both the CO_2_ reduction reaction (CRR) and the CO_2_ evolution reaction (CER). Additionally, the Ru_2_Cu_4_/NC_1000_ cell delivers a high discharge capacity of 33,922 mA h g^−1^, with 16,067 mA h g^‒1^ contributed above 3.0 V, indicating the high catalytic performance for CRR (Figure S35b). As shown in Figure 3b, when compared to the pristine NC_1000_ catalyst, Ru_2_Cu_4_/NC_800_, Ru_2_Cu_4_/NC_900_, and Ru_2_Cu_4_/NC_1000_ cells all exhibit enhanced CRR activity, maintaining discharge plateaus above 3.0 V at a current density of 100 mA g^‒1^. Notably, Ru_2_Cu_4_/NC_1000_ achieves a discharge voltage of 3.23 V, which is much higher than values reported in most previous studies [6]. As shown in Figure 3c, the cell using Ru_2_Cu_4_/NC_1000_ catalyst exhibits a low overpotential of 0.5 V at a current density of 100 mA g^‒1^ with a cut‐off capacity of 1000 mAh g^‒1^, which is much lower than those of cells using pristine NC_1000_ (1.05 V), Ru_2_Cu_4_/KB (0.96 V), Ru_2_Cu_4_/NC_800_ (0.80 V), and Ru_2_Cu_4_/NC_900_ (0.61 V). With increasing current density from 100 to 200, 500, and 1000 mA g^‒1^, the discharge termination voltage of the Ru_2_Cu_4_/NC_1000_ cell drops slightly from 3.25 to 3.14, 3.07, and 2.96 V, respectively (Figure 3f), indicating minimal voltage polarization for CRR. In contrast, pristine NC_1000_, Ru_2_Cu_4_/KB, Ru_2_Cu_4_/NC_800_, and Ru_2_Cu_4_/NC_900_ show significantly larger polarization under identical conditions (Figure 3d, e, and Figure S36 and Table S4), reflecting the inhibited reaction kinetics.

Long‐term cycling tests further confirm the superior durability of Ru_2_Cu_4_/NC_1000_. When cycled at 100 mA g^‒1^ with a cut‐off capacity of 1000 mAh g^‒1^, the cell using Ru_2_Cu_4_/NC_1000_ catalyst delivers stable operation for 90 cycles (1800 h), while maintaining low overpotential and stable discharge‐charge profiles (Figure 3g). Even when operated at a high current density of 500 mA g^‒1^, the cell using Ru_2_Cu_4_/NC_1000_ catalyst retains a low overpotential of 0.89 V and maintains a long cycling life of 88 cycles (352 h, Figure S37). Our findings indicate that the cell using Ru_2_Cu_4_/NC_1000_ catalyst achieves an overpotential of 0.50 V, which is significantly lower than that of most previously reported solid‐state catalysts in LCBs (Figure 3h). These findings demonstrate that supporting Ru_2_Cu_4_ alloys on the NC_1000_ matrix enhances catalytic activity in LCBs. This performance enhancement originates from differences in electronic characteristics among these catalysts, which are systematically regulated by N functionalities in the NC support. The resulting electronic modulation accelerates both CRR and CER by improving charge‐transfer kinetics [36]. Such regulation is expected to modify the adsorption strengths of CO_2_ and key discharge intermediates, thus governing reaction pathways and product desorption behavior. This could be reflected in the change of the adsorption strengths of CO_2_, intermediates, and discharge products, thus influencing reaction pathways, which will be discussed in the following part.

### 2.3 Electrochemical Reaction Mechanism

To elucidate the mechanisms, cathodes at different reaction stages, including the initial state, after the first discharge, and after the first recharge, were examined. EIS data collected after the discharge test and corresponding discharge profiles are shown in Figure S38. For catalysts supported on NC_800_, clear differences in charge‐transfer behavior are observed among the monometallic and alloy catalysts. Specifically, the Cu_6_/NC_800_ cell exhibits a moderate charge‐transfer resistance of ∼1880 Ω, while the Ru_6_/NC_800_ cell shows a much higher resistance of about 2460 Ω. In contrast, the cell using Ru_2_Cu_4/_NC_800_ displays a substantially lower resistance of ∼850 Ω, demonstrating that Ru─Cu alloying markedly enhances charge transport. Additionally, Ru_2_Cu_4/_NC_800_, Ru_2_Cu_4/_NC_900_, and Ru_2_Cu_4/_NC_1000_ cells all exhibit relatively low resistances below 1000 Ω, especially the cell using Ru_2_Cu_4/_NC_1000, which_ shows the lowest resistance of about 375 Ω, reflecting the superior electronic conductivity and interfacial properties tuned by the N characteristics. In contrast, Ru_2_Cu_4_/KB exhibits a much higher resistance of ∼2070 Ω. These results demonstrate that the NC_1000_, owing to its abundant graphitic N content and favorable electronic structure, serves as a favorable support for Ru_2_Cu_4_ alloy, thus enabling the fastest interfacial charge transfer and catalytic kinetics.

**FIGURE 4:**
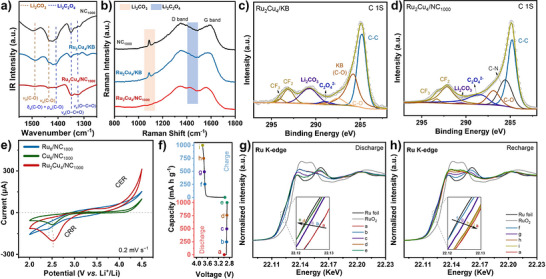
(a) FTIR and (b) Raman of NC_1000_, Ru_2_Cu_4_/KB, and Ru_2_Cu_4_/NC_1000_ cathodes after the first discharge taken at current densities of 100 mA g^‒1^ with a limited capacity of 1000 mA g^‒1^. XPS C *1s* spectra of (c) Ru_2_Cu_4_/KB cathode and (d) Ru_2_Cu_4_/NC_1000_ cathode after the first discharge. (e) CV curves at 0.2 mV s^‒1^. (f) The charge and discharge curves of Ru_2_Cu_4_/NC_1000_ cathode during the first cycle at current densities of 100 mA g^−1^ with a limiting capacity of 1000 mAh g^−1^. (g,h) In situ XANES spectra for Ru K‐edge in Ru_2_Cu_4_/NC_1000_ at different first discharging and first charging states in fluorescence mode.

To identify the discharge products formed on different cathode materials, the composition and morphology of the cathodes were analyzed at the first discharge stage. Fourier transform infrared (FTIR) spectroscopy confirms distinct discharge products depending on both the catalyst composition and the carbon support. For Ru_6/_NC_800_, Cu_6/_NC_800_, and Ru_2_Cu_4/_NC_800_, strong adsorption bands at 859 cm^−1^ (*δ*_a_(CO_3_)) and 1425/1485 cm^−1^ (*ν*_a_(C─O)) are observed, which are characteristic of Li_2_CO_3_ (Figure S39). In addition, peaks at 790, 1312/1350, 1607, and 1642 cm^−1^ can be assigned to the *δ*_a_(O−C═O), *ν*_a_(O─C═O), *δ*_s_(C─O) + *ρ*_ω_(C─O), and *ν*_a_(C═O) modes of Li_2_C_2_O_4_, respectively [37, 38]. The coexistence of Li_2_CO_3_ and Li_2_C_2_O_4_ indicates that these catalysts are capable of stabilizing Li_2_C_2_O_4_ during the discharge process. Importantly, the Ru_2_Cu_4_/NC_1000_ cell (Figure S39) shows that the relative intensity of Li_2_C_2_O_4_‐related peaks is increased when compared to those in Ru_2_Cu_4_/NC_800_ and Ru_2_Cu_4_/NC_900_, while Ru_2_Cu_4_/KB mainly produces Li_2_CO_3_, with only trace amounts of Li_2_C_2_O_4_ detected (Figure 4a). As shown in Figure 4b, Raman spectra collected from the Ru_2_Cu_4_/NC_1000_ cathode after the first discharge reveal a band at 1460 cm^−1^, characteristic of Li_2_C_2_O_4_ formation. In contrast, NC_1000_ and Ru_2_Cu_4_/KB predominantly exhibit a Raman feature at 1089 cm^−1^, which is assigned to Li_2_CO_3_ [6]. XRD and KMnO_4_ titration suggest the presence of Li_2_C_2_O_4_ is a major discharge product on Ru_2_Cu_4_/NC_1000_, consistent with the proposed CO_2_ reduction pathway (Figure S40). These results indicate that the Ru_2_Cu_4_/NC_1000_ catalyst is effective in stabilizing Li_2_C_2_O_4_ as a discharge product, highlighting the critical role of metal‐support interactions and electronic modulation in governing the discharge product.

XPS fitting has been widely used to analyze discharge products in LCBs [6, 11, 39]. A quantitative comparison of discharge products was performed based on carbon species identified from XPS fitting results, which provides surface‐sensitive information within a few nanometers of the cathode surface [39]. As shown in Figure 4c,d and Figures S41–S43, compared with Ru_6_/NC_800_, which contains 12% Li_2_C_2_O_4_, and Cu_6_/NC_800_, which contains 30.4% Li_2_C_2_O_4_, Ru_2_Cu_4_/NC_800_ exhibits a higher Li_2_C_2_O_4_ ratio of 31.7%, indicating that Ru_2_Cu_4_ active sites is effective to stabilize Li_2_C_2_O_4_ as the discharge product. The influence of the carbon support is further highlighted when comparing different Ru_2_Cu_4_‐based catalysts. The cell using Ru_2_Cu_4_/NC_1000_ exhibits the highest Li_2_C_2_O_4_ proportion of 97.0% (Figure S43), while the Ru_2_Cu_4_/KB cell shows only 11.0% of Li_2_C_2_O_4_. This difference demonstrates that N‐doped carbon supports play a crucial role in promoting Li_2_C_2_O_4_ formation. After the recharge process (Figures S44 and S45), for Cu_6_/NC_800_ and Ru_6_/NC_800_ cells, discharge products are observed with a ratio of 12.5% and 8.3%, respectively. In contrast, Ru_2_Cu_4_/NC_800_ retains only 4.4% discharge products, indicating a superior ability to decompose discharge products during charging. Comparing different carbon supports further highlights the role of nitrogen doping., Ru_2_Cu_4_/KB still retains 7.0% of discharge products, which is much higher than that of Ru_2_Cu_4_/NC, indicating the N incorporation promotes the product decomposition. Among the N‐doped samples, Ru_2_Cu_4_/NC_1000_ exhibits the lowest final total product (2.0%), followed by Ru_2_Cu_4_/NC_900_ (3.6%) and Ru_2_Cu_4_/NC_800_ (4.4%), confirming the excellent catalytic activity and reversibility of Ru_2_Cu_4_/NC_1000_ catalyst (Figure S45).

We further examined the morphology of the discharge products to understand their role in electrochemical performance. After the first discharge, the cell using pristine NC_1000_ is covered with rhombohedral block‐like particles with sizes of about 0.6 µm (Figure S46b), while Ru_2_Cu_4_/KB exhibits extensive polymer‐like surface coatings (Figure S46e). Such continuous blocky particles and dense films can impede ion and electron transport as well as gas diffusion, leading to increased voltage polarization and poor cycling stability in LCBs [40]. In contrast, the Ru_2_Cu_4_/NC_1000_ cathode shows loosely distributed acicular discharge products with about 50 nm in diameter (Figure S46h). This nanoscale morphology suggests that the discharge products can be decomposed more readily during charging, thus contributing to improved reversibility, as evidenced in XPS fitting results. The formation and conversion of discharge products were further verified by examining the corresponding cathode surface, as shown in Figure S46. Compared with other catalysts, the discharge products on the Ru_2_Cu_4_/NC_1000_ cathode exhibit better reversibility and a more uniform and loose distribution. This morphology ensures improved contact with the Ru_2_Cu_4_/NC_1000_ conductive network, reduces ionic transfer pathways, and thus enables a higher discharge capacity.

To better identify the genuine active sites, CV curves of Cu_6_, Ru_6_, and Ru_2_Cu_4_ on the same NC_1000_ support (Figure 4e) were conducted and compared. Distinct cathodic features are observed for three catalysts. Specifically, Cu_6_/NC_1000_ exhibits a reduction peak at a more positive potential of 2.50 V, when compared to that of Ru_6_/NC_1000_ (2.42 V), indicating that Cu more readily initiates CO_2_ conversion. However, the smaller peak area of Cu_6_/NC_1000_ suggests slower overall reaction kinetics compared to Ru_6_/NC_1000_. Notably, Ru_2_Cu_4_/NC_1000_ displays a cathodic peak at 2.51 V with significantly larger peak areas, indicating superior redox behavior arising from Ru‐Cu interactions. These peak shifts demonstrate that the excellent catalytic performance of Ru_2_Cu_4_/NC_1000_ originates from the synergistic coupling of Ru and Cu active sites, rather than the isolated contributions of single‐metal catalysts [39]. This result is further supported by the XPS analysis of the Cu *2p* region for the first discharged cathode (Figure S47). After the first discharge, the Cu *2p* peaks shift toward higher binding energies, accompanied by the emerging of a new satellite feature near 944.0 eV. This change may indicate further oxidation of Cu^0^ [41]. Such electron‐deficient Cu sites could serve as active sites to enhance the adsorption of oxalate species, thus facilitating the formation of Li_2_C_2_O_4_ [11, 39]. Notably, after the recharge process, the Cu *2p* peaks shift back toward lower binding energies and the satellite peak at 944.0 eV disappears, indicating that Cu is back to its original electronic state.

Additionally, in situ XAS of Ru_2_Cu_4_ /NC_1000_ at the Ru K‐edge was collected during both first discharge and charge cycles to further understand the dynamic evolution of Ru sites [42, 43]. The Ru K‐edge XAS spectra collected at different depths of discharge (DoD) and states of charge (SoC) were compared with those of the Ru foil (Figure S48 and Figure 4f). As depicted in Figure 4g,h, the overall features, particularly the white‐line positions of metallic Ru sites in the Ru K‐edge XANES spectra, remain well preserved throughout the entire electrochemical cycling, indicating high structural stability of the Ru sites. During the first discharge, the Ru absorption edge shifts to lower energy relative to the pristine state, indicating that Ru gained numerous electrons, and a large number of CO_2_ and discharge intermediates adsorbed on the surface of Ru sites [44, 45]. This electron gain was further confirmed by the reduced white line intensity. During the first charging, the Ru absorption edge shifted to higher energy compared with the pristine state, suggesting increased oxidation of the Ru surface in contact with charge intermediates and discharge products [44]. At the end of the first charge, the Ru K‐edge position fully recovers to that of the pristine state (Figure 4h, insert), demonstrating excellent reversibility of the Ru sites in Ru_2_Cu_4_/NC_1000_ during cell cycling. The combined in situ XAS and ex situ XPS analyses confirm that both Ru and Cu function as active sites, synergistically enhancing CRR and CER activity.

**FIGURE 5:**
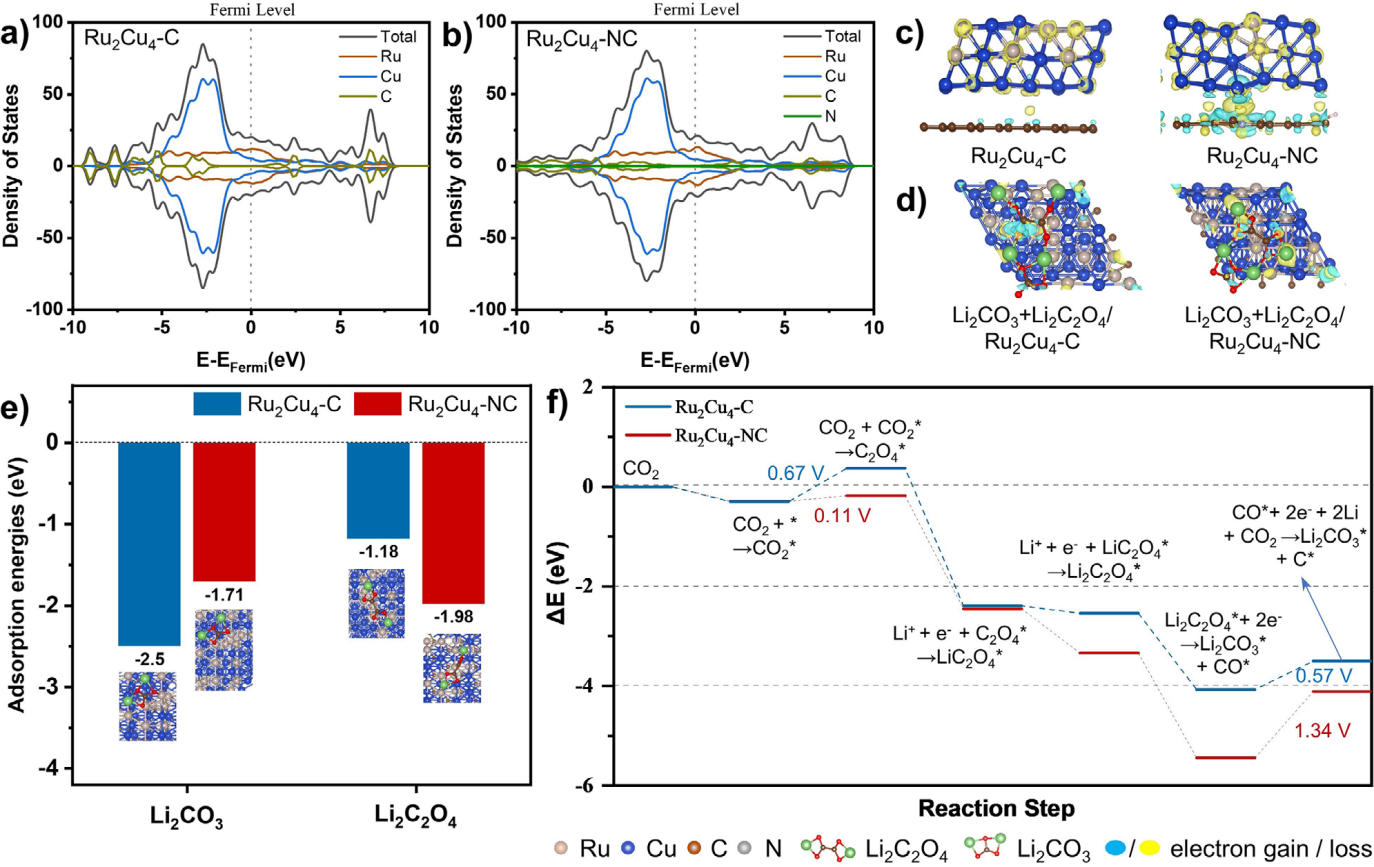
Spin‐polarized projected density of states (PDOS) of (a) Ru_2_Cu_4_‐C and (b) Ru_2_Cu_4_‐NC. (c) The differential charge density diagram of the electrode alloy part and the carbon layer. (d) The differential charge density diagram of co‐adsorbed Li_2_CO_3_ and Li_2_C_2_O_4_ on Ru_2_Cu_4_‐C and Ru_2_Cu_4_‐NC surface. (e) The adsorption energies for Li_2_CO_3_ and Li_2_C_2_O_4_ species on Ru_2_Cu_4_‐C and Ru_2_Cu_4_‐NC surface. (f) A free energy diagram of Ru_2_Cu_4_‐C and Ru_2_Cu_4_‐NC under the equilibrium potentials of Li^+^|Li_2_CO_3_.

Density functional theory (DFT) calculations were further performed to gain a deep understanding of the enhanced catalytic performance of the Ru_2_Cu_4_ supported on N‐doped carbon matrix (Figure 5). Eight quasi‐random Ru: Cu = 1:2 configurations were constructed on a Cu*_fcc_* (111) 3 × 3 × 2 slab. Among them, the eighth configuration (Structure 8) exhibits the lowest energy and was therefore selected as the optimized structural model for subsequent alloy‐carbon interface calculations (Figure S50).

Spin‐polarized projected density of states (PDOS, Figure 5a,b) reveals that the introduction of N in Ru_2_Cu_4_‐NC induces only minor perturbations to the C‐derived states but significantly modulates the d‐band electronic structure of both Ru and Cu. Specifically, the d‐band centers of Ru and Cu shift closer to the Fermi level upon N doping (Table S7), which indicates that it is related to enhanced interactions between the metal active sites and reactants, intermediates, and discharge products, thus favoring catalytic activity. Charge density difference analysis provides further insight into the role of nitrogen doping (Figure 5c). On N‐free carbon, electrons primarily transfer from the carbon matrix to the Ru_2_Cu_4_ alloy surface, while after N doping, pyridinic‐N sites can withdraw electron density from the Ru_2_Cu_4_ alloy owing to the high electronegativity of N, while the surrounding carbon matrix remains electron‐rich and continues to donate electrons to the alloy. This competing charge redistribution results in a unique electronic modulation at the alloy‐support interface that is fundamentally distinct from that on N‐free carbon. In addition, N doping induces minor but identifiable changes in the local RuCu alloy geometry, consistent with coordination rearrangements near nitrogen sites. Our findings indicate that N doping simultaneously modulates the electronic structure and local geometry of the Ru‐Cu alloy, thus optimizing the d‐orbital distribution of the active sites for both CRR and CER.

To further understand the influence of N‐doping in the adsorption behavior of reaction, intermediates, and discharge products, adsorption‐energy calculations were performed (Figure 5e). For Ru_2_Cu_4_‐NC, the adsorption energy of Li_2_C_2_O_4_ is calculated to be −1.98 eV, which is much stronger than that of Li_2_CO_3_ (−1.71 eV). This preferential interaction stabilizes Li_2_C_2_O_4_ on the catalyst surface, resulting in a much higher discharge plateau during discharging. In contrast, Ru_2_Cu_4_‐C exhibits much stronger binding toward Li_2_CO_3_ (−2.50 eV) than Li_2_C_2_O_4_ (−1.18 eV), suggesting that Li_2_CO_3_ is more readily formed on N‐free carbon, consistent with the experimental observation that Ru_2_Cu_4_/NC produces more Li_2_C_2_O_4_ than Ru_2_Cu_4_/KB. Charge density difference analysis (Figure 5d) provides further evidence of this trend. Compared with the N‐free carbon support, the N‐doped Ru_2_Cu_4_‐NC model exhibits a larger and more widely distributed charge‐accumulation isosurface upon Li_2_C_2_O_4_ adsorption. Notably, in Ru_2_Cu_4_‐NC, the accumulated charge is predominantly localized on the C‐O bonds of the C_2_O_4_, whereas in Ru_2_Cu_4_‐C, a substantial fraction of charge density is instead distributed around Li atoms. This indicates more pronounced electron transfer from Ru_2_Cu_4_‐NC to the C_2_O_4_ unit in Li_2_C_2_O_4_, consistent with the enhanced electronic interaction and higher selectivity toward Li_2_C_2_O_4_ formation on N‐doped carbon. The computed reaction energy profiles provide further evidence (Figure 5f). Along the CO_2_ → Li_2_C_2_O_4_* reaction pathway, both catalysts share the same rate‐determining step, namely, CO_2_ + CO_2_* → C_2_O_4_*. For Ru_2_Cu_4_‐NC, the N doping can significantly lower the corresponding energy barrier from 0.67 eV on Ru_2_Cu_4_‐C to 0.11 eV on Ru_2_Cu_4_‐NC, indicating that N‐modified Ru_2_Cu_4_ sites are effective in promoting Li_2_C_2_O_4_ formation. In contrast, for the competing pathway, that is, CO* + 2e^−^ + 2Li^+^ + CO_2_ → Li_2_CO_3_* + C*, the N‐doped model exhibits a much higher energy of 1.34 eV than the Li_2_C_2_O_4_ formation (0.11 eV), indicating suppression of Li_2_CO_3_ formation; moreover, Ru_2_Cu_4_‐C intrinsically favors Li_2_CO_3_ generation because its barrier for Li_2_CO_3_* formation (0.57 eV) is slightly lower than that for Li_2_C_2_O_4_* formation (0.67 eV). These theoretical insights provide a consistent explanation for the experimentally observed enhancement in Li_2_C_2_O_4_ selectivity and the reduced accumulation of Li_2_CO_3_ on Ru_2_Cu_4_/NC_1000_ catalyst, as observed in electrochemical performance.

## 3 Conclusion

In conclusion, this work demonstrates that alloy‐support interaction is an effective method for optimizing CO_2_ reduction pathways in LCBs. By regulating the electronic states of Ru and Cu through nitrogen coordination, the Ru_2_Cu_4_/NC_1000_ catalyst establishes a distinct electronic configuration that promotes Li_2_C_2_O_4_ formation while suppressing the insulating Li_2_CO_3_ pathway. Spectroscopic analyses reveal Ru─Cu charge redistribution and solid‐solution formation with under‐coordinated metal sites, and theoretical calculations further confirm d‐band center upshifts and interfacial charge reorganization that lower the kinetic barriers for C_2_O_4_* formation. As a result, Ru_2_Cu_4_/NC_1000_ achieves a high discharge voltage of 3.23 V and an ultralow overpotential of 0.50 V, while maintaining stable cycling performance for over 1800 h at 100 mA g^−1^. These findings establish electron‐state engineering via alloy‐support interactions as a general strategy for achieving high‐discharge voltage, reversible, and durable LCBs and provide broader insights into catalyst design for multielectron CO_2_ conversion systems.

## Author Contributions

L.S. conducted the experiments and drafted the original manuscript. X.Z., G.Z., and G.L. conducted part of the cathode characterizations and analysed the data. B.J. provided helpful XAS testing. G.F., S.Z., H.P., and Z.G. directed the project and revised the manuscript.

## Conflicts of Interest

The authors declare no conflicts of interest.

## Supporting information




**Supporting File**: adma73809‐sup‐0001‐SuppMat.pdf.

## Data Availability

The data that support the findings of this study are available from the corresponding author upon reasonable request.
