# Supplementary material for: Engineering CO2 Reduction Pathways via Alloy‐Support Interactions in Li‐CO2 Batteries
Source: Adv Mater. 2026 Jun 20;38(42):e73809. doi: 10.1002/adma.73809 (PMC13410828; doi:10.1002/adma.73809)
Supplement: Supplementary file 1 — Supporting File: adma73809‐sup‐0001‐SuppMat.pdf. [file ADMA-38-e73809-s001.pdf]

Supporting Information

Engineering CO_2 Reduction Pathways via Alloy-Support Interactions in Li-CO_2 Batteries

Liang Sun[a,b], Xindan Zhang[b], Guang Feng[c,], Guoqiang Zhao[a], Bernt Johannessen[d,e], Guanjie Li[b], Shilin Zhang[b,*], Hongge Pan[a,*], Zaiping Guo[b,f,*]*

a. *Institute of Science and Technology for New Energy, Xi'an Technological University, Xi'an 710021, China*

b. *School of Chemical Engineering, The University of Adelaide, Adelaide, 5000, Australia*

c. *Department of Beijing Key Laboratory for Chemical Power Source and Green Catalysis, Beijing Institute of Technology, Beijing, 100081, China*

d. *Australian Synchrotron, ANSTO, Clayton, VIC 3168, Australia*

e. *Institute for Superconducting & Electronic Materials (ISEM), Australian Institute for Innovative Materials (AIIM), Innovation Campus, University of Wollongong, Wollongong, NSW 2500, Australia*

f. *Department of Materials Science and Engineering, City University of Hong Kong, Kowloon, Hong Kong 999077, China*

Corresponding author: shilin.zhang01@adelaide.edu.au (S.Z); hgpan@zju.edu.cn (H.P); fg@bit.edu.cn (G.F); zaipiguo@cityu.edu.hk (Z.G)

Methodology

Materials

Ruthenium (III) chloride hydrate ($RuCl_3 \cdot xH_2O$, ≥99.9%), copper (II) sulfate pentahydrate ($CuSO_4 \cdot 5H_2O$, ≥98.0%), sodium borohydride ($NaBH_4$, 99%), dicyandiamide (DCDA, 99.5%), D-(+)-glucose (99.5%), and nafion solution (5% in lower aliphatic alcohols and water, contains 15-20% water) were ordered from Sigma-Aldrich. Ketjen black (KB) was purchased from Fuel Cell Store. All chemicals were analytical grade and used as received without further purification.

Synthesis of NC_z

To prepare the precursor solution, 40 g of DCDA and 1 g of glucose were dissolved in 200 ml of deionized water. The resulting homogeneous solution was heated and stirred until evaporation and maintaining in the vacuum oven for overnight at 50°C, leaving a powder that was subsequently heat-treated at 800 °C, 900 °C, and 1000 °C for 2 hours, with a heating rate of 2 °C/min, under a nitrogen atmosphere. After cooling to room temperature, the resulting black powders were labelled NC_z (where z denotes the heating temperature).

Synthesis of Ru_xCu_{6-x}/NC_z and Ru_xCu_{6-x}/KB

In a typical synthesis of Ru_xCu_{6-x}/NC_{800} (where *x* denotes atomic ratios of Ru) or Ru_xCu_{6-x}/KB, $RuCl_3{\cdot}xH_2O$ (0.06, 0.04, 0.03, 0.02, and 0 mmol) and $CuSO_4{\cdot}5H_2O$ (0, 0.02, 0.03, 0.04, and 0.06 mmol, see Table S1) were first dissolved in deionized water (20 mL). The solution was stirred until a clear solution was obtained. Then, carbon powders (NC_{800} or KB, 20mg) were slowly added to the clear solution, and the mixture was continuously stirred to achieve a uniform dispersion. Subsequently, a saturated aqueous solution of sodium borohydride was added to the solution. Vigorous stirring using a magnetic stirring apparatus was carried out for 2 hours at room temperature. The reaction products were then separated through filtration and subjected to drying in a vacuum oven for 12 hours. Finally, the solid products obtained were annealed at 350°C for 3 hours to obtain the final product.[1]

Synthesis of Ru_2Cu_4/KB in 1.8 nm

To further evaluate the influence of particle size on product selectivity, a size-controlled Ru_2Cu_4/KB catalyst (~1.8 nm), comparable to Ru_2Cu_4/NC_{1000} with an ultrasmall alloy particle size (~1.7 nm), was prepared using a modified polyol (ethylene glycol) reduction method adapted from previous reports.[2] In this approach, metal precursors were reduced in an ethylene glycol system with controlled alkalinity to achieve uniform and ultrasmall nanoparticle dispersion on the KB support. In a typical synthesis, 0.02 mmol of $RuCl_3{\cdot}xH_2O$ and 0.04 mmol of $CuSO_4{\cdot}5H_2O$ were dissolved in 20 mL of deionized water. Then, 20 mg of carbon powders (KB) were slowly added, and the mixture was stirred continuously to ensure homogeneous dispersion. To control the pH and metal nanoparticle formation, 300 µL of 1 M NaOH was added. The resulting mixture was stirred and refluxed at 170 °C for 3 hours to reduce Ru^{3+} and Cu^{2+} to metallic nanoparticles on the carbon support. The resulting Ru_2Cu_4/carbon powders were washed with water and ethanol via three cycles of centrifugation, followed by drying in a vacuum oven at 70 °C overnight.

Battery Assembly and Electrochemical Measurements

The NC_z, Ru_xCu_{6-x}/NC_z and Ru_xCu_{6-x}/KB synthesized in this study were employed as catalysts for electrochemical evaluations in Li-CO_2 batteries (LCBs). For instance, to prepare the catalyst ink for Ru_xCu_{6-x}/NC_z, to prepare the catalyst ink for battery assembly, the Ru_xCu_{6-x}/NC_z was dispersed in an appropriate quantity of Nafion and ethanol. the material was dispersed in a mixture of Nafion and ethanol. This ink was then applied onto carbon paper using a pipette, with a weight ratio of 9:1 (Ru_xCu_{6-x}/NC_z to binder). Unless specified otherwise, the catalyst mass loading was maintained between 0.1 and 0.2 mg. LCBs were assembled using CR2032 coin cells with perforated cathode casings to facilitate CO_2 diffusion. The anode consisted of 500 µm-thick lithium foil, while the cathode was fabricated following established protocols. A GF/D glass-fiber separator, dried overnight in a vacuum oven at 100°C, was utilized. The electrolyte comprised 1 M LiTFSI dissolved in dimethyl sulfoxide (DMSO), with a volume of 100 µl used for cells with electrolyte configurations. All assembly steps were performed in an argon-filled glovebox, ensuring moisture and oxygen levels remained below 1 ppm. The assembled LCBs were tested in a sealed chamber filled with high-purity CO_2 gas (99.9%). Current densities and specific capacities were calculated based on the mass of the active material (NC_z, Ru_xCu_{6-x}/NC_z or Ru_xCu_{6-x}/KB) deposited on the carbon paper. Battery performance was assessed using a Land Battery Test System and a Neware Battery Test System, with galvanostatic charge/discharge tests conducted within a voltage range of 2.0 to 4.5 V. Cyclic voltammetry (CV) measurements were carried out on a Biologic VMP-3 electrochemical workstation at scan rates of 0.1 or 0.2 mV s^{-1} to identify redox peaks within the designated potential range.

Materials characterizations

X-ray diffraction (XRD) analysis was conducted using a Rigaku MiniFlex 600 diffractometer equipped with Cu Kα radiation. The catalyst and cathode samples were scanned at a rate of 10° min^{-1} to investigate their structural characteristics. Fourier transform infrared (FTIR) spectroscopy was performed on a Nicolet 6700 spectrometer to analyze functional groups. The morphology of the materials and the cathode electrodes, both before and after cycling, was examined using a FEI QUANTA 450 scanning electron microscope. Thermogravimetric analysis (TGA) was carried out using a Mettler Toledo TGA/DSC1 analyzer in an air atmosphere (20 sccm) over a temperature range of 50–800°C at a heating rate of 5 °C min^{-1}. Brunauer-Emmett-Teller (BET) measurements were performed using a Micromeritics TriStar II Plus surface area and porosity analyser for the KB samples, and a Micromeritics 3Flex high-performance gas adsorption analyser for the NC materials. Transmission electron microscopy (TEM) images were obtained with a FEI Tecnai G2 Spirit TEM operated at 120 kV. High-resolution bright-field and high-angle annular dark-field scanning transmission electron microscopy (HAADF-STEM) images, along with energy-dispersive X-ray spectroscopy (EDX) line scans and elemental mapping, were captured using a FEI Titan Themis 80-200 instrument at an acceleration voltage of 200 kV. Synchrotron-based X-ray absorption spectroscopy (XAS) was performed at the 12ID wiggler beamline of the Australian Synchrotron (Melbourne), employing liquid nitrogen-cooled Si (311) monochromator crystals. Measurements at the Ru and Cu K-edge were conducted in beamline mode 3 within Hutch B and the materials are protected in an inert atmosphere prior to testing. The acquired data were processed using the Athena software package, with pure metal references employed for energy calibration and structural refinement. X-ray absorption near-edge structure (XANES) and extended X-ray absorption fine structure (EXAFS) spectra were recorded in fluorescence mode after diluting the samples with cellulose. First-shell EXAFS analysis was performed assuming single-scattering events, and the associated errors were quantified using the R-factor. All ex/in situ XAS measurements were conducted in a high-purity helium atmosphere.

Computational Method

All calculations were performed by using the projector augmented wave method in the framework of density functional theory (DFT), as implemented in the Vienna Ab initio Simulation Package (VASP).[3] The electron exchange-correlation interactions were parameterized by the generalized gradient approximation (GGA) with the Perdew-Burke-Ernzerhof (PBE).[4-5] Spin polarization effect was considered in this work. The Brillouin zone was sampled by the Γ-centered k-mesh with a resolution of 2π × 0.04 $Å^{-1}$. The plane-wave energy cutoff was set to 520 eV, and the convergence tolerance for residual force and energy on each atom during structure relaxation were set to -0.02 eV/Å and 10^{-7} eV, respectively. A vacuum space of 15 Å was applied to the Z axis avoid interactions between the neighboring configurations. Van der Waals interaction (DFT-D3 method with Becke-Jonson damping) was incorporated.[6-7] Structural visualization was conducted utilizing the VESTA software.[8] The VASPKIT code is used to finish the pre- and post-processing of the calculation results.[9]

Figure S1

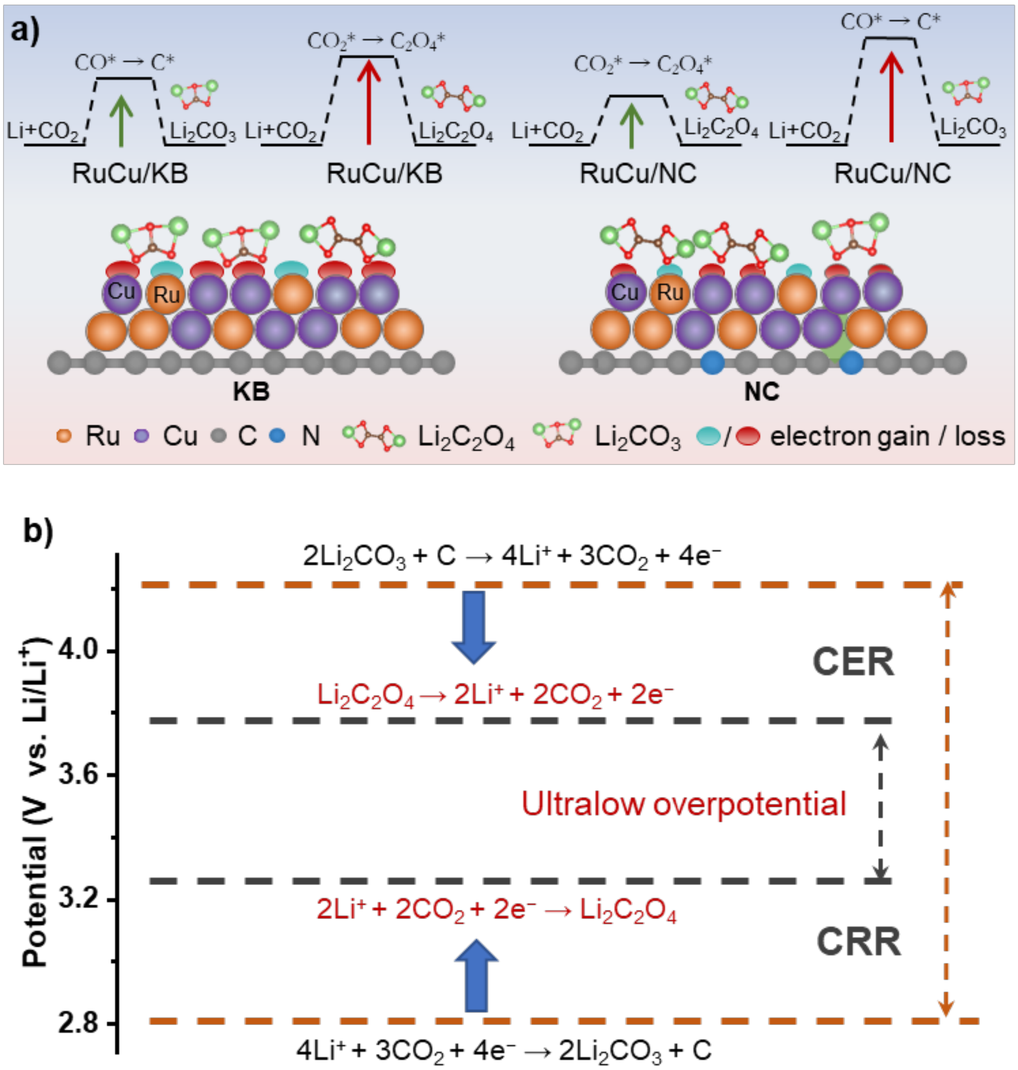

Figure S1. **a**) Schematic of electron modulation in RuCu/KB and RuCu/NC. **b**) Schematic of the synergetic effect of RuCu/NC catalysts for the Li-CO_2 battery system. Discharge voltage of the battery using RuCu/NC catalysts can adjust the output voltage from 2.80 V (CO_2-to-Li_2CO_3 conversion) to above 3.2 V (CO_2-to-Li_2CO_3/$Li_2C_2O_4$ conversion) by RuCu/NC.

Figure S2

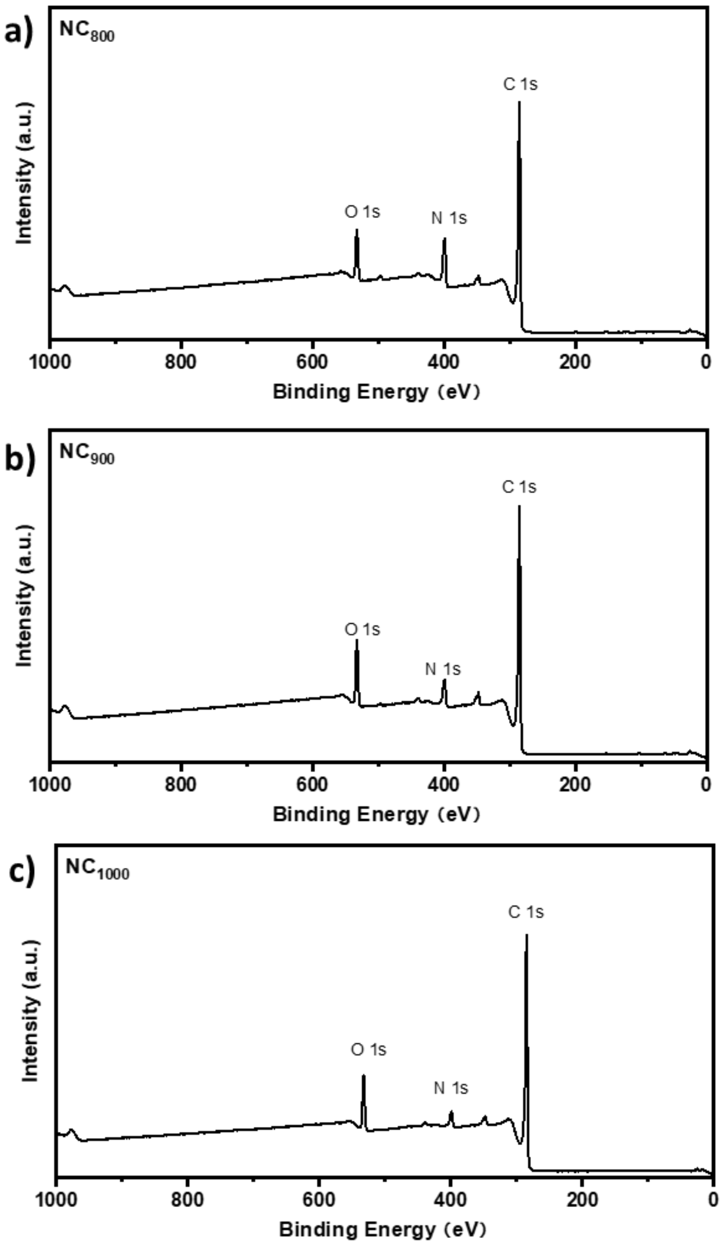

Figure S2. XPS survey of **a**) NC_{800}, **b**) NC_{900}, and **c**) NC_{1000}. The detected element compositions and their weight ratios see Table S2.

Figure S3

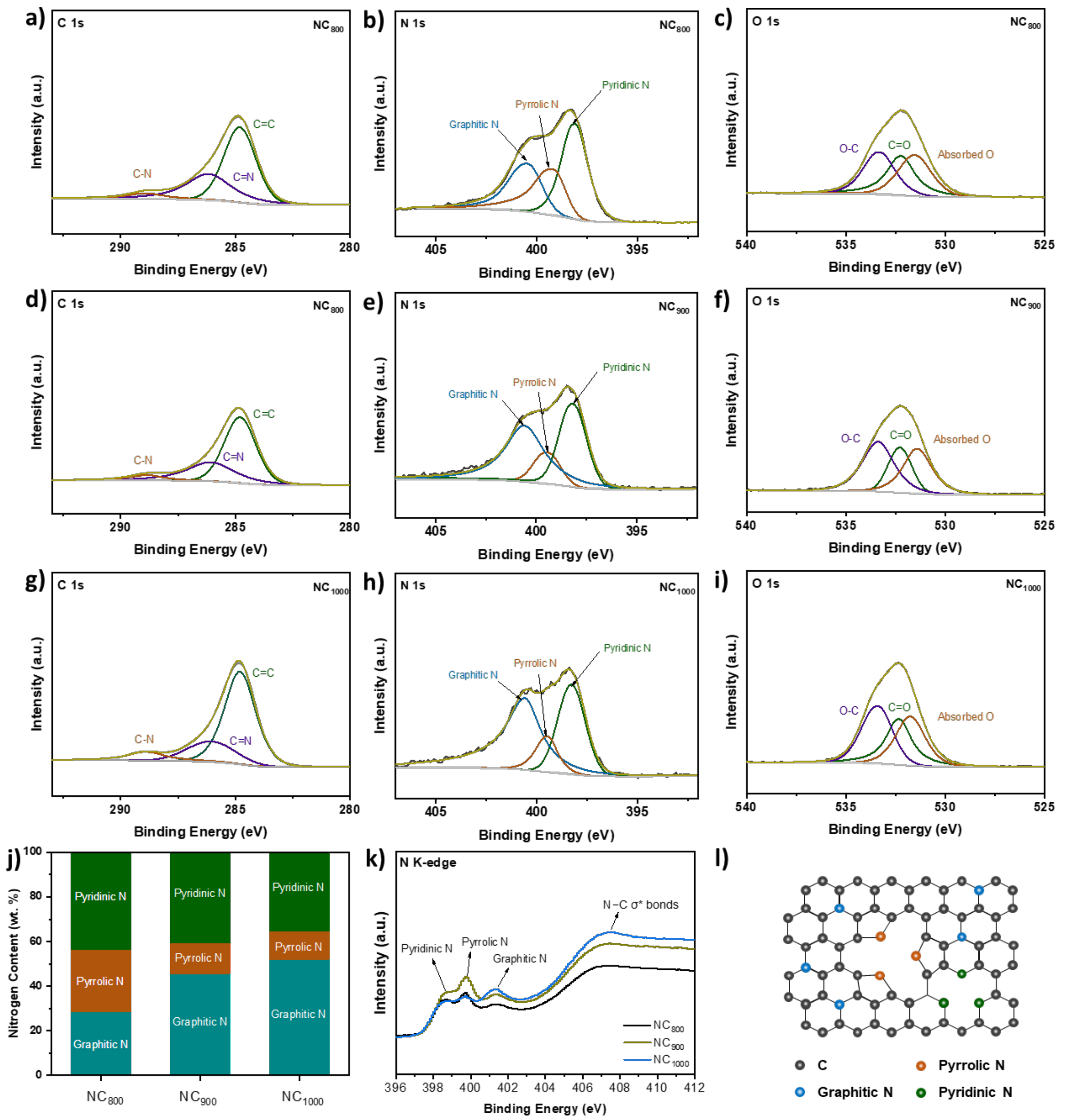

Figure S3. High-resolution XPS spectra. **a, d, g**) C *1s*, **b, e, h**) N *1s*, and **c, f, i**) O *1s* of NC_{800}, NC_{900}, and NC_{1000}. **j**) Summary of the nitrogen content in the NC_{800}, NC_{900}, and NC_{1000}. **k)** The N K-edge XAS spectra of NC_{800}, NC_{900}, and NC_{1000} materials. **l)** Schematic illustration of the structure of different nitrogen species in nitrogen-doped carbon materials.

The peaks in the N *1s* spectra at 398.1 eV, 399.2 eV and 400.2 eV correspond to pyridinic N, pyrrolic N and graphitic N, respectively. NC_{1000} contains the highest proportions of graphitic N (52.1 wt.%), the two nitrogen species most commonly associated with enhanced electrocatalytic activity.[10] Specifically, graphitic N promotes electron donation to the carbon framework, improving the electronic conductivity of the support.[11] The N K-edge obtained from soft XAS spectra (Figure S3k) exhibit four distinct resonances corresponding to pyridine-like, pyrrolic-like, and graphite-like nitrogen species, with an additional peak at ~407 eV attributed to the transition from N *1s* core states to N–C σ* bonds.

Figure S4

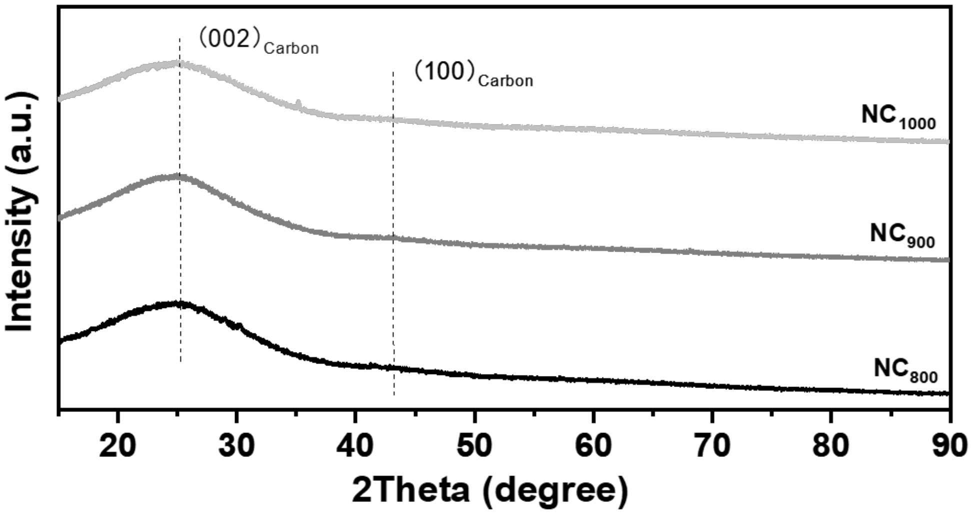

Figure S4. XRD pattern of NC_{800}, NC_{900}, and NC_{1000}.

Figure S5

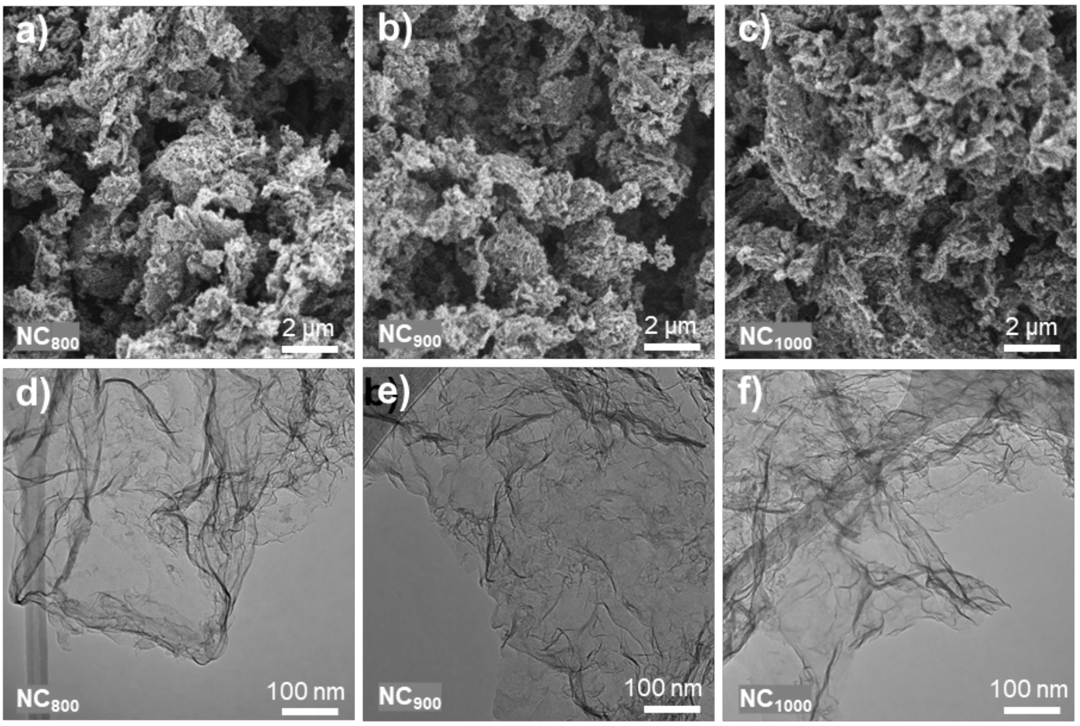

Figure S5. SEM images of **a**) NC_{800}, **b**) NC_{900}, and **c**) NC_{1000}**.** TEM images of **a**) NC_{800}, **b**) NC_{900}, and **c**) NC_{1000}**.**

Figure S6

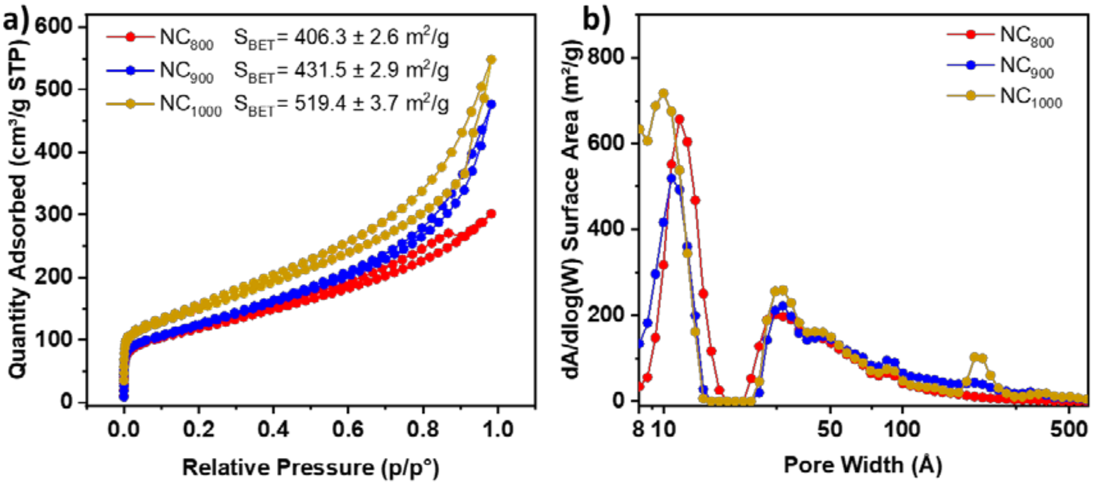

Figure S6. a) Nitrogen adsorption-desorption curves and specific surface areas of NC_{800}, NC_{900}, and NC_{1000}. **b)** BJH Desorption dA/dlog(W) surface area of NC_{800}, NC_{900}, and NC_{1000}.

The surface area and porosity of NC_{800}, NC_{900}, and NC_{1000} were analyzed using the Brunauer-Emmett-Teller (BET) nitrogen adsorption–desorption method. N_2 adsorption–desorption measurements were conducted to assess the impact of calcination temperature on the structural properties. The results indicate that increasing the calcination temperature enhances the specific surface area, which can influence catalytic performance. The BET surface area (S_{BET}) of NC_{800} was measured at 406.3 $m^2\ g^{-1}$, increasing to 519.4 $m^2\ g^{-1}$, for NC_{1000}. The pore size distribution, determined by the Barrett-Joyner-Halenda (BJH) method. The pore sizes of the three materials are mostly micropores and mesopores between 0.8 and 50 nm. Additionally, a slight decrease in pore size with increasing calcination temperature was observed, as supported by pore size distribution analysis (Figure S6a), confirming that most pores reside in the microporous region and that the three materials exhibit similar morphological characteristics (Figure S5), revealed that the change of micropores (~1 nm) significantly contribute to the overall surface area.

Additionally, previous studies indicate that increasing nitrogen content in carbon structures leads to reduced electronic conductivity. Therefore, considering the similar carbon structure and pore size distribution among all synthesised NC matrixes, in this work, NC_{1000} was selected as the most suitable one for Li-CO_2 battery applications.[12-13]

Figure S7

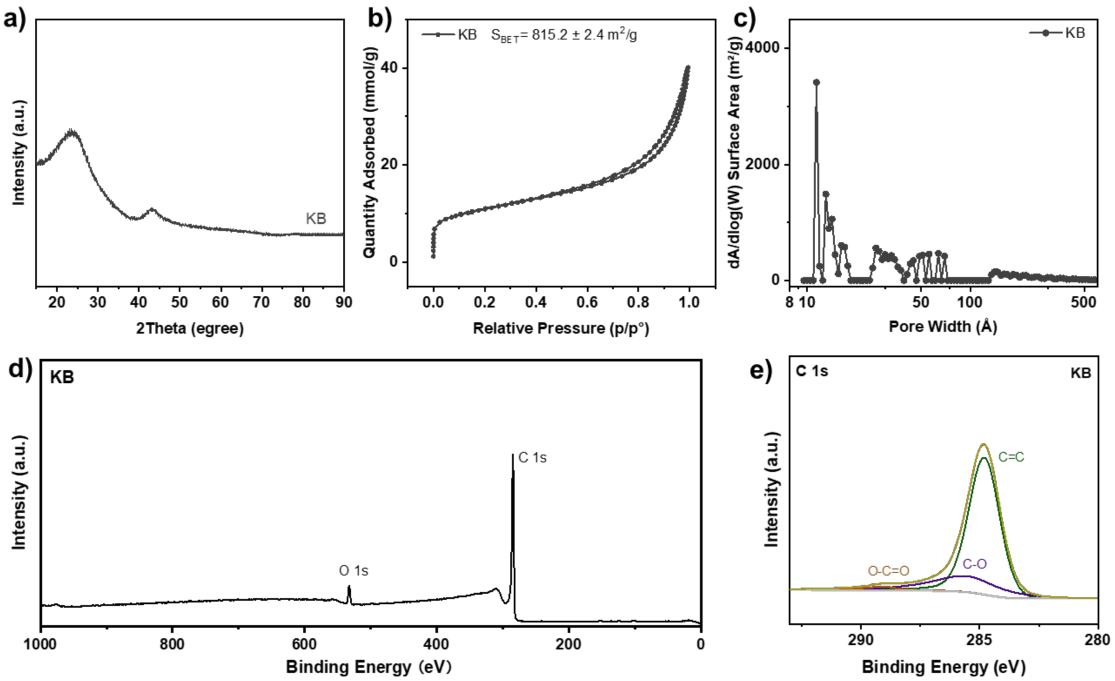

Figure S7. a) XRD pattern of KB. **b)** Nitrogen adsorption-desorption curves and specific surface areas of KB. **c)** BJH Desorption dA/dlog(W) surface area of KB. **d)** XPS survey spectrum of KB. **e)** High-resolution C *1s* XPS spectrum of KB.

Figure S8

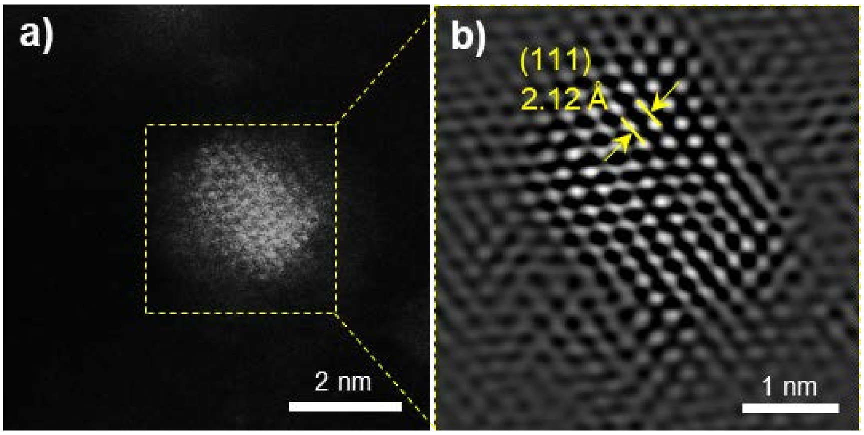

Figure S8. a) Atomically resolved HAADF-STEM image of a Ru_2Cu_4/NC_{1000} nanoparticle. **b)** Simulated crystal structure image of the selected region in a).

Figure S9

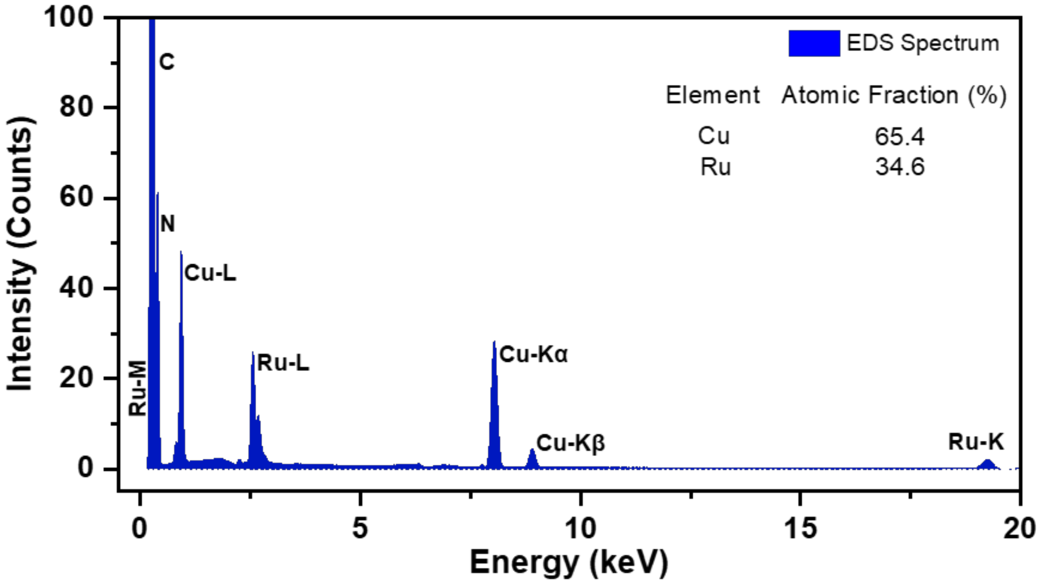

Figure S9. The EDS spectrum of the selected particle in Figure 1e indicates that the Ru_2Cu_4/NC_{1000} composites consist of Ru and Cu elements, which are uniformly distributed throughout the particle, with a Ru: Cu ratio of 1:1.89**.**

Figure S10

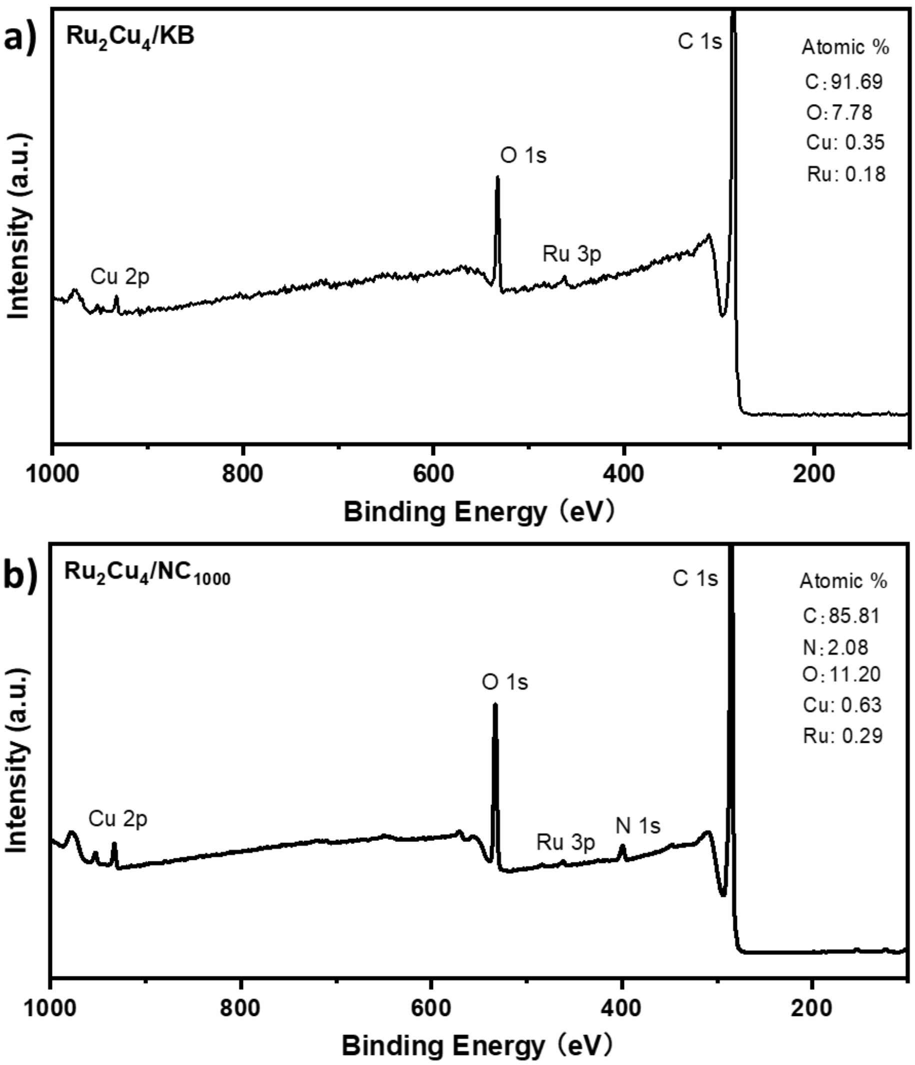

Figure S10. XPS survey of **a**) Ru_2Cu_4/KB and **b**) Ru_2Cu_4/NC_{1000}.

The detected elemental compositions and atomic ratios show that the Ru_2Cu_4/KB and Ru_2Cu_4/NC_{1000} composites contain Ru and Cu with Ru: Cu ratios of 1:1.94 and 1:2.17, respectively. These values are generally consistent with the theoretical feed ratio of 2:1.

Figure S11

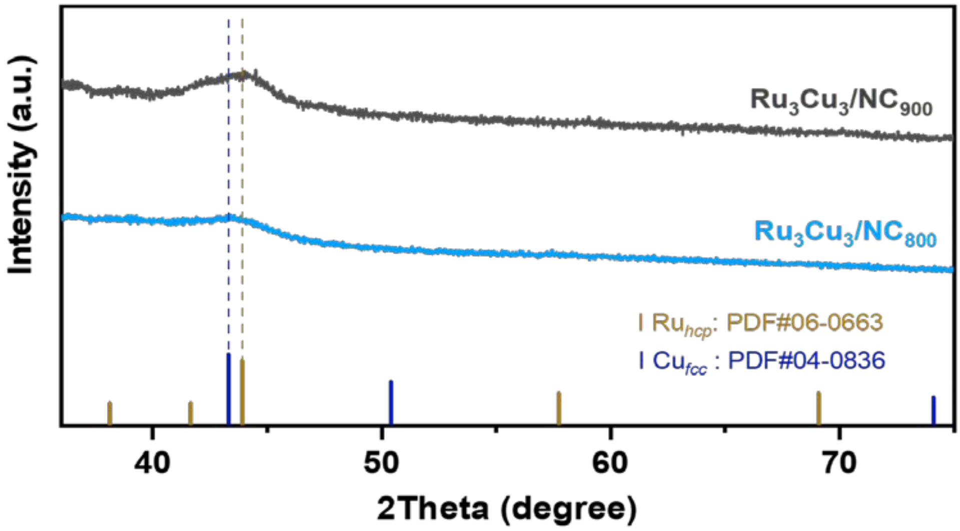

Figure S11. XRD pattern of Ru_3Cu_3/NC_{800} and Ru_3Cu_3/NC_{900}.

To investigate the influence of carbon support on the crystal structure of Ru–Cu alloys, RuCu catalysts with a fixed Ru/Cu atomic ratio of 3:3 were synthesized on NC_{800} and NC_{900} under identical reaction conditions. XRD analysis reveals that RuCu alloys supported on NC_{800} display broad diffraction features characteristic of a Ru–Cu solid solution, indicating uniform alloying and small crystallite sizes. In contrast, when supported on NC_{900}, the diffraction patterns show pronounced phase separation, with a dominant *hexagonal close-packed* (*hcp*) Ru phase accompanied by a *face-centered cubic* (*fcc*) Cu phase. Specifically, the appearance of a strong diffraction peak at ~43.9° is attributed to the *hcp* Ru (011) plane, while a weak shoulder peak at ~43.1° corresponds to *fcc* Cu, evidencing partial segregation of Ru and Cu rather than a homogeneous solid solution. These results demonstrate that the carbon support plays a critical role in stabilizing the alloy crystal phase. Based on its ability to preserve the solid solution structure, together with its good structural durability and favorable compatibility with uniform alloy dispersion, NC_{800} was therefore selected as the optimal support for Ru and Cu atomic ratios studies.

Figure S12

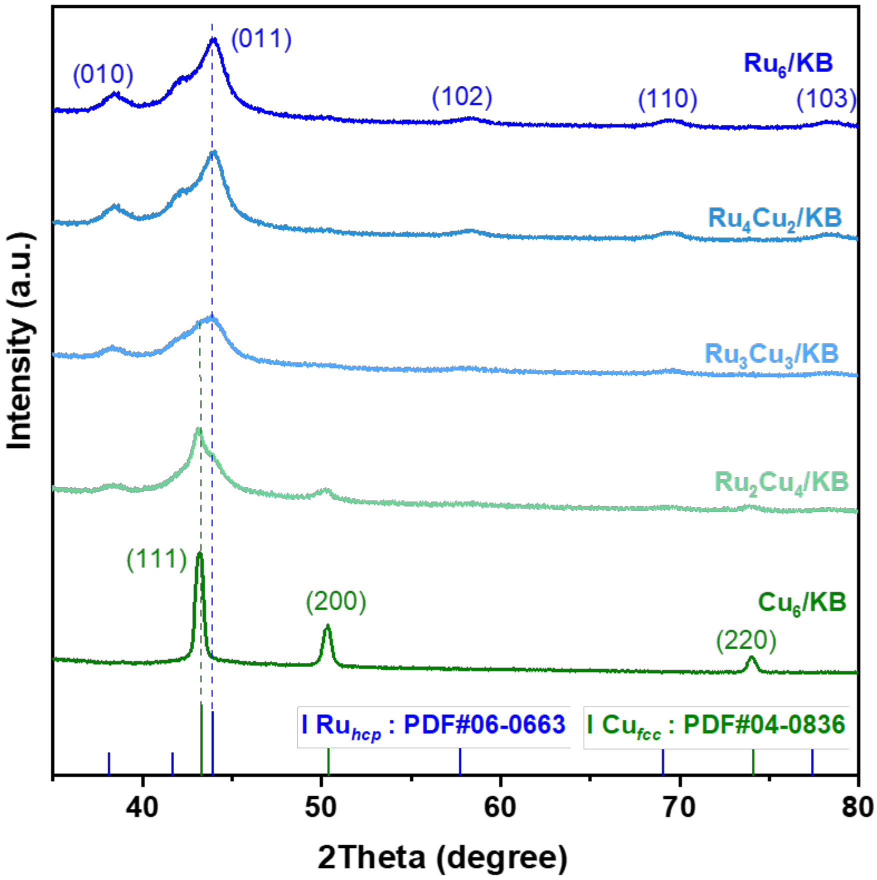

Figure S12. XRD pattern of Cu_6/KB, Ru_2Cu_4/KB, Ru_3Cu_3/KB, Ru_4Cu_2/KB, and Ru_6/KB materials.

RuCu alloy samples supported by KB with varying Ru and Cu atomic ratios (Table S1) were synthesized by adjusting the molar feed ratios of Ru to Cu from 0 to 1, under consistent reaction conditions. XRD analysis confirmed alloys prepared with Cu_6 and Ru_2Cu_4 compositions exhibited the face-centered cubic (*fcc*) structure characteristic of Cu. As the Ru content increased, the alloy structure transitioned to the hexagonal close-packed (*hcp*) phase typical of Ru, as observed in Ru_3Cu_3, Ru_4Cu_2, and Ru_6 compositions.

Figure S13

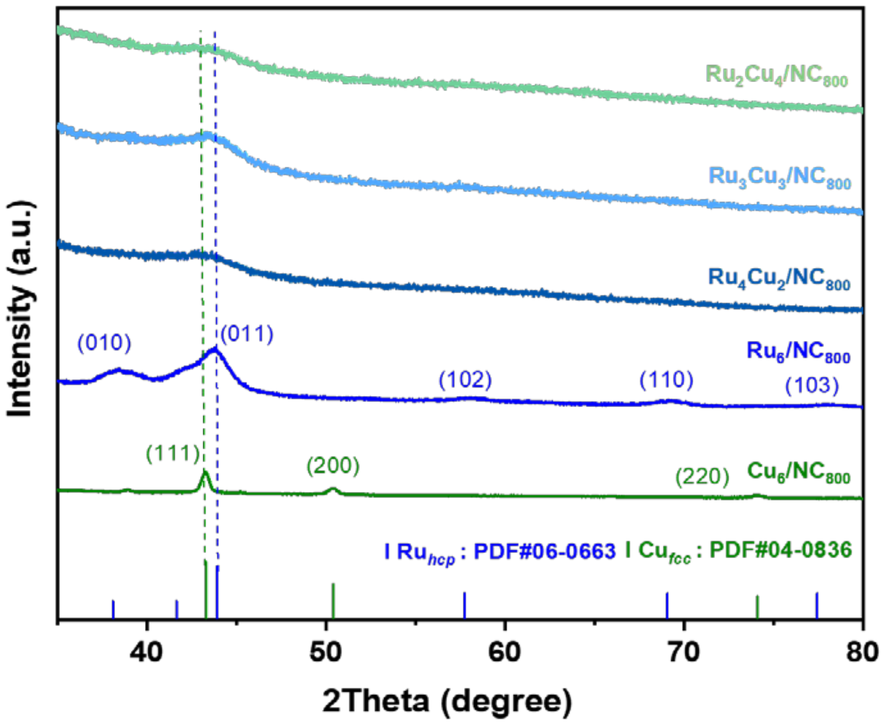

Figure S13. XRD pattern of Cu_6/NC_{800}, Ru_6/NC_{800}, Ru_4Cu_2/NC_{800}, Ru_3Cu_3/NC_{800}, and Ru_2Cu_4/NC_{800} materials.

Figure S14

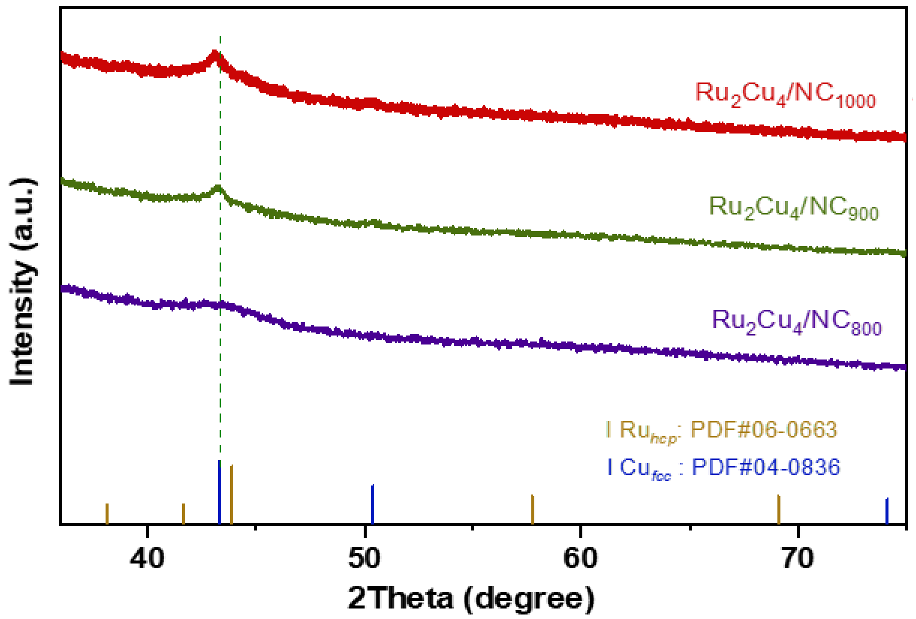

Figure S14. XRD pattern of Ru_2Cu_4/NC_{800}, Ru_2Cu_4/NC_{900}, and Ru_2Cu_4/NC_{1000} materials.

Figure S15

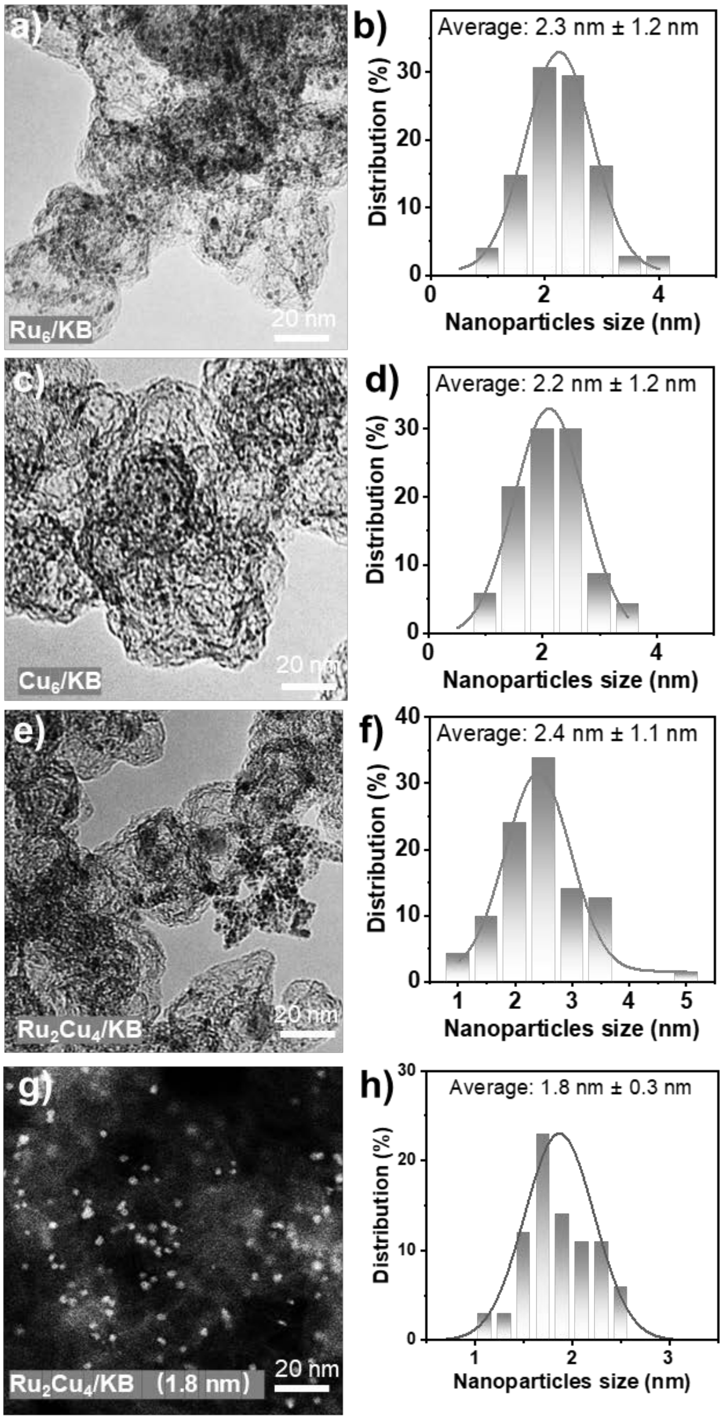

Figure S15. TEM images of **a)** Ru_6/KB, **c)** Cu_6/KB and **e)** Ru_2Cu_4/KB. Size distribution histogram of **b)** Ru_6/K, **d)** Cu_6/KB and **f)** Ru_2Cu_4/KB. **g)** STEM images and **h**) size distribution histogram (counted number of particles: 100) of Ru_2Cu_4/KB (in 1.8 nm).

Figure S16

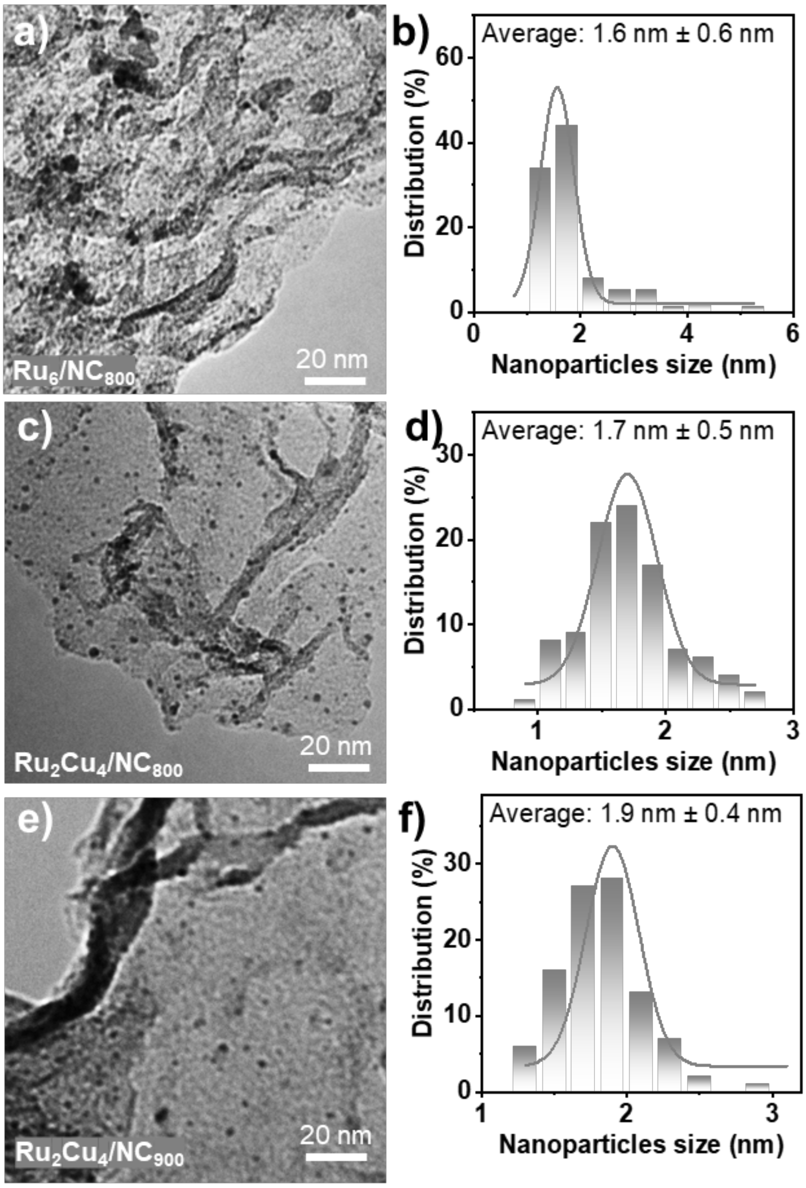

Figure S16. TEM images of **a)** Ru_6/NC_{800}, **c)** Ru_2Cu_4/NC_{800}, and **e)** Ru_2Cu_4/NC_{900}. Size distribution histogram (counted number of particles: 100) of **b)** Ru_6/NC_{800}, **d)** Ru_2Cu_4/NC_{800}, and **f)** Ru_2Cu_4/NC_{900}.

Figure S17

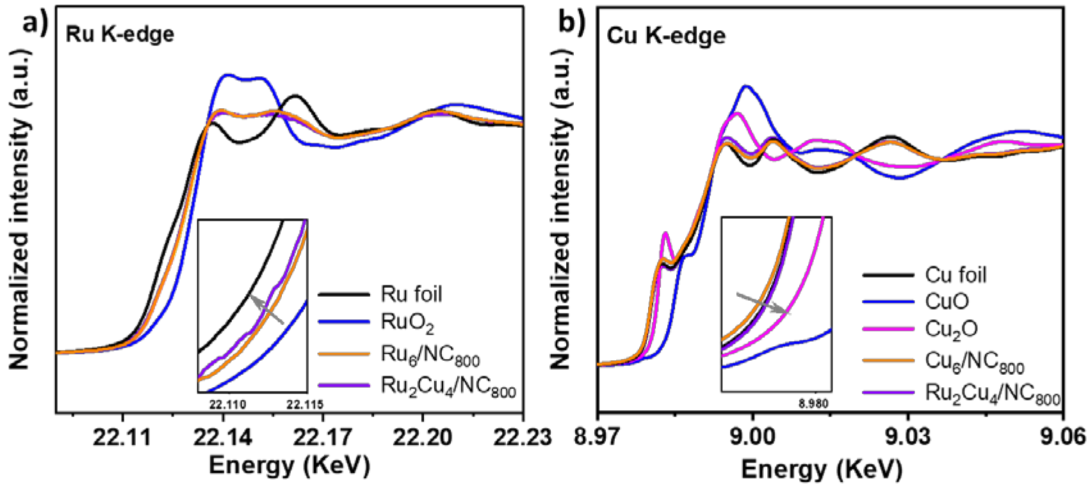

Figure S17. X-ray absorption near edge structure (XANES) spectra of the **a**) Ru K-edge in Ru foil, RuO_2, Ru_6/NC_{800}, and Ru_2Cu_4/NC_{800} materials and **b**) Cu K-edge in Cu foil, CuO, Cu_2O, Cu_6/NC_{800}, and Ru_2Cu_4/NC_{800} materials.

In Figure S17a, the observed white lines of Ru are close to metallic valence state, which are different from corresponding oxide reference samples. This finding suggests that Ru in the Ru_6/NC_{800} and Ru_2Cu_4/NC_{800} are predominantly in metallic states, which is also confirmed by the first derivative of the Ru K-edge XANES spectrum (Figure S22). Compared with the white line of Ru_6/NC_{800}, Ru_2Cu_4/NC_{800} is closer to Ru foil, which means with the adding of Cu, resulting in electron enrichment of Ru (Figure S17a, insert). The absorption edges of the Cu_6/NC_{800} and Ru_2Cu_4/NC_{800} are close to Cu foil (Figure S17b, insert), suggesting that the copper species predominantly exhibit a metal state. Compared to the white line of Cu_6/NC_{800}, Ru_2Cu_4/NC_{800} exhibits a shift to higher energy, indicating electron loss from Cu. Combined with the analysis in Figure S17a, this suggests that electrons are transferred from Cu to Ru.

Figure S18

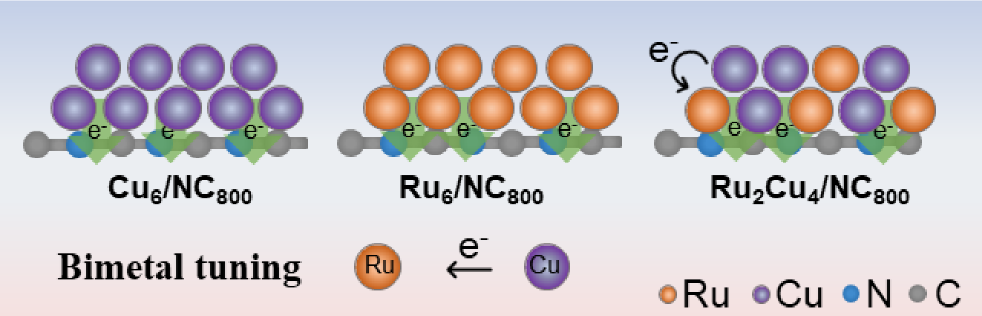

Figure S18. Schematic of electron modulation in RuCu/NC through bimetallic tuning.

Figure S19

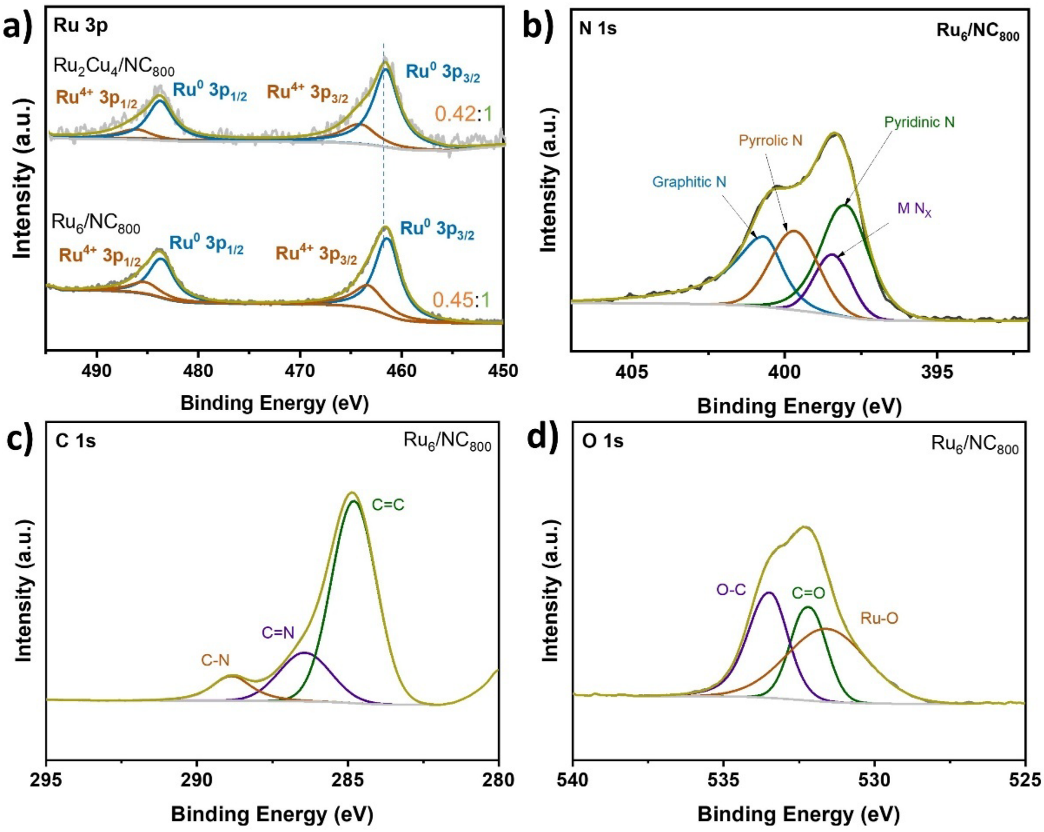

Figure S19. High-resolution XPS spectra. **a**) Ru *2p*, **b**) N *1s*, **c**) C *1s*, and **d**) O *1s* of Ru_6/NC_{800} and Ru_2Cu_4/NC_{800}.

The Ru $2p_{3/2}$ XPS fitting curves revealed that Ru was mainly 0 mix with +4 valence in Ru_6/NC_{800} and Ru_2Cu_4/NC_{800}, which also confirmed by Figure S17 and S22. The Ru^{4+}: Ru^0 ratio, which decreases from 0.45:1 in Ru_6/NC_{800} to 0.42:1 in Ru_2Cu_4/NC_{800} (Figure S19a), illustrate the Ru^0 increase in Ru_2Cu_4/NC_{800}. Compared to Ru_6/NC_{800}, the increase in Ru^0 characteristic peak means the Ru metal atoms in the NC-supported Ru_2Cu_4 alloy as the electron acceptors, resulting in electron-rich Ru atom in the Ru_2Cu_4/NC_{800} samples. This also illustrates that with the adding of Cu, the electrons transfer from Cu to Ru.

Figure S20

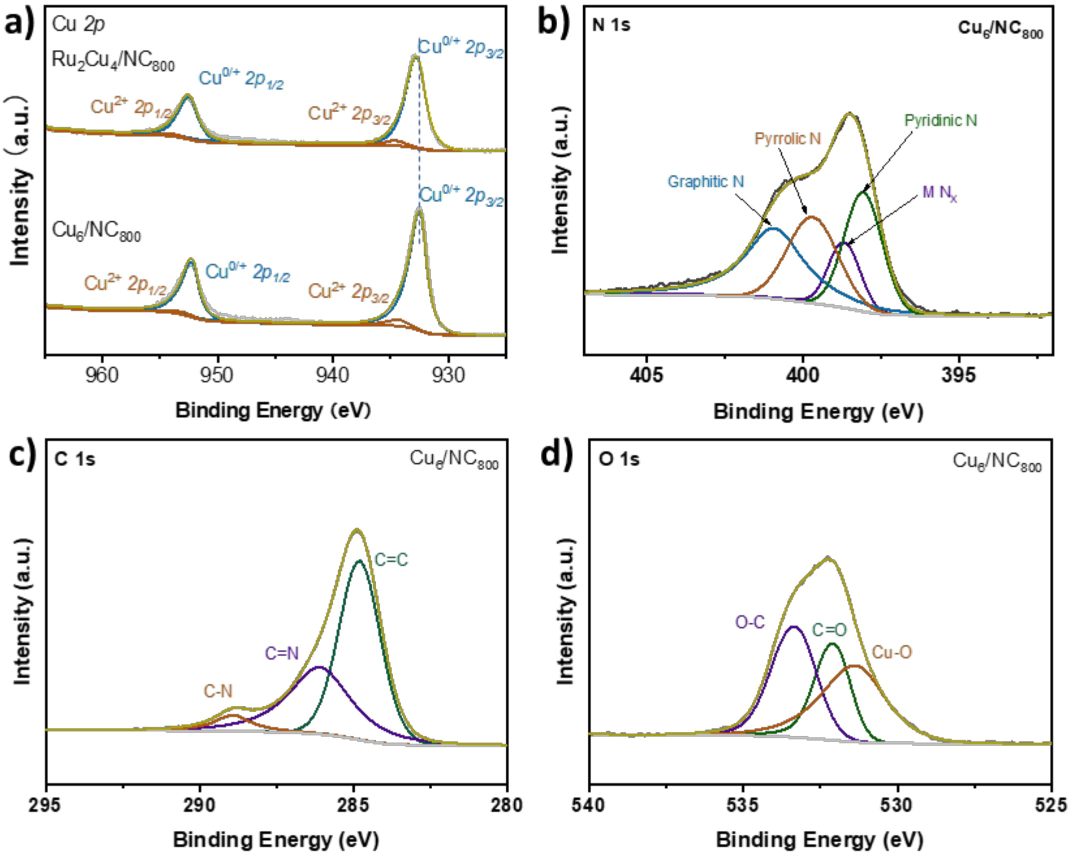

Figure S20. High-resolution XPS spectra. **a**) Cu *2p*, **b**) N *1s*, **c**) C *1s*, and **d**) O *1s* of Cu_6/NC_{800} and Ru_2Cu_4/NC_{800}.

The Cu $2p_{3/2}$ XPS fitting curves revealed that Cu was mainly 0/+1 valence with +2 valence in Cu_6/NC_{800} and Ru_2Cu_4/NC_{800}. Compared to Cu_6/NC_{800}, a positive shift of Cu characteristic peaks means Cu metal atoms in the NC-supported Ru_2Cu_4 alloy as the electron donators, giving an electron-deficient Cu atom in the Ru_2Cu_4/NC_{800} samples. This also illustrates that with the adding of Ru, the electrons transfer from Cu to Ru.

Figure S21

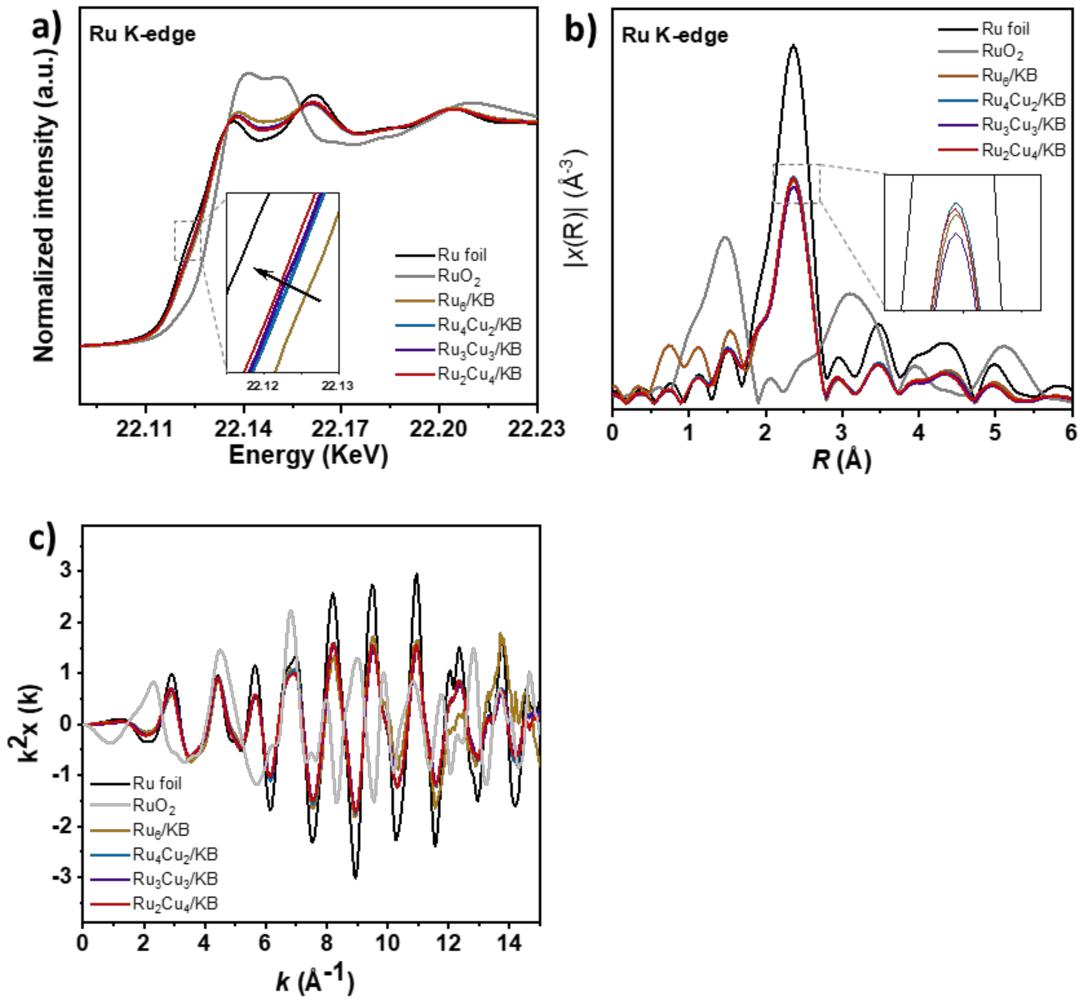

Figure S21. **a**) XANES spectra of Ru in Ru_xCu_{6-x}/KB materials. **b**) EXAFS spectra for Ru K-edge. **c**) Experimental k^2-weighted EXAFS oscillations at Ru K-edge.

To further investigate the influence of N-doping on CRR/CER activity, XANES and FT-EXAFS data of Ru K-edge for Ru_xCu_{6-x}/KB are shown in Figure S21. The XANES spectra of Ru_xCu_y/KB suggest that the Ru ions were reduced to metallic Ru. The FT-EXAFS data also revealed a peak for Ru-Ru in R space, indicating that Ru in Ru_xCu_{6-x}/KB agglomerated into larger metallic particles. The white line of Ru progressively shifted to lower values in Figure S21a, indicating gradual alloying and strong electronic interactions between Ru and Cu.

Figure S22

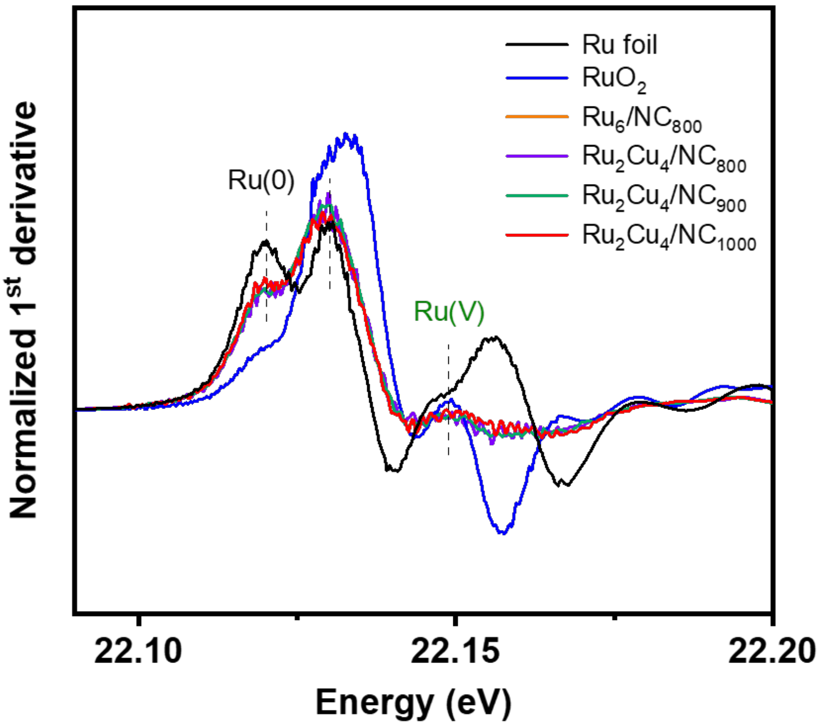

Figure S22. First-order derivatives Ru in Ru foil, RuO_2, Ru_6/NC_{800}, Ru_2Cu_4/NC_{800}, Ru_2Cu_4/NC_{900}, and Ru_2Cu_4/NC_{1000} materials.

Figure S23

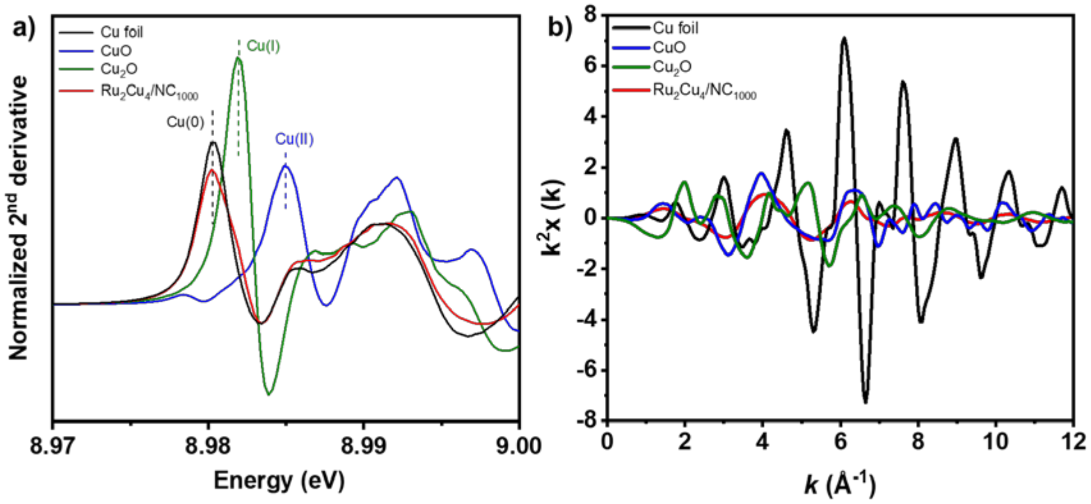

Figure S23. a) First-order derivatives of the XANES spectra of Cu in in Cu foil, CuO, Cu_2O, and Ru_2Cu_4/NC_{1000} materials. To allow for an accurate analysis of the oxidation state of Cu, we use the first-order derivatives of the copper XANES spectra, which is generally accepted by previous publications.[14] **b)** Experimental k^2-weighted EXAFS oscillations at Cu K-edge.

Figure S24

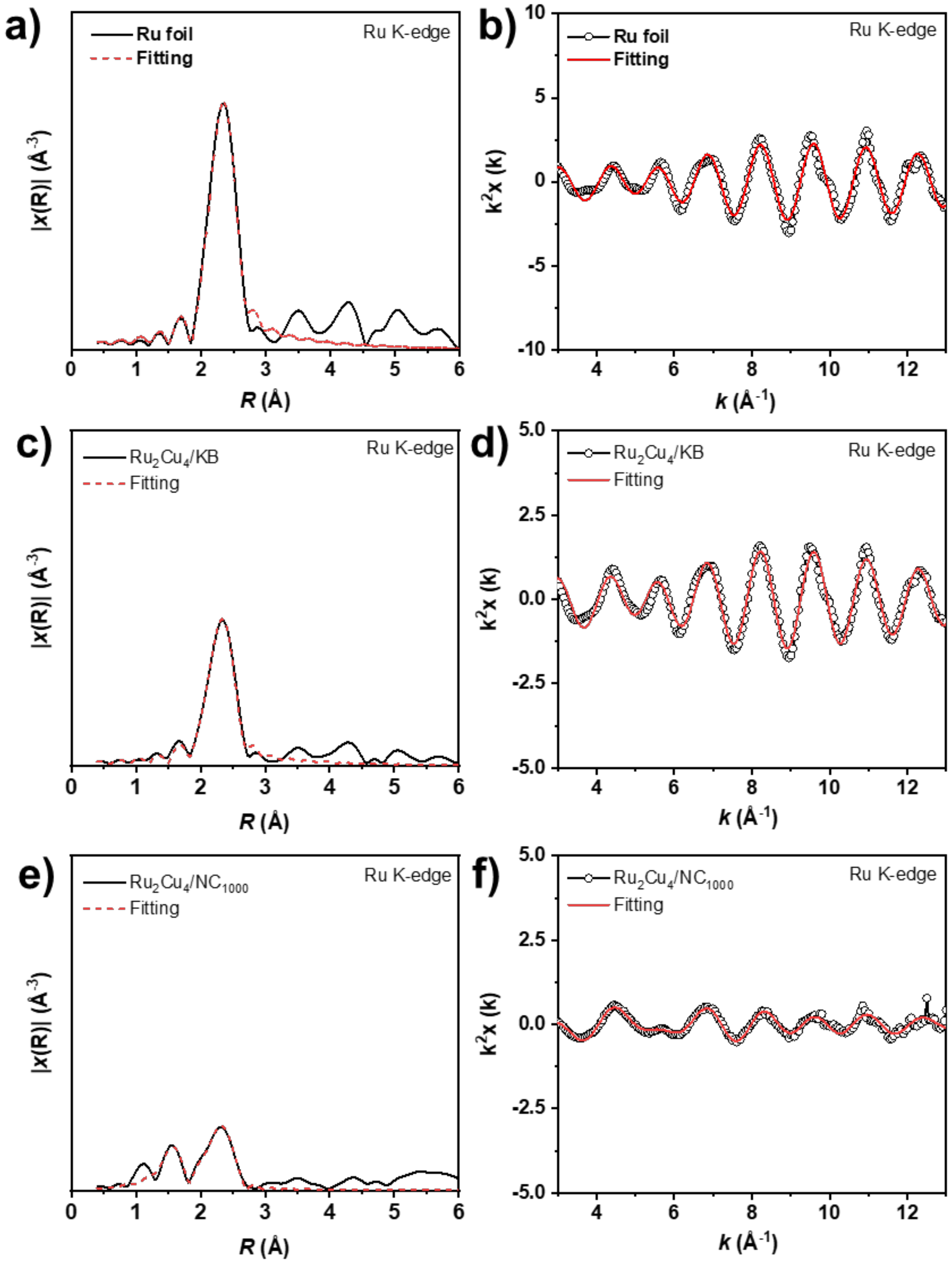

Figure S24. a-f) Fitted EXAFS data in R and K space of Ru K-edge. The detailed fitting information can be found in Table S3.

Figure S25

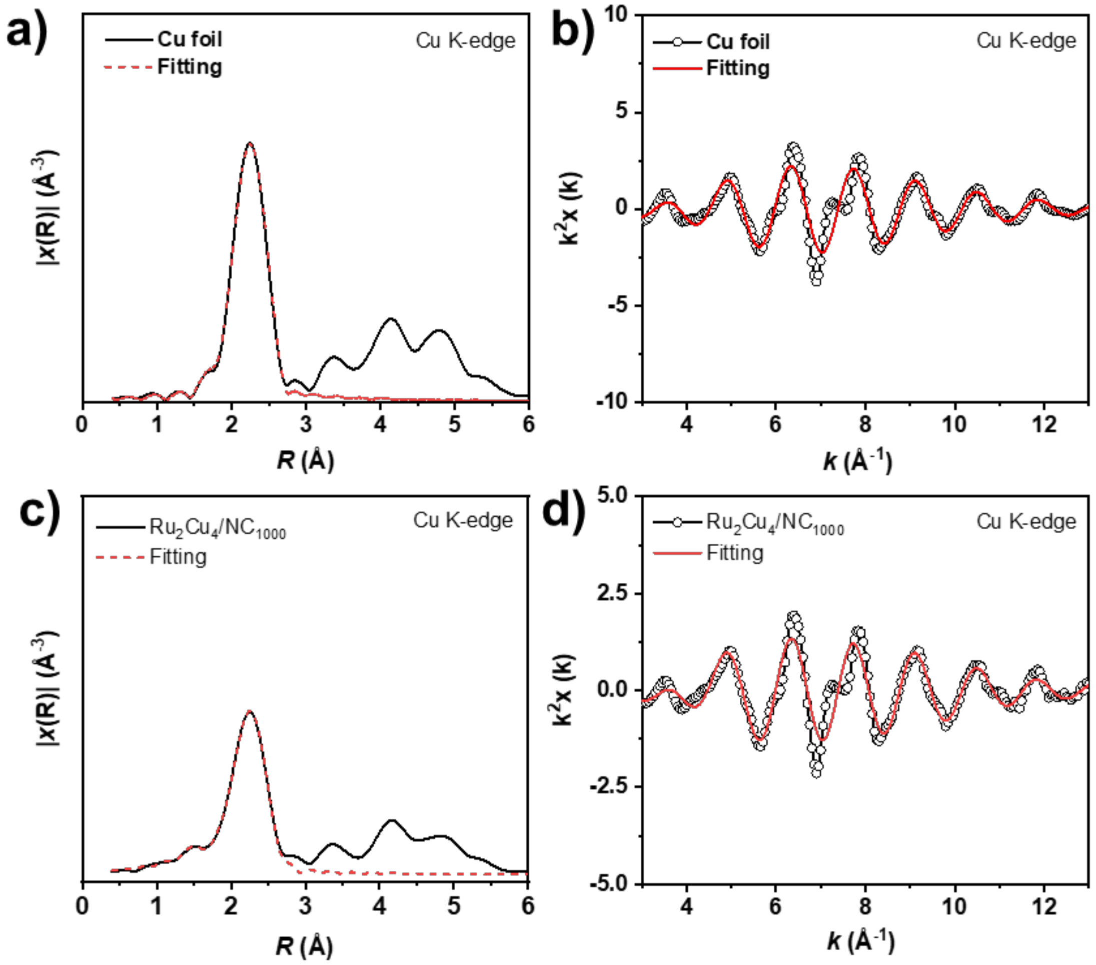

Figure S25. a-d) Fitted EXAFS data in R and K space of Cu K-edge. The detailed fitting information can be found in Table S3.

Figure S26

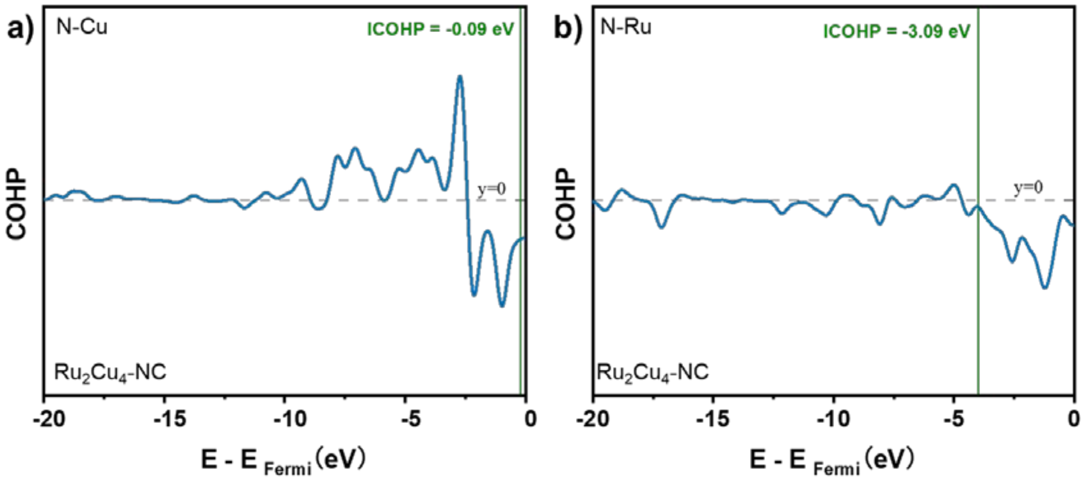

Figure S26. Crystal Orbital Hamilton Population (COHP) of **a**) N-Cu and **b**) N-Ru in Ru_2Cu_4-NC model.

Crystal orbital Hamilton population (COHP) analysis was employed to elucidate the energy-resolved atomic interactions. As shown in Figure S26, the Ru--N interaction in Ru_2Cu_4-NC exhibits pronounced bonding states predominantly distributed below the Fermi level, corresponding to occupied bonding orbitals. Moreover, the integrated COHP (ICOHP) value for the Ru-N bond in Ru_2Cu_4-NC reaches −3.09 eV, which is significantly more negative than that of Cu-N bond in Ru_2Cu_4-NC (−0.09 eV). These results indicate a much stronger Ru-N interaction in the N-doped system. In contrast, Cu–N bonding is considerably weaker, suggesting that nitrogen preferentially coordinates with Ru atoms. Such strong Ru-N coupling plays a dominant role in modulating the local electronic structure of Ru_2Cu_4-NC, thereby influencing the overall electronic properties of the catalyst.

Figure S27

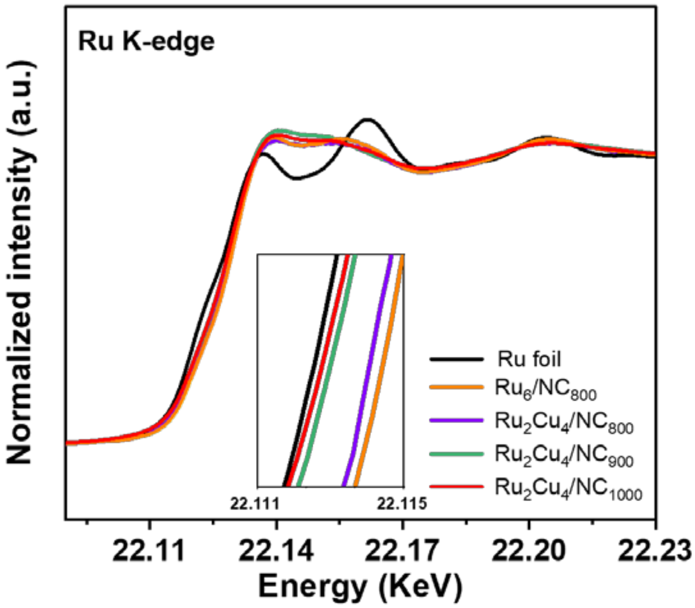

Figure S27. X-ray absorption near edge structure (XANES) spectra of the Ru K-edge in Ru_2Cu_4/NC_{800}, Ru_2Cu_4/NC_{900}, and Ru_2Cu_4/NC_{1000} materials.

Compare with Ru_2Cu_4/NC_{800}, the Ru K-edge absorption threshold position in the Ru_2Cu_4/NC_{900} and Ru_2Cu_4/NC_{1000} samples slightly shifted to a negative value with the N decrease (Figure S27a, insert), accompanied by a reduced intensity of the white line, which indicates an electron density transfer from neighbouring atoms to Ru and a change in the coordination environmental of Ru species.

Figure S28

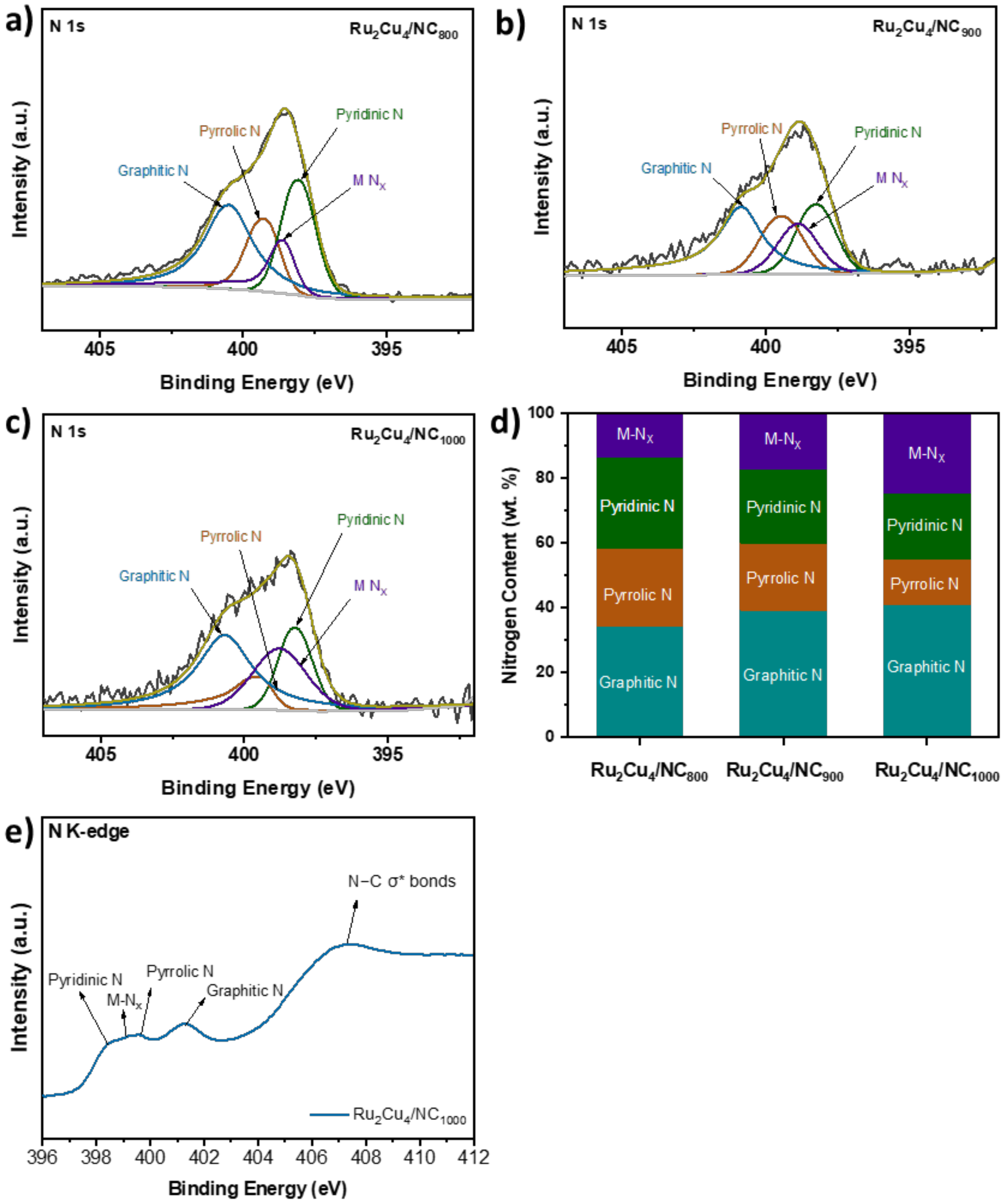

Figure S28. High-resolution XPS spectra. **a-c**) N *1s* of Ru_2Cu_4/NC_{800}, Ru_2Cu_4/NC_{900}, and Ru_2Cu_4/NC_{1000}. **d**) Summary of the nitrogen content in the carbon samples. **e)** The N K-edge XAS spectra of Ru_2Cu_4/NC_{1000} material.

Figure 29

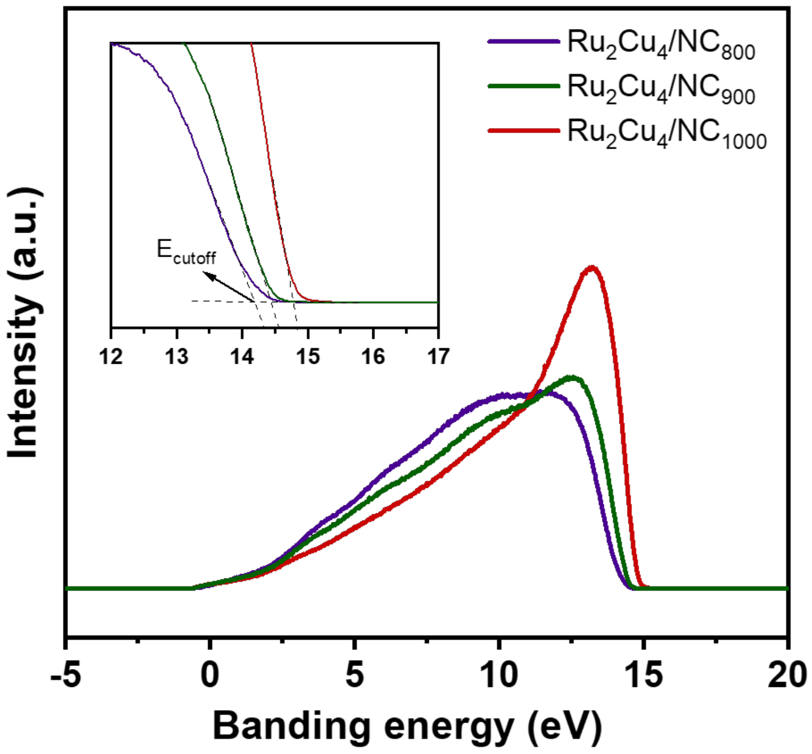

Figure S29. The local magnification of ultraviolet photo-electron spectroscopy (UPS) of Ru_2Cu_4/NC_{800} (6.40 eV), Ru_2Cu_4/NC_{900} (6.14 eV), and Ru_2Cu_4/NC_{1000} (5.80 eV) materials.

UPS spectra were recorded after Ar^+ sputtering at 2 kV for 45 s to minimize surface contamination. A He discharge lamp (He I, hν = 21.22 eV) was used for UPS measurements, with a sample bias of -10 eV. The Au $4f_{7/2}$ core-level signal at a binding energy of 83.8 eV served as the reference for electron energy calibration. The work function was determined using the equation ϕ = hν – (E_{cutoff} – $E_{Fermi\ level}$), where hν corresponds to the UV photon energy (21.22 eV), E_{cutoff} indicates the final state energy, and $E_{Fermi\ level}$ represents the initial state energy.[15]

As shown in Figure S29, the work functions of Ru_2Cu_4/NC_{800}, Ru_2Cu_4/NC_{900}, and Ru_2Cu_4/NC_{1000} were determined to be 6.40 eV, 6.14 eV, and 5.80 eV, respectively, indicating the relatively lower work function of Ru_2Cu_4/NC_{1000}. The increase in work function with higher nitrogen content aligns with previous studies, where pyridinic nitrogen increases the work function due to reverse electron transfer at the C-N bond.[16-17]

UPS measurements were conducted to examine variations in the work function and electron transfer within the NC-interfaced RuCu heterostructure. The reduced work function of Ru_2Cu_4/NC_{1000} could lower the potential of zero charge, thereby facilitating the CRR by reducing the interfacial CO_2 reorganization barrier.[15] A lower work function reduces the energetic barrier for catalysts to transfer surface electrons to adsorbed CO_2, facilitating intermediate formation, which is the rate-determining step in CRR. This work function modulation strategy offers valuable insight into designing efficient electrocatalysts for enhanced CRR performance.[18]

Figure S30

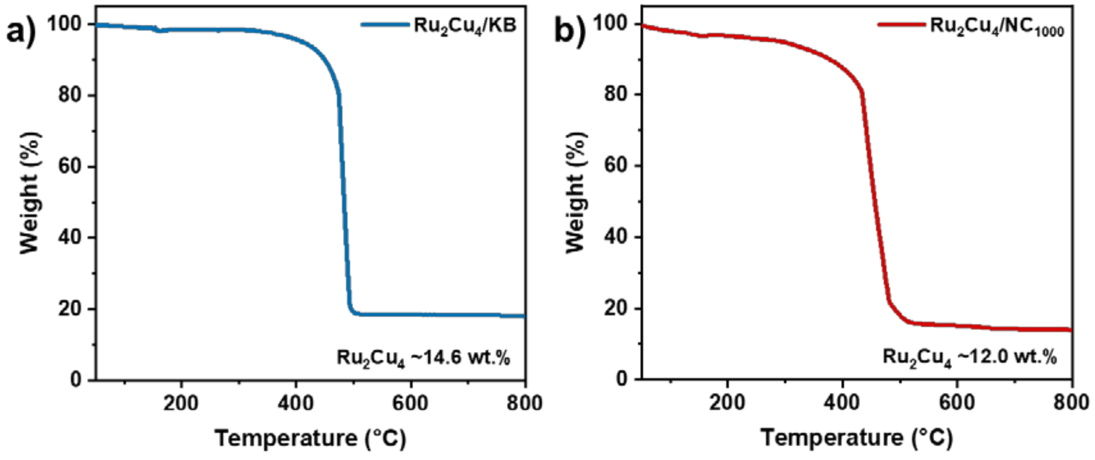

Figure S30. TGA analysis of the **a)** Ru_2Cu_4/KB and **b)** Ru_2Cu_4/NC_{1000}.

Assuming complete combustion of carbon and the conversion reaction of M to metal oxides (MO: Ru_2O or CuO), the content of M in the M/KB catalysts can be determined using the following equation (1).

$$m_M\ (wt.\%) = \frac{m_M}{m_{MO}} \times \frac{Final\ weight}{Initial\ weight} \times 100\% \qquad \text{(Equ.1)}$$

Figure S31

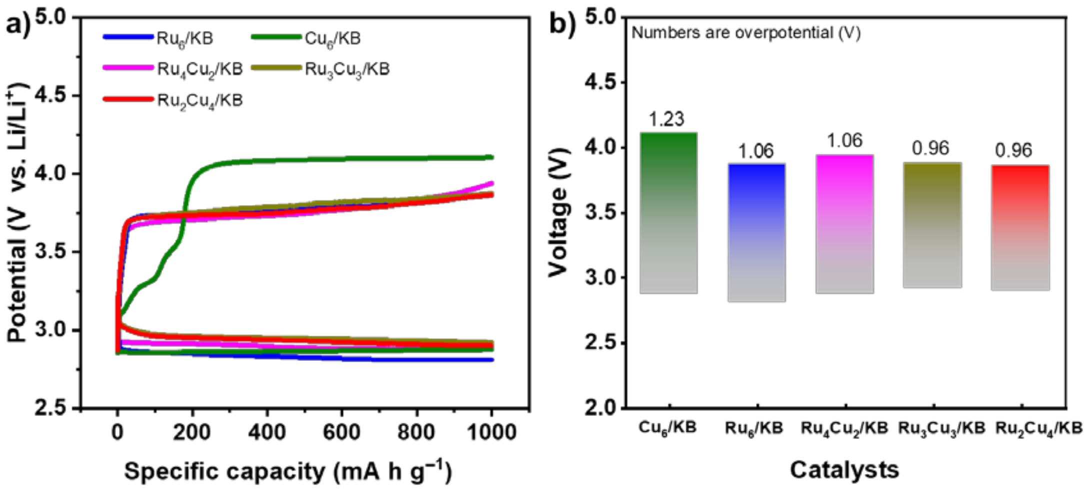

Figure S31. a) Charge and discharge curves Ru_6/KB, Cu_6/KB, Ru_4Cu_2/KB, Ru_3Cu_3/KB, and Ru_2Cu_4/KB materials at current densities of 100 mA g^{-1}. **b)** Battery overpotentials at current densities of 100 mA g^{-1}.

RuCu alloy catalysts supported on KB (Ru_xCu_{6-x}/KB) with varying atomic ratios of Ru and Cu were synthesized and evaluated for their electrochemical performance in LCBs. Although all KB-supported catalysts exhibit discharge plateaus below 3.0 V, the design of multicomponent heterostructures emerges as a promising strategy to enhance electrocatalytic performance, and catalytic engagement. Compared to Ru_6/KB, the RuCu alloy catalysts significantly reduce the Ru content while maintaining, and even improving, catalytic activity. Notably, the Ru_3Cu_3/KB and Ru_2Cu_4/KB catalysts decrease the overpotential from 1.06 V of Ru_6/KB to 0.96 V, demonstrating its superior performance.

Figure S32

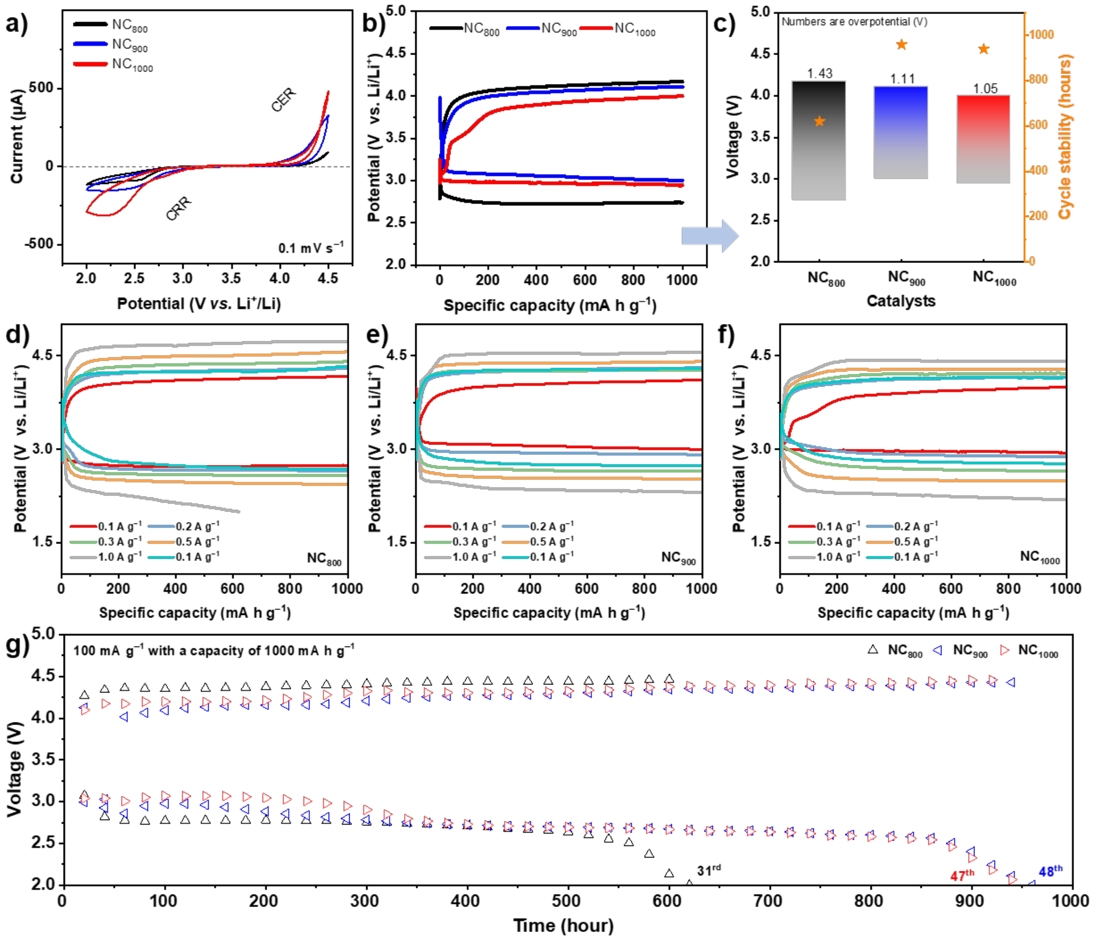

Figure S32. a) CV curves at 0.1 mV s^{-1}. **b)** Charge and discharge curves of NC_{800}, NC_{900}, and NC_{1000} materials at current densities of 100 mA g^{-1}. **c)** Battery overpotentials at current densities of 100 mA g^{-1}. **d-f)** Charge and discharge curves of NC_{800}, NC_{900}, and NC_{1000} materials materials taken at different current densities. **g)** The long cyclic performance of catalysts at 100 mA g^{-1} curtailed the specific capacity of 1000 mAh g^{-1}.

As the nitrogen concentration decreases, the overpotential of NC decreases from 1.43 V in NC_{800} to 1.05 V in NC_{1000}, with the cycle performance increasing to approximately 950 hours. The BET surface area (S_{BET}, Figure S6) increases from 406.3 m^2 g^{-1} for NC_{800} to 519.4 m^2 g^{-1} for NC_{1000}, which appears to contribute to the improved performance of NC_{1000}.

In the XPS peaks (Figure S3) in the N 1s spectra at 398.1 eV, 399.2 eV and 400.2 eV correspond to pyridinic N, pyrrolic N and graphitic N, respectively. NC_{1000} contains a proportion of graphitic N (52.1 wt.%), pyridinic N (35.4 wt.%), pyrrolic N (12.5 wt.%). Previous works indicate that the pyridinic N in N doped carbon as a more favorable site for the CO_2 adsorption, *COOH formation, and *CO removal in proton-based CO_2 reduction.[19] Therefore, it is reasonable to believe that the highly electronegative pyrrolic and pyridinic nitrogen sites act as active sites to rapidly capture CO_2 molecules, while graphitic N sites facilitate fast electron transfer, both of which drive the exceptional reaction kinetics and yields the initial discharge plateau near 3.0 V.

Figure S33

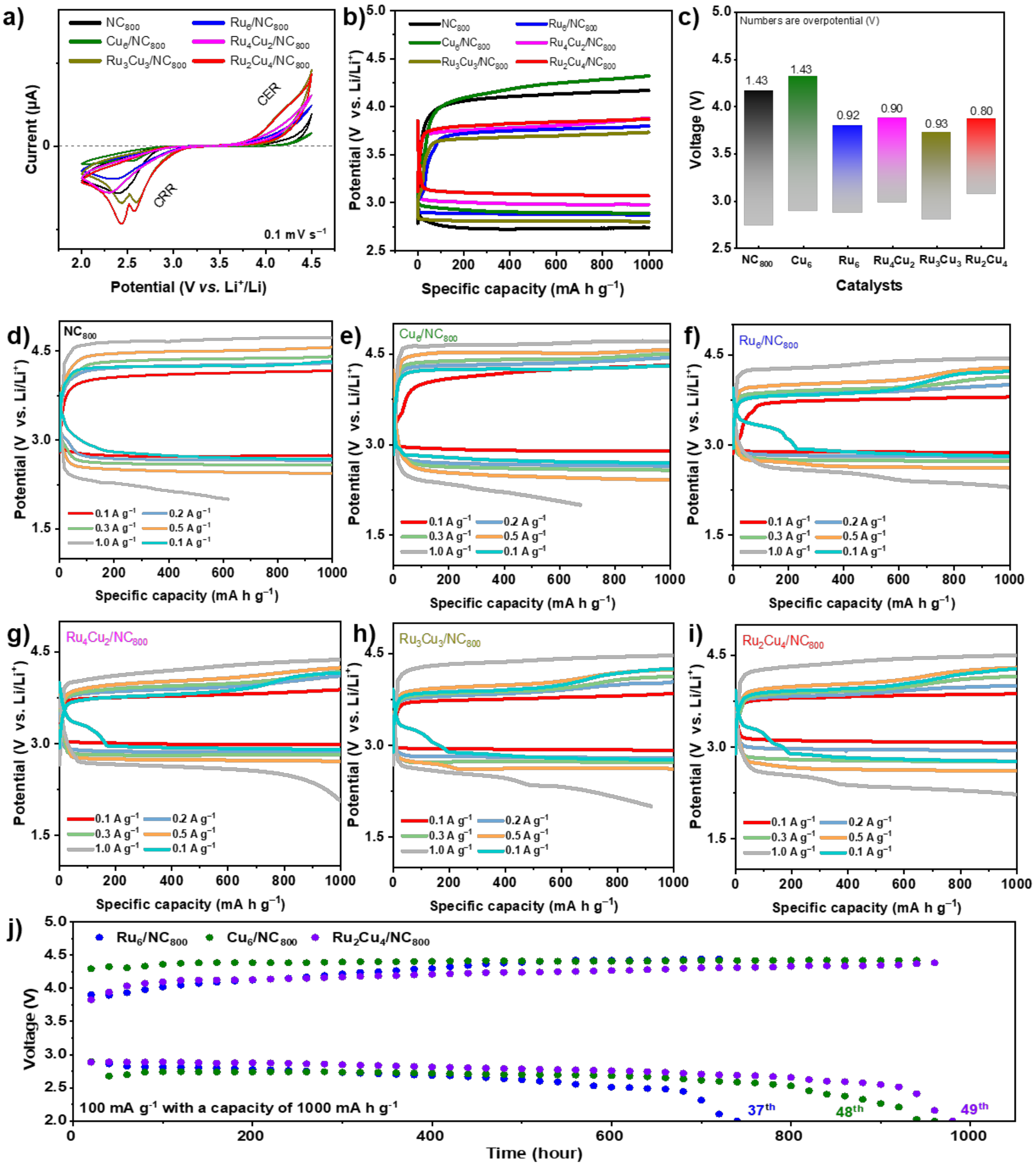

Figure S33. a) CV curves at 0.1 mV s^{-1}. **b)** Charge and discharge curves NC_{800}, Cu_6/NC_{800}, Ru_6/NC_{800}, Ru_4Cu_2/NC_{800}, Ru_3Cu_3/NC_{800}, and Ru_2Cu_4/NC_{800} materials at current densities of 100 mA g^{-1}. **c)** Battery overpotentials at current densities of 100 mA g^{-1}. **d-i)** Charge and discharge curves of NC_{800}, Cu_6/NC_{800}, Ru_6/NC_{800}, Ru_4Cu_2/NC_{800}, Ru_3Cu_3/NC_{800}, and Ru_2Cu_4/NC_{800} materials taken at different current densities. **j)** The long cyclic performance of catalysts at 100 mA g^{-1} curtailed the specific capacity of 1000 mAh g^{-1}.

Figure S34

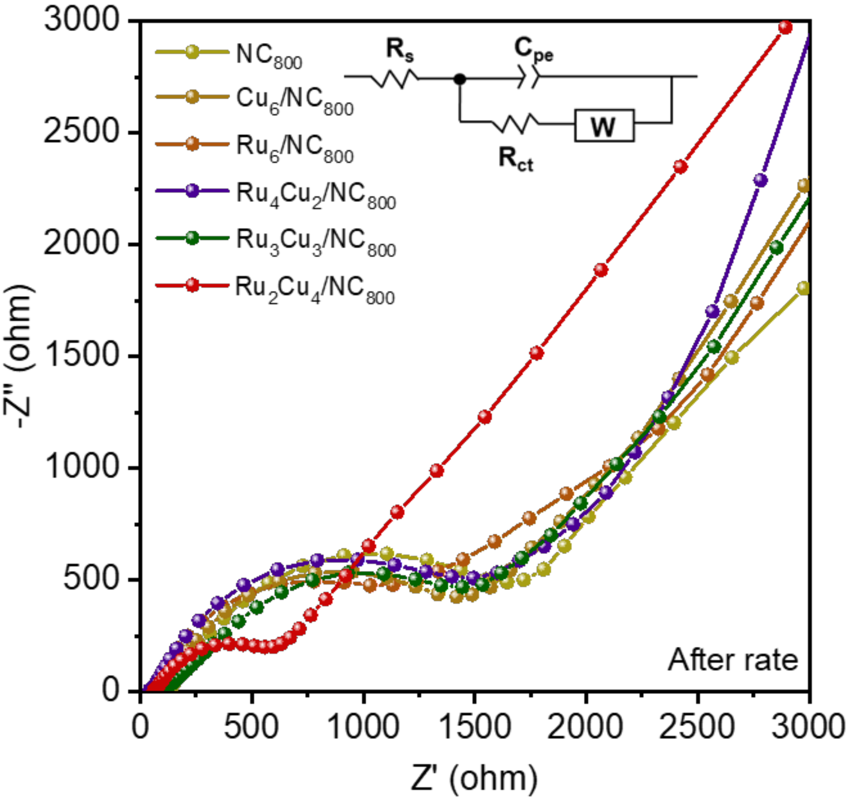

Figure S34. Electrochemical impedance spectra of different cathodes after rate.

Figure S35

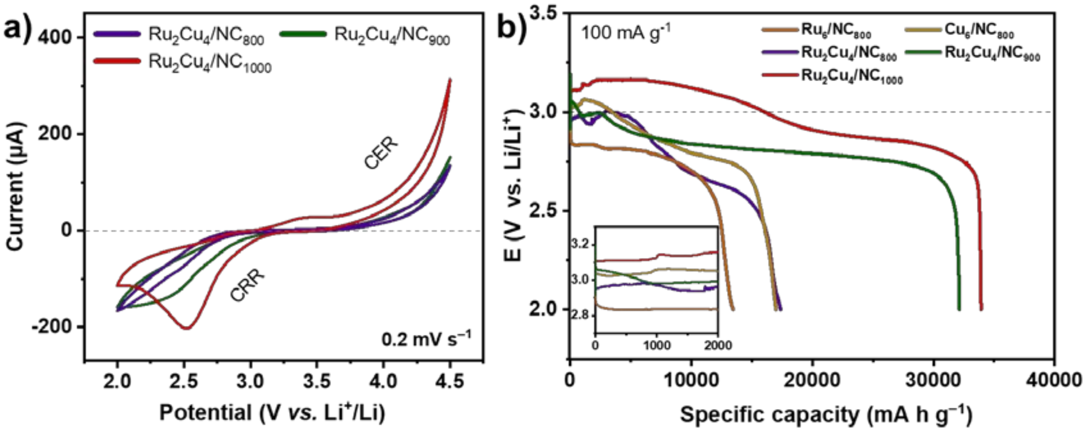

Figure S35. a) CV curves at 0.2 mV s^{-1} of Ru_2Cu_4/NC_{800}, Ru_2Cu_4/NC_{900}, and Ru_2Cu_4/NC_{1000}. **b)** Full discharge curves of Ru_6/NC_{800}, Cu_6/NC_{800}, Ru_2Cu_4/NC_{800}, Ru_2Cu_4/NC_{900}, and Ru_2Cu_4/NC_{1000} materials at a current density of 100 mA g^{-1}.

As shown in Figure S35b, Ru_2Cu_4/NC_{1000} delivers both a higher discharge capacity and a higher discharge plateau than the other catalysts. This behavior reflects its high CO_2 reduction reaction activity and suggests that the possible reaction pathway may differ from those of the other catalysts. A detailed discussion of the associated reaction mechanism is provided in the following section.

Figure S36

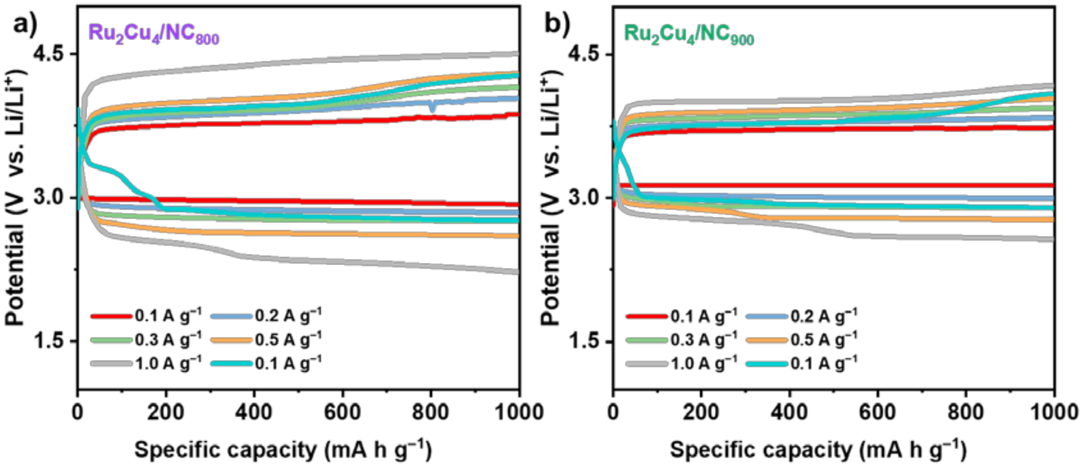

Figure S36. Charge and discharge curves of Ru_2Cu_4/NC_{800} and Ru_2Cu_4/NC_{900} materials taken at different current densities.

Figure S37

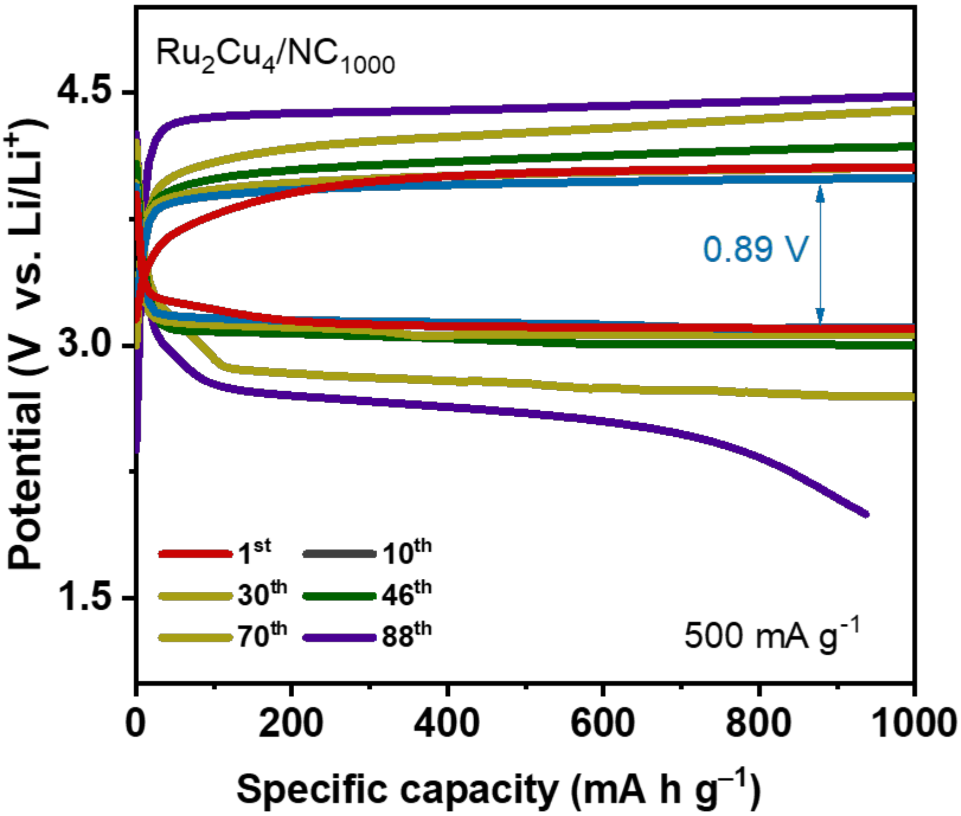

Figure S37. Charge and discharge curves of Ru_2Cu_4/NC_{1000} catalyst at current densities of 500 mA g^{-1}.

Figure S38

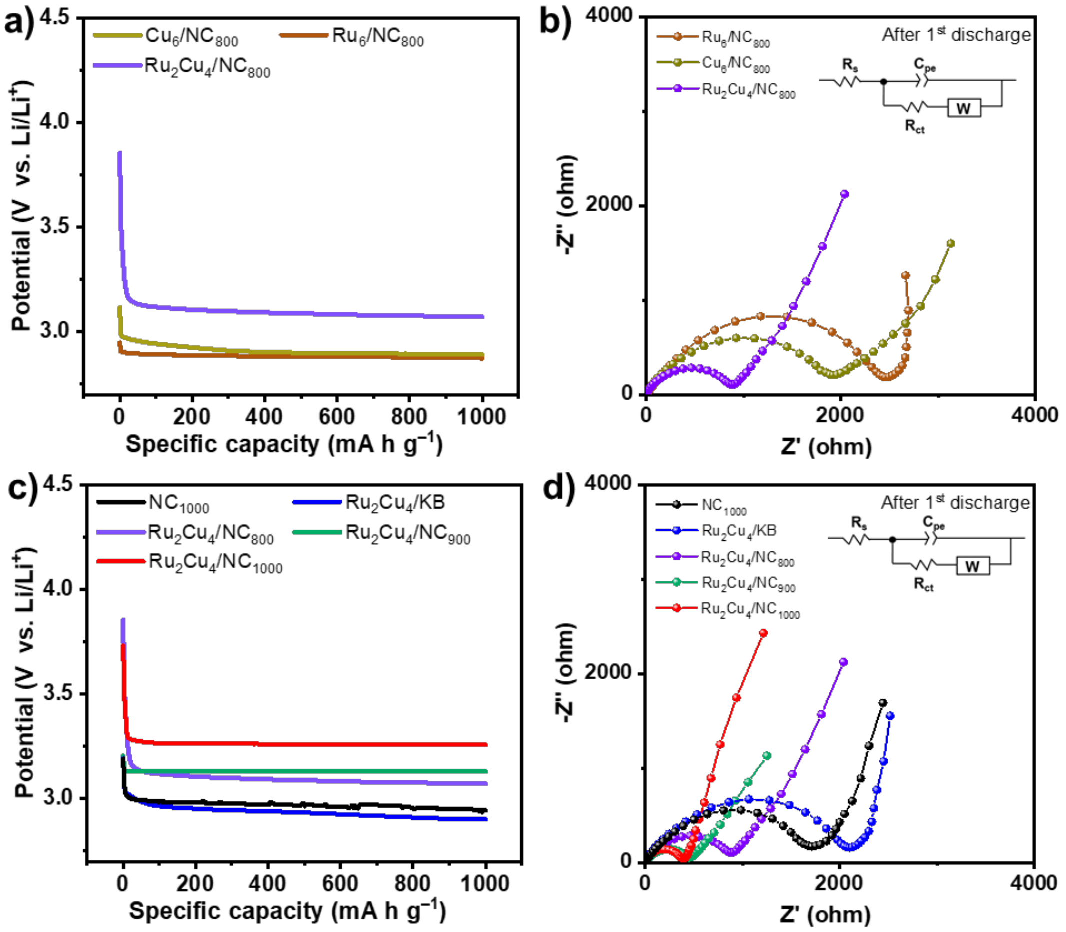

Figure S38. a) Discharge curves of Ru_6/NC_{800}, Cu_6/NC_{800}, and Ru_2Cu_4/NC_{800} catalysts taken at current densities of 100 mA g^{-1}. **b**) EIS of Ru_6/NC_{800}, Cu_6/NC_{800}, and Ru_2Cu_4/NC_{800} cathodes after discharge. **c)** Discharge curves of NC_{1000}, Ru_2Cu_4/KB, Ru_2Cu_4/NC_{800}, Ru_2Cu_4/NC_{900}, and Ru_2Cu_4/NC_{1000} catalysts taken at current densities of 100 mA g^{-1}. **d**) EIS of NC_{1000}, Ru_2Cu_4/KB, Ru_2Cu_4/NC_{800}, Ru_2Cu_4/NC_{900}, and Ru_2Cu_4/NC_{1000} cathodes after discharge.

Figure S39

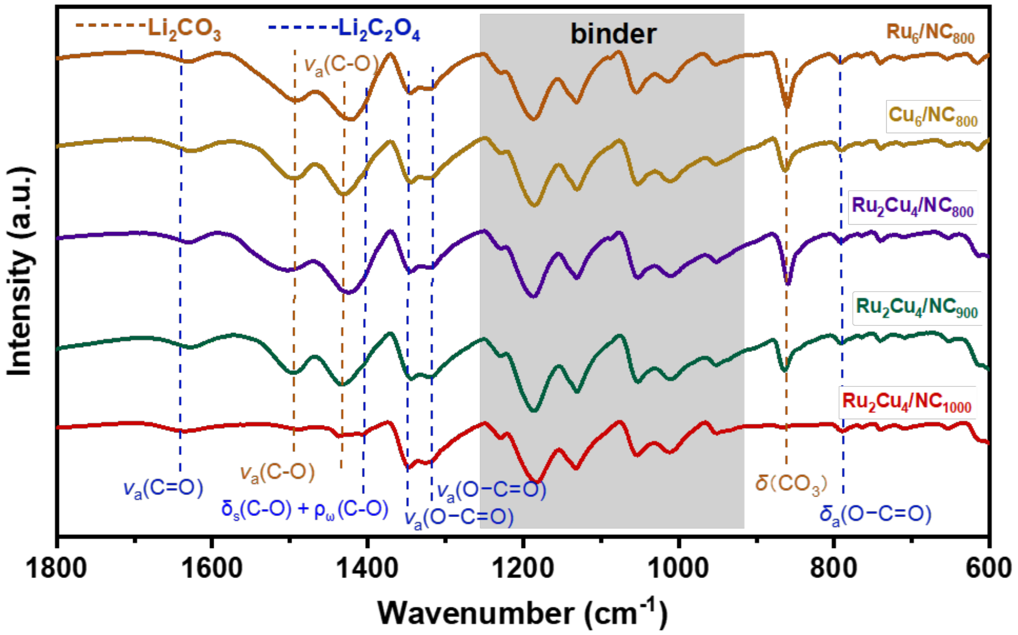

Figure S39. FTIR of Ru_6/NC_{800}, Cu_6/NC_{800}, Ru_2Cu_4/NC_{800}, Ru_2Cu_4/NC_{900}, and Ru_2Cu_4/NC_{1000} cathodes after discharge taken at current densities of 100 mA g^{-1} with limited capacity of 1000 mA g^{-1}.

Figure S40

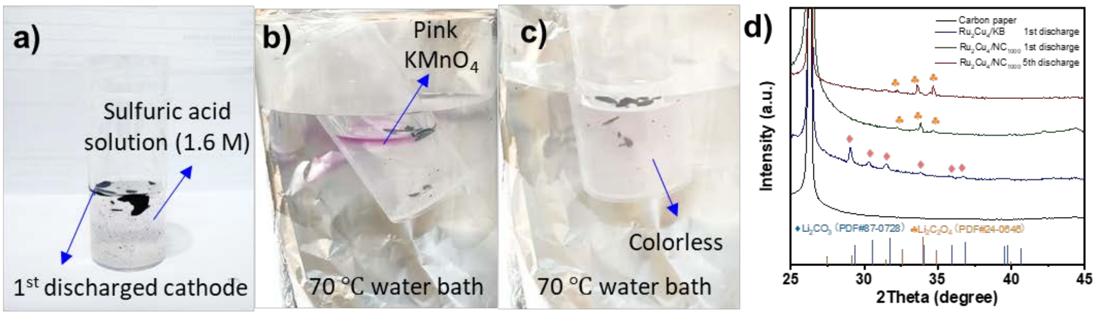

Figure S40. a-c) Photograph of the lithium oxalate titration of the cathode after the first discharge. **d**) XRD patterns of 1st discharged products.

To further solidate our findings in the engineering of CO_2 reduction pathway using our Ru_2Cu_4/NC_{1000} catalyst, we used a chemical titration process to quantitatively identify the existence of $Li_2C_2O_4$ on the discharged cathodes. Following the discharge process, the Ru_2Cu_4/NC_{1000} electrodes were collected in an argon filled glovebox and washed thoroughly with a volatile aprotic solvent to remove all residual salt and electrolyte. The electrodes were then immersed in a sulfuric acid solution (1.6 M), which successfully dissolved the solid discharge products into the aqueous phase. We subsequently performed a rigorous redox titration using a standardized potassium permanganate ($KMnO_4$, 0.0021 M) at 70 ℃, which was calibrated against sodium oxalate (0.0029 M). As shown in Figure S40b, upon the addition of $KMnO_4$, the solution became colorless due to the reduction of MnO_4^- (Figure S40c). After the addition of approximately 700 µL of $KMnO_4$, a persistent pale pink color appeared and remained for at least 30 s, indicating the complete consumption of oxalate species. This observation confirms the presence of lithium oxalate on the electrode.

Because carbonate species (CO_3^{2-}) are already fully oxidized and cannot react with $KMnO_4$, the quantitative consumption of the $KMnO_4$ titrant is exclusively driven by the oxidation of the dissolved oxalate species ($H_2C_2O_4$) according to the following equation:

$$5H_2C_2O_4 + 2KMnO_4 + 3H_2SO_4 \rightarrow 10CO_2 + 2MnSO_4 + K_2SO_4 + 8H_2O$$

Figure S41

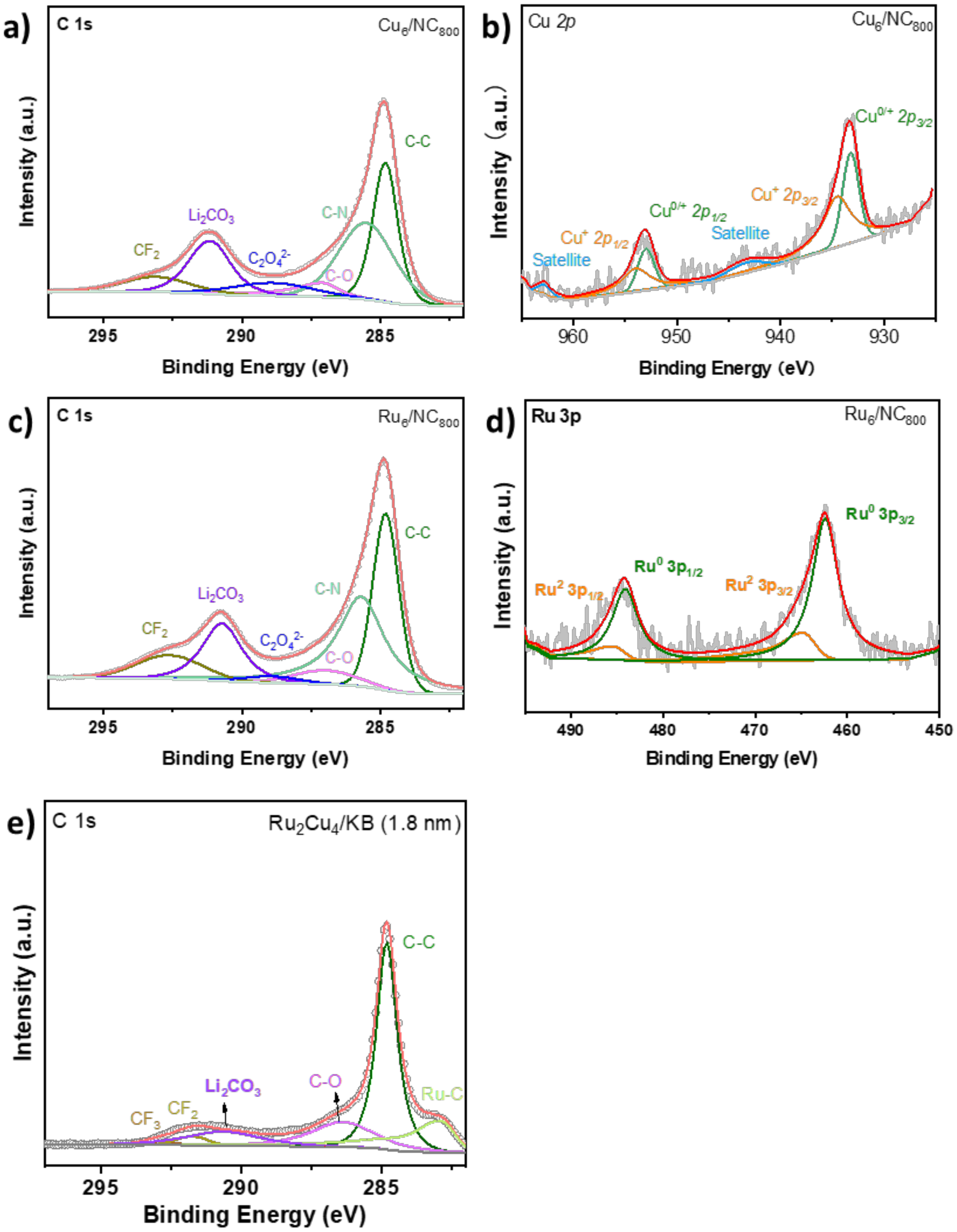

Figure S41. High-resolution XPS spectra of materials after discharge. **a**) C *1s* and **b**) Cu *2p* of Cu_6/NC_{800}. **c**) C *1s* and **d**) Ru *3p* of Ru_6/NC_{800}. **e**) C *1s* of Ru_2Cu_4/KB (1.8 nm).

XPS analysis of the discharged products (after 1st discharging, Figure S41e) reveals that no peaks at around 289. 4 eV can be indexed to $C_2O_4^{2-}$, indicating that the Li_2CO_3 remains the dominant discharge product on Ru_2Cu_4/KB (1.8 nm).

Figure S42

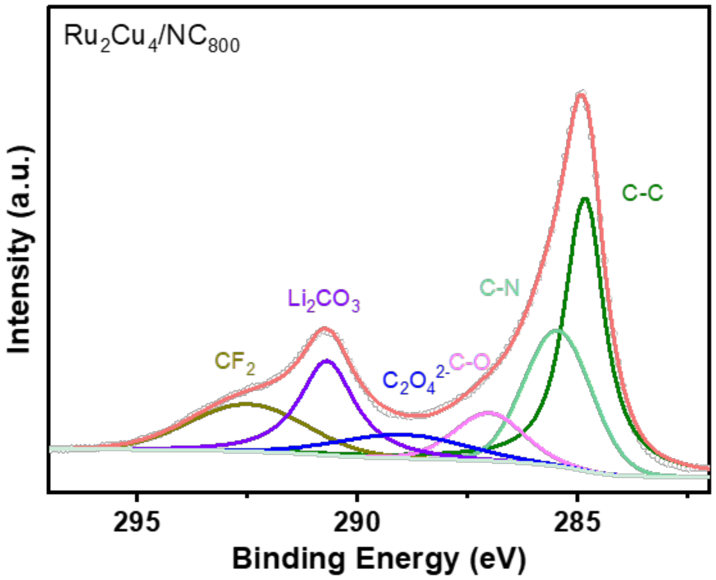

Figure S42. High-resolution XPS C *1s* spectra of Ru_2Cu_4/NC_{800} materials after discharge.

Figure S43

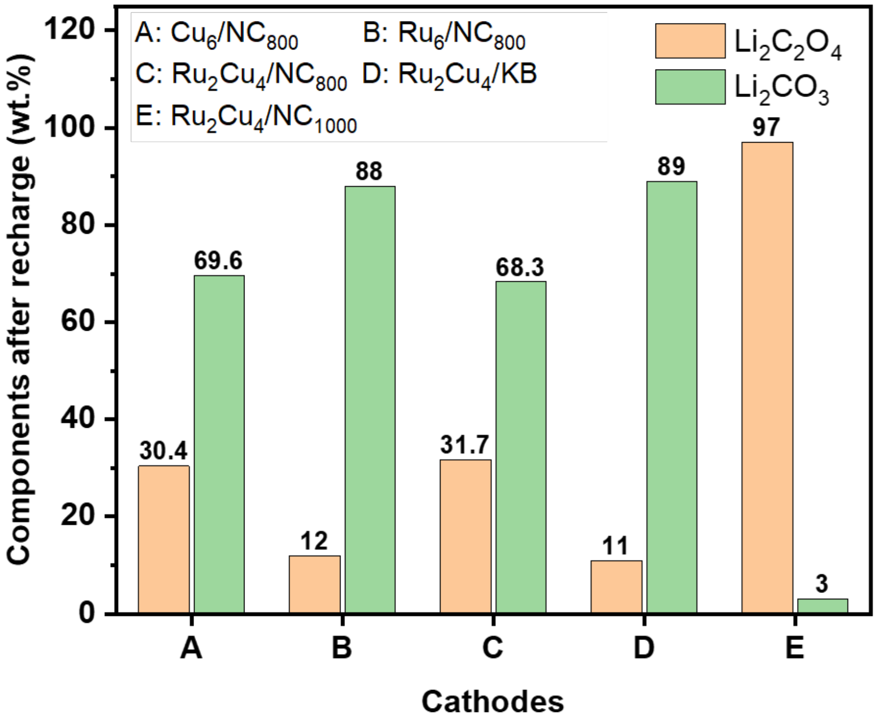

Figure S43. Comparison diagram of cathode surface components ($Li_2C_2O_4$/Li_2CO_3) after discharge.

Figure S44

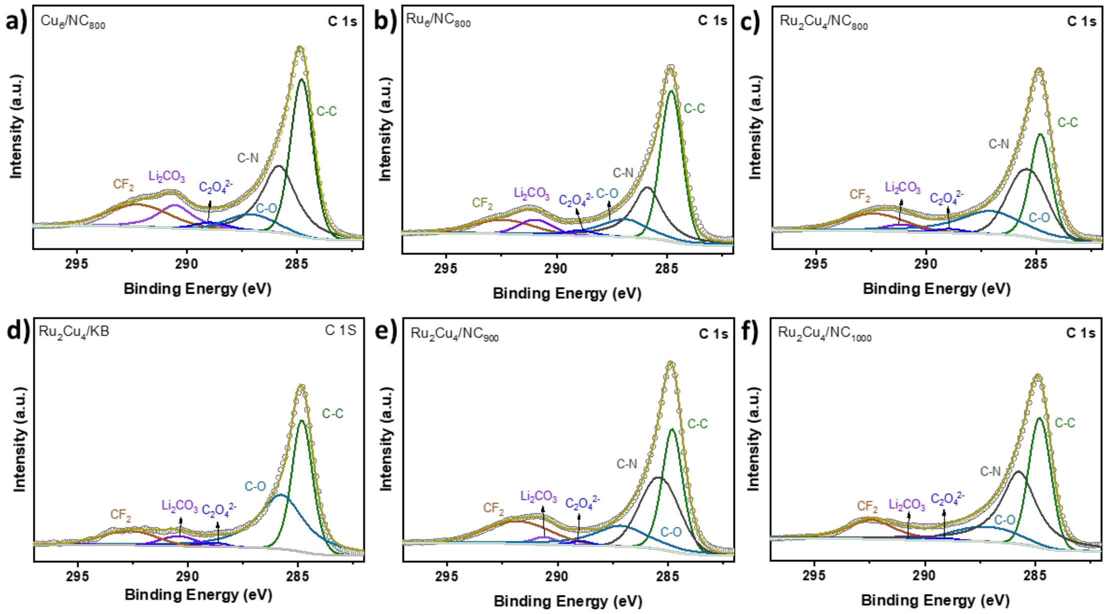

Figure S44. XPS C *1s* spectra of **a**) Cu_6/NC_{800}, **b**) Ru_6/NC_{800}, **c**) Ru_2Cu_4/NC_{800}, **d**) Ru_2Cu_4/KB, **e**) Ru_2Cu_4/NC_{900}, and f) Ru_2Cu_4/NC_{1000} cathodes after recharge.

Figure S45

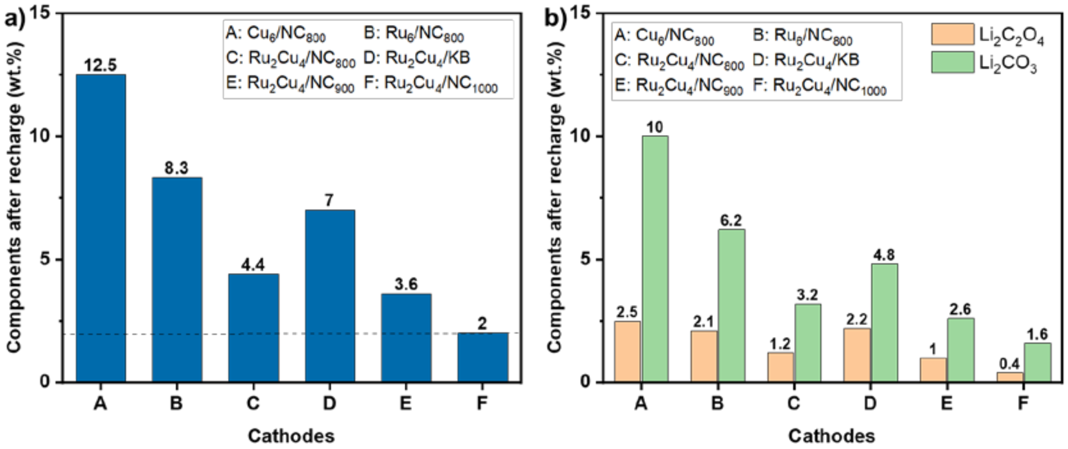

Figure S45. Comparison diagram of **a)** discharged products and **b)** $Li_2C_2O_4/Li_2CO_3$ after recharge.

Figure S46

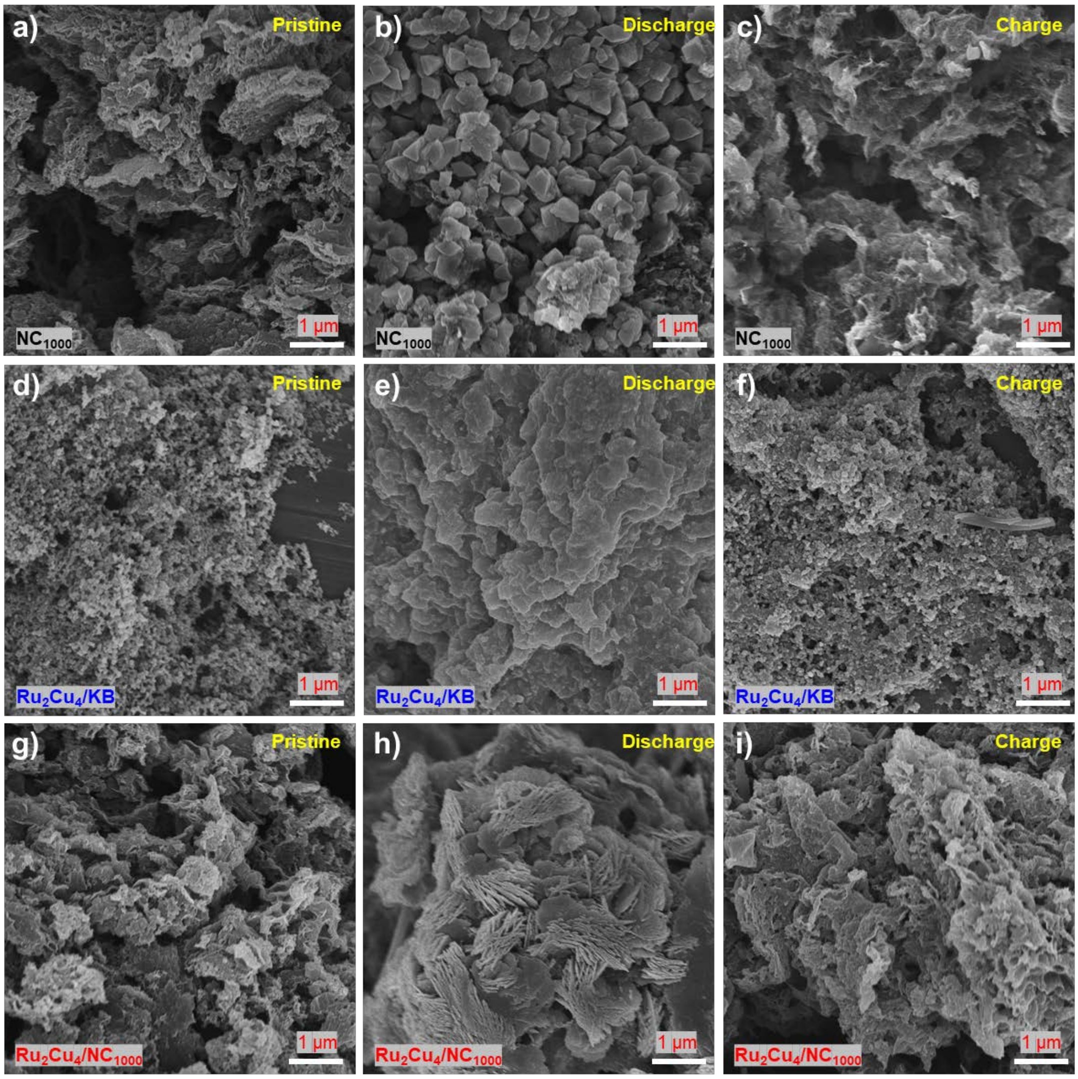

Figure S46. SEM image of the **a-c**) NC_{1000}, **d-f**) Ru_2Cu_4/KB, and **g-i**) Ru_2Cu_4/NC_{1000} cathodes at pristine, discharged and charged stages.

Figure S47

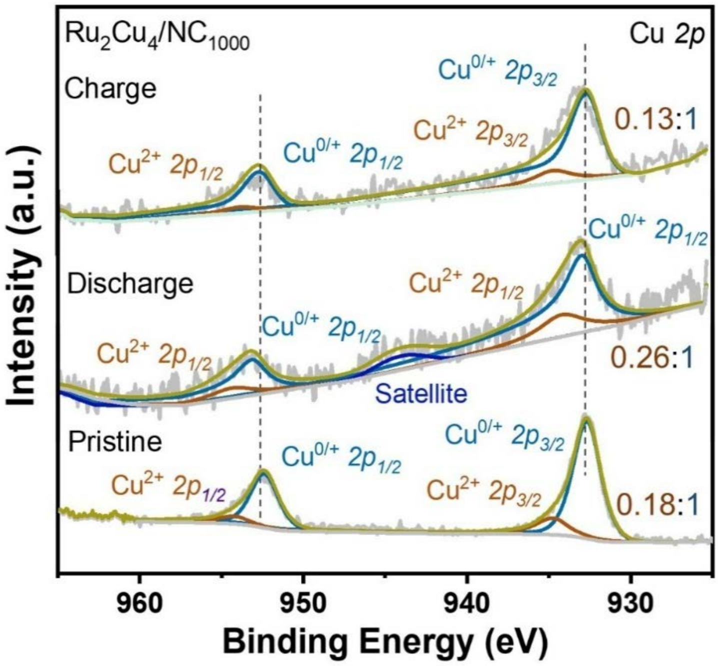

Figure S47. High-resolution XPS spectra of materials with pristine and after the 1st discharge/recharge of Cu 2*p* of Ru_2Cu_4/NC_{1000}.

Figure S48

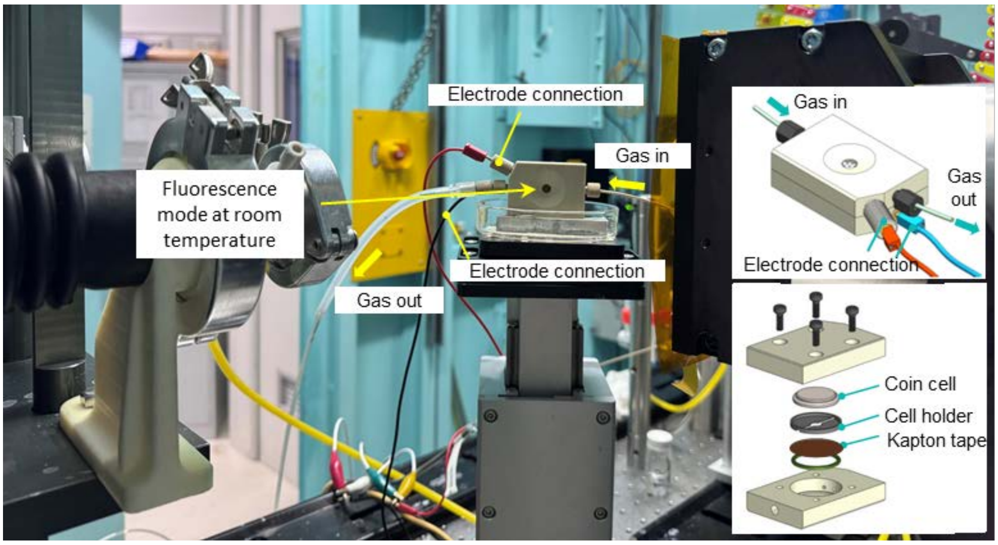

Figure S48. Schematic of *in-situ* XAS cell.

For in-situ XAS testing, CR2032 coin cells (modified with a hole on the cathode side) were assembled inside a CO_2-filled glove box. The cells included air electrodes and lithium chip anodes, separated by a glass fiber separator (Whatman, 19 mm diameter). A 1 M LiTFSI/DMSO solution served as the electrolyte. The assembled cells were sealed within the *in-situ* XAS setup and continuously purged with CO_2 gas throughout the testing process.

Figure S49

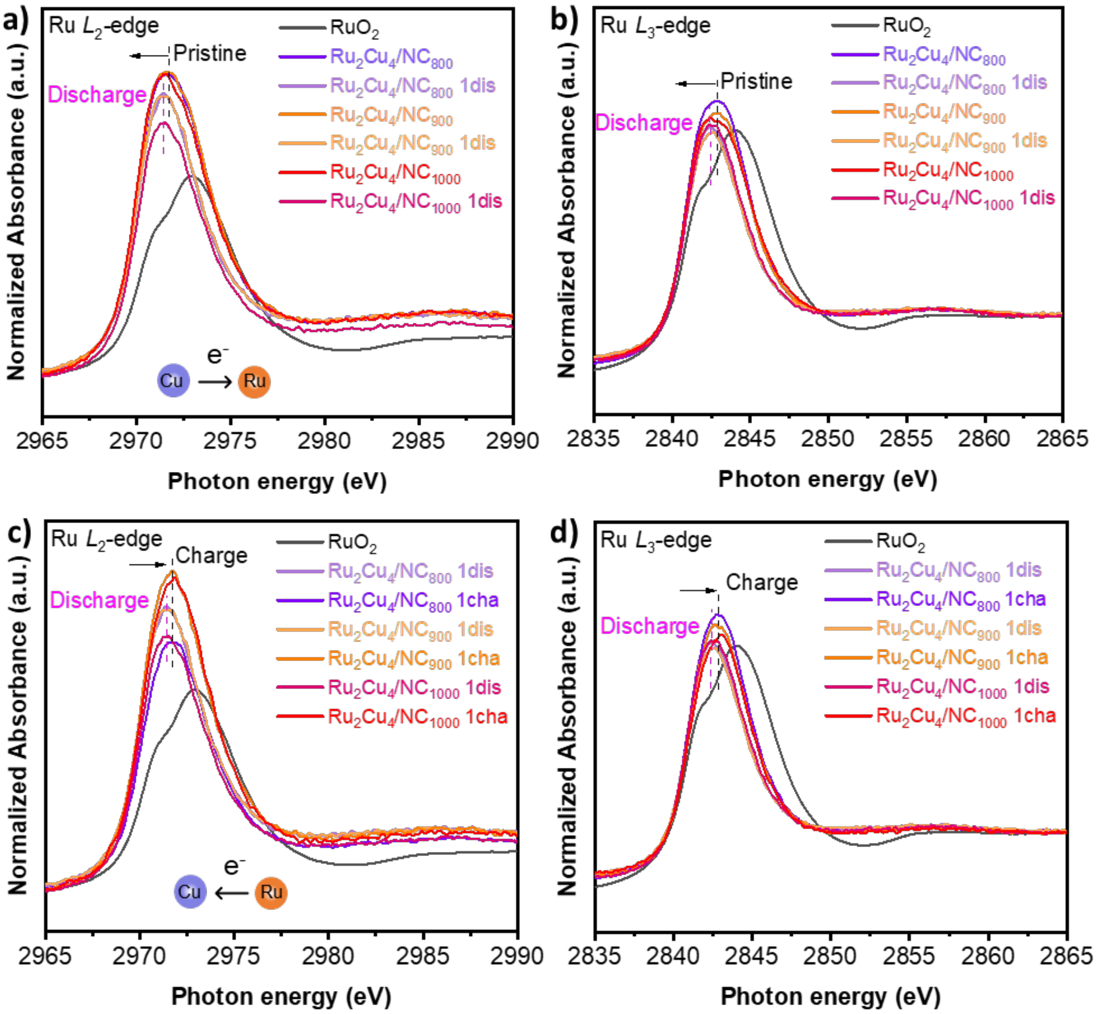

Figure S49. a, b) Ru L_2-edge and Ru L_3-edge spectra of Ru_2Cu_4/NC_{800}, Ru_2Cu_4/NC_{900}, and Ru_2Cu_4/NC_{1000} at pristine and discharged state, and RuO_2. **c, d**) Ru L_2-edge and Ru L_3-edge spectra of Ru_2Cu_4/NC_{800}, Ru_2Cu_4/NC_{900}, and Ru_2Cu_4/NC_{1000} at discharged and charged state, and RuO_2.

During discharge, the Ru L_2-edge and L_3-edge spectra shifted to lower energy levels relative to the pristine state, signifying electron accumulation at Ru sites and enhanced adsorption of CO_2 and discharge intermediates. Conversely, during the charging process, the Ru L_2-edge and L_3-edge spectra shifted to higher energy, reflecting the oxidation of Ru driven by interactions with charge intermediates and discharge products.

Figure S50

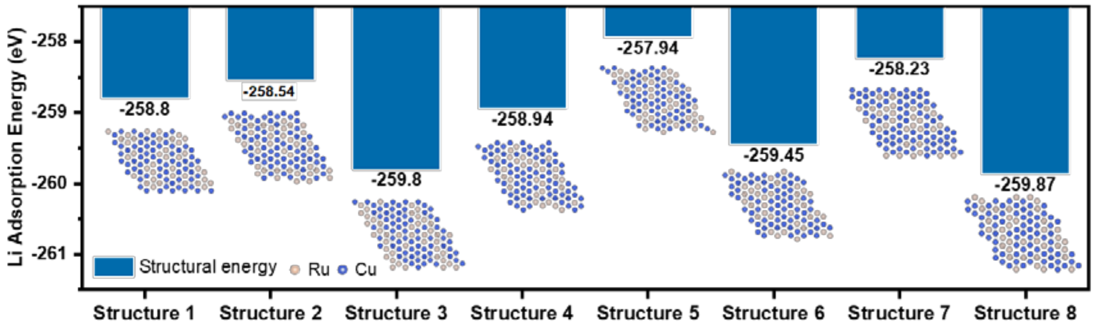

Figure S50. Structural energies of Ru_2Cu_4 clusters.

To preserve the intrinsic Cu *fcc* crystal symmetry, we constructed the Ru–Cu alloy model by slicing a Cu *fcc* cell along the (111) facet and expanding it to a 3 × 3 × 2 slab with a vacuum layer over 15 Å. Eight quasi-random Ru: Cu = 1: 2 substitutional configurations were generated, with Ru and Cu occupying different lattice sites. Total-energy comparison shows that Structure 8 possesses the lowest energy, and therefore it was selected for subsequent Ru_2Cu_4-C and Ru_2Cu_4-NC model construction.

Figure S51

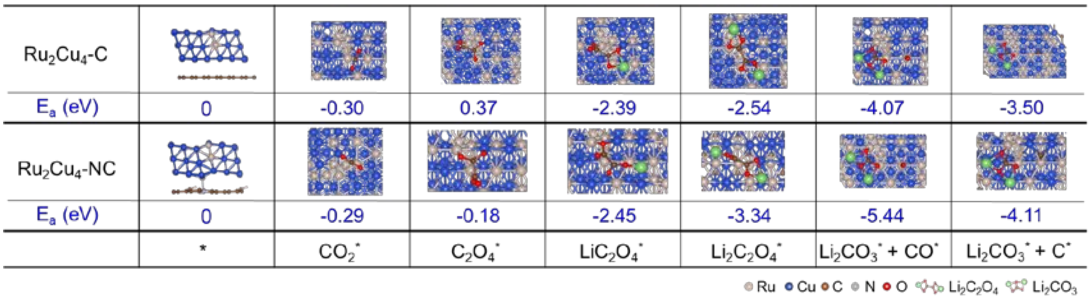

Figure S51. Adsorption configurations at Ru_2Cu_4-C and Ru_2Cu_4-NC.

Figure S52

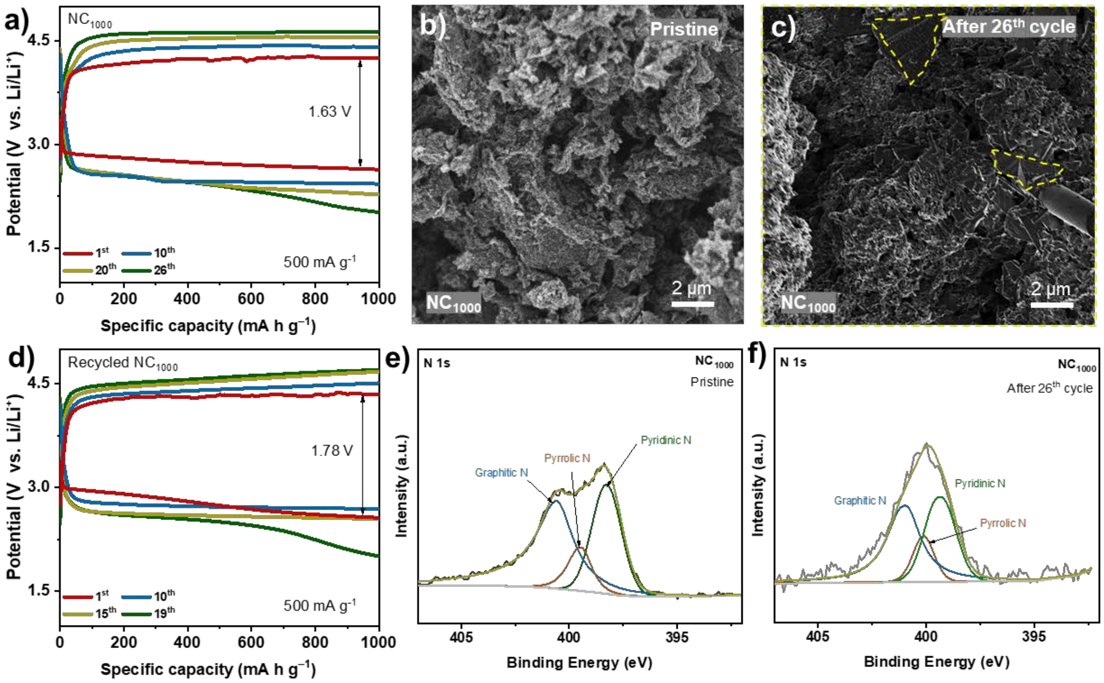

Figure S52. a) The long cyclic performance of NC_{1000} cathode at 500 mA g^{-1} curtailed the specific capacity of 1000 mAh g^{-1}. SEM images of **b)** pristine and **c)** after 26th cycle cathodes at 500 mA g^{-1} with the capacity of 1000 mAh g^{-1}. **d)** The long cyclic performance of recycled NC_{1000} cathode after 26th at 500 mA g^{-1} curtailed the specific capacity of 1000 mAh g^{-1}. High-resolution XPS spectra of N 1*s* of **e)** pristine $NC_{1000}$and **f)** after 26th cycle cathodes.

As shown in Figure S52a, NC_{1000} exhibits stable cycling performance at 500 mA g^{-1} with a limited specific capacity of 1000 mAh g^{-1}, sustaining 26 cycles before reaching the cut-off voltage of 2.0 V. The initial overpotential is ~1.63 V. After prolonged cycling, SEM analysis reveals that the cathode largely recovers its original morphology and remains similar to the pristine state. However, some residual discharge products (highlighted in the yellow circle) are still present, indicating incomplete decomposition during cycling (Figure S52c).

To further verify the stability of NC_{1000}, the cycled cathode was reassembled into a new cell and tested under identical conditions. As shown in Figure S52d, the initial overpotential (~1.78 V) is comparable to that of the fresh cathode, suggesting that the NC_{1000} support maintains its structural integrity. The slightly reduced cycle life (19 cycles vs. 26 cycles for the fresh NC_{1000}) is likely due to the accumulation of residual discharge products rather than degradation of the catalyst itself. In addition, side reactions associated with electrolyte decomposition or Li anode instability may also contribute to cell failure. Furthermore, XPS analysis (Figure S52e,f) shows that the N chemical states remain largely unchanged after prolonged cycling, confirming the good structural and chemical stability of NC_{1000}. The positive shift and changes in peak shape observed in the N 1s spectra after cycling can be attributed to the combined effects of residual discharge products (e.g., Li_2CO_3), electrolyte decomposition (LiTFSI, containing $–SO_2–N–SO_2–$ groups, and DMSO), and surface oxidation. These factors introduce electron-withdrawing species and increase the oxidation state of surface functional groups, leading to a decrease in electron density and a corresponding shift toward higher binding energies. In addition, surface coverage by insulating species may also contribute to charging effects during XPS measurement.[20] These additional results have been incorporated into the

R-SI to provide a more comprehensive evaluation of catalyst stability. The results indicate that the overall morphology and chemical structure of NC_{1000} remain largely unchanged after cycling, suggesting good stability of the support.

Table S1. The synthesis of RuCu/KB or NC materials.

No.	Catalysts	metal precursor/mmol	Carbon/mg
1	Cu_6/KB (NC_{800})	$CuSO_4 \cdot 5H_2O$/0.06	20
2	Ru_6/KB (NC_{800})	$RuCl_3 \cdot xH_2O$/0.06	20
3	Ru_4Cu_2/KB (NC_{800})	$RuCl_3 \cdot xH_2O$/0.04<br>$CuSO_4 \cdot 5H_2O$/0.02	20
4	Ru_3Cu_3/KB (NC_{800})	$RuCl_3 \cdot xH_2O$/0.03<br>$CuSO_4 \cdot 5H_2O$/0.03	20
5	Ru_2Cu_4/KB (NC_{800}, NC_{900}, or NC_{1000})	$RuCl_3 \cdot xH_2O$/0.02<br>$CuSO_4 \cdot 5H_2O$/0.04	20

Table S2. Elemental compositions of samples

Elements<br>Samples	Weight ratio (%)		
	**C**	**N**	**O**
NC_{800}	75.87	14.18	9.95
NC_{900}	78.92	8.68	12.40
NC_{1000}	82.37	6.12	11.51

Note: C, N, and O weight percent were measured by XPS.

Table S3. The inductively coupled plasma-atomic (ICP) emission spectroscopy results of Ru_2Cu_4/KB and Ru_2Cu_4/NC_{1000} materials.

No.	Catalysts	Cu (wt %)	Ru (wt %)	Cu: Ru (molar ratio)
1	Ru_2Cu_4/KB	8.77	6.46	2.16: 1
2	Ru_2Cu_4/NC_{1000}	7.37	5.69	2.06: 1

Table S4. EXAFS fitting results for different materials at Ru and Cu K-edge.

	Materials	*pair*	*CN*	*R (Å)*	σ^2 ($\times 10^{-3}$Å^2)	ΔE_0(eV)	R-factor
Ru	Ru foil	Ru-Ru	12.0	2.675	3.6±0.5	-5.4±0.8	0.016
	Ru_2Cu_4/KB	Ru-Cu	2.0	2.639	4.9±0.8	-6.9±1.1	0.014
		Ru-Ru	8.0±1.2	2.669	21.9±20.1		
	Ru_2Cu_4/CN_{1000}	Ru-N	1.4±0.3	2.001	1.8±1.2	-6.8±1.0	0.007
		Ru-Cu	3.7	2.609	21.1±3.2		
		Ru-Ru	3.0	2.655	8.5±0.5		
Cu	Cu foil	Cu-Cu	12.0	2.543	8.5±0.4	5.6±0.4	0.003
	Ru_2Cu_4/CN_{1000}	Cu-N	0.6±0.6	1.882	2.4±0.6	4.9±0.8	0.012
		Cu-Cu	6.4±1.0	2.540	9.2±0.1		
		Cu-Ru	1.5	2.674	6.2±0.5		

CN - Coordination number

R - Interatomic distance

σ^2 - Debye-Waller factor (thermal and static disorder in absorber-scatterer distances)

ΔE_0 - Edge energy shift (the difference between the zero kinetic energy value of the sample and that of standard theoretical model)

R factor - Goodness of fitting

Table S5. Cycling performance of cathodes at different current densities

Cathodes	Discharge/charge termination voltage (V)				
	Current density 100 mA g^{-1}	Current density 200 mA g^{-1}	Current density 500 mA g^{-1}	Current density 1000 mA g^{-1}	Current density 100 mA g^{-1}
NC_{1000}	2.95/4.00	2.87/4.16	2.49/4.29	2.19/4.41	2.77/4.15
Ru_2Cu_4/KB	2.90/3.87	2.80/4.00	2.66/4.19	2.49/4.24	2.86/4.05
Ru_2Cu_4/NC_{800}	2.93/3.87	2.84/4.04	2.60/4.30	2.22/4.50	2.76/4.28
Ru_2Cu_4/NC_{900}	3.13/3.74	3.00/3.84	2.78/4.04	2.57/4.18	2.90/4.09
Ru_2Cu_4/NC_{1000}	3.25/3.75	3.14/3.90	3.07/4.10	2.96/4.22	3.16/3.94

Table S6. Cycling performance of cathodes versus Ru loading on catalysts reported in recent literatures.

Cathodes	Overpotential V (current density mA g^{-1} /cut-off capacity mAh g^{-1})	Discharge capacity mAh g^{-1} (current density mA g^{-1})	Cycle stability hours (current density mA g^{-1} /cut-off capacity mAh g^{-1})	Electrolytes
SA Ru-Co_3O_4/CC[21]	1.05 (100/500)	30915 (100)	>2000 (200/800)	1 M LiTFSI/TEGDME
SA Ru_h-NC@rGO[22]	0.97 (100/1000)	44700 (100)	734 (1000/1000)	1 M LiTFSI/TEGDME
Ru_{AC+SA}@ NC[23]	1.05 (100/1000)	10651.9 (100)	≈230 (300/500)	1 M LiTFSI/TEGDME
Ru/Co-CPY@CNT-2[24]	0.84 (100/1000)	24740 (200)	720 (500/1000)	1 M LiTFSI/TEGDME
Ru/NS-G[25]	1.13 (100/1000)	10660 (100)	100 (250/500)	1 M LiTFSI/TEGDME
Cu(I) RM and Ru catalysts[26]	1.03 (100/1000)	5846 (100)	4000 (100/1000)	0.1 M $LiClO_4$/MeCN
This work RuCu/NC	0.50 (100/1000)	33922 (100)	1800 (100/1000)	1 M LiTFSI/DMSO

Table S7. D-band center of Ru_2Cu_4-C and Ru_2Cu_4-NC.

Models	Cu (spin up)	Ru (spin up)	Cu (spin down)	Ru (spin down)
Ru_2Cu_4-C	-2.472	-1.178	-2.473	-1.182
Ru_2Cu_4-NC	-2.390	-1.049	-2.390	-1.049

Note: The units of the d-band center are all eV *vs* Fermi Level.

References

[1]W. Gao, K. Xie, J. Xie, X. Wang, H. Zhang, S. Chen, H. Wang, Z. Li, C. Li, *Adv. Mater.* **2023**, 35, e2202952.

[2]J. Zou, G. Liang, J. A. Yuwono, F. Zhang, Y. Fan, S. Zhang, B. Johannessen, L. Sun, Z. Guo, *ACS Energy Lett.* **2024**, 9, 5145.

[3]G. Kresse, J. Furthmuller, *Phys Rev B Condens Matter* **1996**, 54, 11169.

[4]J. P. Perdew, K. Burke, M. Ernzerhof, *Phys Rev Lett* **1996**, 77, 3865.

[5]M. Ernzerhof, G. E. Scuseria, *The Journal of Chemical Physics* **1999**, 110, 5029.

[6]S. Grimme, S. Ehrlich, L. Goerigk, *J Comput Chem* **2011**, 32, 1456.

[7]S. Grimme, J. Antony, S. Ehrlich, H. Krieg, *J Chem Phys* **2010**, 132, 154104.

[8]K. Momma, F. Izumi, *Journal of Applied Crystallography* **2011**, 44, 1272.

[9]V. Wang, N. Xu, J.-C. Liu, G. Tang, W.-T. Geng, *Computer Physics Communications* **2021**, 267.

[10]X. Liu, L. Li, W. Zhou, Y. Zhou, W. Niu, S. Chen, *ChemElectroChem* **2015**, 2, 803.

[11]J. Wang, Z. Wei, S. Mao, H. Li, Y. Wang, *Energy Environ. Sci.* **2018**, 11, 800.

[12]G. Witjaksono, M. Junaid, M. H. Khir, Z. Ullah, N. Tansu, M. Saheed, M. A. Siddiqui, S. S. Ba-Hashwan, A. S. Algamili, S. A. Magsi, M. Z. Aslam, R. Nawaz, *Molecules* **2021**, 26.

[13]Z. R. Ismagilov, A. E. Shalagina, O. Y. Podyacheva, A. V. Ischenko, L. S. Kibis, A. I. Boronin, Y. A. Chesalov, D. I. Kochubey, A. I. Romanenko, O. B. Anikeeva, T. I. Buryakov, E. N. Tkachev, *Carbon* **2009**, 47, 1922.

[14]S. F. Hung, A. Xu, X. Wang, F. Li, S. H. Hsu, Y. Li, J. Wicks, E. G. Cervantes, A. S. Rasouli, Y. C. Li, M. Luo, D. H. Nam, N. Wang, T. Peng, Y. Yan, G. Lee, E. H. Sargent, *Nat. Commun.* **2022**, 13, 819.

[15]Y. Li, C. Yang, J. Yue, H. Cong, W. Luo, *Adv. Funct. Mater.* **2023**, 33, 2211586.

[16]J. D. Wiggins-Camacho, K. J. Stevenson, *J. Phys. Chem. C* **2009**, 113, 19082.

[17]K. Akada, S. Obata, K. Saiki, *ACS Omega* **2019**, 4, 16531.

[18]M. Yuan, Y. Bai, J. Zhang, T. Zhao, S. Li, H. He, Z. Liu, Z. Wang, G. Zhang, *J. Mater. Chem. A* **2020**, 8, 26066.

[19]S. Liu, H. Yang, X. Huang, L. Liu, W. Cai, J. Gao, X. Li, T. Zhang, Y. Huang, B. Liu, *Adv. Funct. Mater.* **2018**, 28.

[20]D. R. Baer, K. Artyushkova, H. Cohen, C. D. Easton, M. Engelhard, T. R. Gengenbach, G. Greczynski, P. Mack, D. J. Morgan, A. Roberts, *J. Vac. Sci. Technol. A* **2020**, 38.

[21]Z. Lian, Y. Lu, C. Wang, X. Zhu, S. Ma, Z. Li, Q. Liu, S. Zang, *Adv. Sci.* **2021**, 8, e2102550.

[22]J. Cheng, Y. Bai, Y. Lian, Y. Ma, Z. Yin, L. Wei, H. Sun, Y. Su, Y. Gu, P. Kuang, J. Zhong, Y. Peng, H. Wang, Z. Deng, *ACS Appl. Mater. Interfaces* **2022**, 14, 18561.

[23]J. Lin, J. Ding, H. Wang, X. Yang, X. Zheng, Z. Huang, W. Song, J. Ding, X. Han, W. Hu, *Adv. Mater.* **2022**, 34, e2200559.
[24]J.-H. Wang, Y. Zhang, M. Liu, G.-K. Gao, W. Ji, C. Jiang, X. Huang, Y. Chen, S.-L. Li, Y.-Q. Lan, *Cell Rep. Phys. Sci.* **2021**, 2, 100583.
[25]Y. Qiao, J. Wu, J. Zhao, Q. Li, P. Zhang, C. Hao, X. Liu, S. Yang, Y. Liu, *Energy Storage Materials* **2020**, 27, 133.
[26]X. Sun, X. Mu, W. Zheng, L. Wang, S. Yang, C. Sheng, H. Pan, W. Li, C. H. Li, P. He, H. Zhou, *Nat. Commun.* **2023**, 14, 536.
